# Synergistic assembly, disassembly, and protection of complex forms of bundled F-actin

**DOI:** 10.1083/jcb.202509039

**Published:** 2026-07-01

**Authors:** Sudeepa Rajan, Jimok Yoon, Heng Wu, Raju Baskar, Roman Aguirre, Jonathan R. Terman, Emil Reisler

**Affiliations:** 1 Department of Chemistry and Biochemistry, University of California, Los Angeles, Los Angeles, CA, USA; 2 Departments of Neuroscience and Pharmacology, The University of Texas of Southwestern Medical Center, Dallas, TX, USA; 3 Department of Microbiology, Immunology and Molecular Genetics, University of California, Los Angeles, Los Angeles, CA, USA; 4 Molecular Biology Institute, University of California, Los Angeles, Los Angeles, CA, USA

## Abstract

Actin’s transition from monomers (G-actin) to polymers (F-actin) and then into bundled and branched networks underlies many cellular and system functions. Yet, how these networks are dynamically assembled and disassembled is incompletely understood—including why F-actin is often simultaneously and redundantly bundled by different proteins. Here, we focus on fascin and espin, two bundlers that often coexist and robustly bundle F-actin. We find that they synergistically bundle F-actin compared to equal amounts of each one alone. However, we show that bundles containing these two proteins are robustly destabilized by a synergism between Mical and cofilin, indicating mechanisms of how complex bundles are disassembled and remodeled. Yet, our results also reveal that together fascin and espin protect F-actin from this disassembly more effectively than each one alone—including to regulate F-actin disassembly and cellular remodeling *in vivo*. These findings reveal mechanisms for assembling and disassembling complex networks of bundled F-actin, including a synergism between different bundlers and disassemblers in these processes.

## Teaser

Why F-actin is often simultaneously bundled together with multiple different bundling proteins and how these complex stable F-actin bundles undergo spatiotemporally targeted disassembly to drive cellular remodeling are poorly understood. Rajan et al. identify mechanisms for assembling and disassembling complex networks of multi-protein–bundled F-actin, including a synergism between different bundlers and disassemblers in these processes.

## Introduction

Many physiological functions, including motility, polarity, cell division, endocytosis, and connectivity, are controlled via rapid assembly and disassembly of the actin cytoskeleton. Actin assembly from its monomeric/globular (G) state into filamentous (F) states provides the supporting structures and dynamics needed for these diverse physiological functions (Blanchoin et al., 2014). Moreover, F-actin’s ability to be assembled into tightly packed parallel bundles further increases its stability (Rajan et al., 2023a)—including in the main core of finger-like cell protrusions (such as filopodia, stereocilia, and microvilli), and stress fibers that assist/maintain cellular adhesion and integrity (Rajan et al., 2023a). A common aspect of F-actin bundles is the presence of multiple classes of actin-bundling proteins—such as fascins, espins, fimbrin/plastins, and villins—in different combinations and molar ratios (Rajan et al., 2023a). For example, although fascin is a well-known major bundling protein, additional bundling proteins such as espins (also called ESPN, DFNB36, and forked) and fimbrin/plastins are also present in fascin-bundled F-actin structures (Rajan et al., 2023a). Therefore, fascin alone may not be optimal for their formation, maintenance, and/or function. Notably, a loss/mutation of any of these bundling proteins dramatically affects cohesive actin bundled structures (Cant and Cooley, 1996; Cant et al., 1994; Krey et al., 2016; Sekerková et al., 2011; Van Audenhove et al., 2016; Zheng et al., 2000). This underscores the importance of both individual and multiple bundling proteins in providing distinct structural and mechanical properties to bundles and tailoring them for specific cellular functions (Rajan et al., 2023a).

Stable actin bundles also need to undergo tightly regulated spatiotemporally targeted disassembly to allow cells to undergo functional remodeling (Blanchoin et al., 2014; Lappalainen et al., 2022). The molecules and mechanisms underlying this regulated disassembly are still poorly understood. Furthermore, to the best of our knowledge, the ability of disassemblers to destabilize bundles composed of multiple bundling proteins has not been examined. Recently, we found that MICAL—a potent disassembler of F-actin (Rajan et al., 2023b)—is also able to robustly disassemble fascin-bundled F-actin (Hung et al., 2010; Rajan et al., 2023c). Likewise, although cofilin, a well-known F-actin disassembler, is a relatively poor disassembler of fascin-bundled F-actin compared with Mical (Rajan et al., 2023c; and also see Breitsprecher et al., 2011; Chikireddy et al., 2024; Elkhatib et al., 2014; Schmoller et al., 2011), it robustly enhances MICAL’s disassembly of fascin-bundled F-actin, and vice versa (Rajan et al., 2023c). In light of these disassembly effects on fascin-bundled F-actin, we wondered how MICAL and cofilin affect F-actin bundled with multiple bundling proteins. For example, both fascin and espin have been well-studied *in vitro* and *in vivo* for their ability to form superbundles containing many filaments (Claessens et al., 2008; Krishnan et al., 2021; Tilney and DeRosier, 2005; Tilney et al., 1995; Wulfkuhle et al., 1998). Together, they play key roles in organizing and stabilizing actin structures in filopodia, stereocilia, microvilli, and other subcellular regions in many cell types (Chou et al., 2011; Claessens et al., 2008; Davidson et al., 2019; Dickinson and Thatcher, 1997; Krishnan et al., 2021; Okenve-Ramos and Llimargas, 2014; Tilney et al., 1995; Winkelman et al., 2016). However, the molecular, biochemical, and cellular mechanisms by which complex superbundles are disassembled and remodeled are unknown.

Thus, herein we sought to further define the role of fascin and espin in actin bundles stability, topology, and robustness—and the mechanisms through which they are disassembled. Our results confirm that while fascin and espin colocalize on actin filaments, they do not compete for binding to them and together bundle them robustly. Furthermore, we find that fascin and espin synergize to form wider, longer, and denser actin bundles than expected from the sum of their individual effects. Moreover, despite their increased stability, fascin–espin co-bundled filaments are robustly disassembled by Mical and Mical in combination with cofilin. However, compared with bundles composed of fascin or espin alone, their co-bundled filaments are more resistant to this disassembly. *In vivo* studies support our biochemical results—including that fascin and espin function together to dampen Mical’s F-actin disassembly—which is critical for controlling cellular remodeling and semaphorin repulsion. Thus, together, our results provide a first understanding of how complex F-actin bundles are dismantled. Our results demonstrate also how the presence of two different bundling proteins confers unique properties to actin filaments to help protect them from disassembly.

## Results

### Fascin and espin robustly bundle F-actin

**Figure 1.**
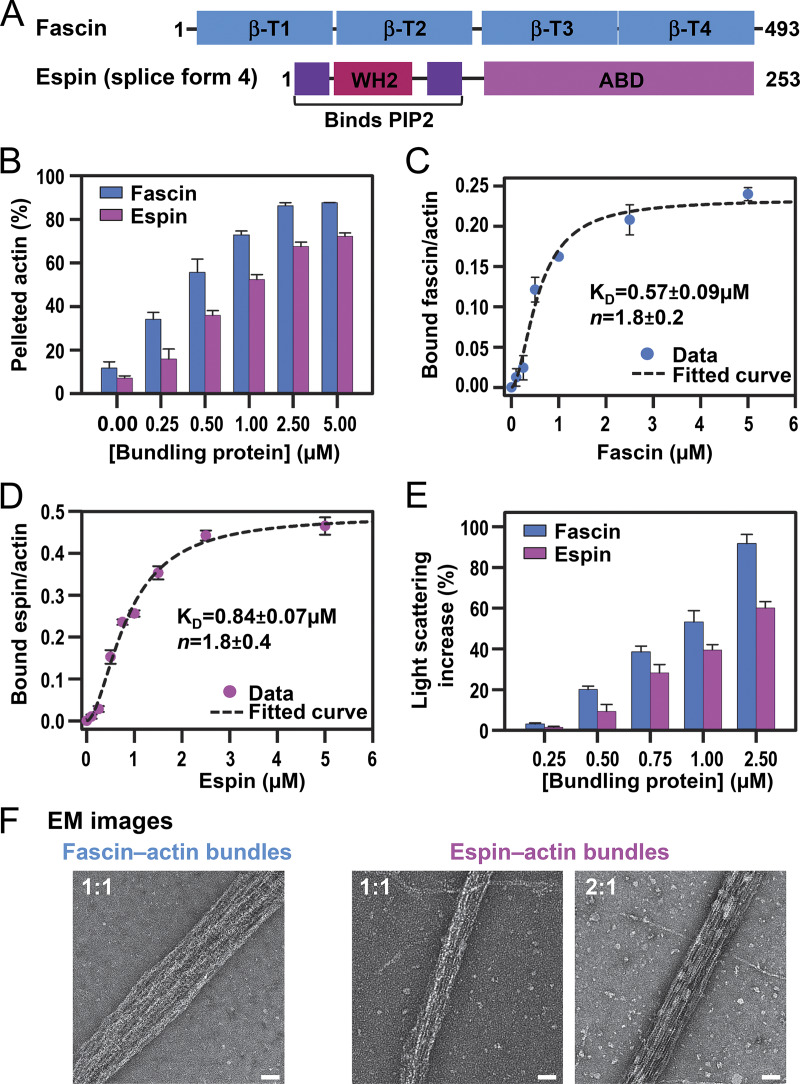
**Fascin and espin robustly bundle actin filaments. (A)** Domain structure of fascin and the espin splice form used in this study (espin-4). Fascin has four tandem β-trefoil folds (β-T) that form two ABS: ABS1 is formed by β-T1 and β-T4, and ABS2 is formed by β-T2 and β-T3 (Aramaki et al., 2016; Gong et al., 2025; Jansen et al., 2011; Song et al., 2025; Yang et al., 2013). This espin contains N-terminal phosphatidylinositol 4, 5-biphosphate (PIP2)-binding regions, a WASP homology 2 (WH2) domain, and a C-terminal ABD, which is conserved in all four splice forms of espin (Bartles, 2000; Sekerková et al., 2006). Numbers at the N (left) and C (right) termini of each diagram indicate amino acids. **(B–D)** Results of low-speed actin pelleting assays with fascin or espin (mean ± SEM). **(B)** Percentage of pelleted actin at different concentrations of bundling proteins. **(C and D)** Plots of the amount of fascin (C) or espin (D) bound to pelleted actin following their preincubation with actin (the corresponding gels are shown in Fig. S1 B). [F-actin] = 5 μM; *n* = 3 independent experiments/conditions. **(E)** Light-scattering (at λ = 325 nm) increases show increased bundling of actin filaments in the presence of different concentrations of bundling proteins (mean ± SEM). Individual plots are shown in Fig. S1 C. [F-actin] = 5 μM; *n* = 3 independent experiments/conditions. **(F)** EM images of F-actin bundles in the presence of fascin or espin. [F-actin] = 1 μM; [Fascin] and [Espin] are as indicated (by the molar ratio of bundling proteins to actin). Scale bars: 50 nm.

Fascin is a 55-kDa protein that uses its four tandem β-trefoil domains (β-T) to form two actin-binding sites (ABS), which bind and bundle two actin filaments (Fig. 1 A). Espins consist of a main C-terminal domain, which is necessary and sufficient for actin binding/bundling (ABD, Fig. 1 A), and shares homology with *Drosophila*-forked proteins (Bartles, 2000; Rajan et al., 2023a; Sekerková et al., 2006). In mammals, four splice forms (1 to 4) of the single *espin* gene have been identified (Sekerková et al., 2004). In this study, we used the shortest espin splice form—splice form 4 (∼30 kDa; also called espin-4 or small espin; Fig. 1 A)—because it contains the common espin C-terminal ABD, it has been used in foundational studies of espin (Bartles et al., 1998; Claessens et al., 2008), and it does not contain domains that serve to autoinhibit espin’s function (Liu et al., 2016; Zheng et al., 2014). Furthermore, since we have used the *Drosophila* model for *in vivo* studies herein (see below), it is notable that the espin-4 splice variant most closely matches *Drosophila* espin (forked), and it has the domains sufficient to rescue the defects that occur *in vivo* when *Drosophila* forked (*espin*) is removed (Grieshaber et al., 2001). This allowed us to more closely compare our *in vitro* and *in vivo* results. Below, for simplicity, we refer to this espin-4 splice form as “espin.”

**Figure S1.**
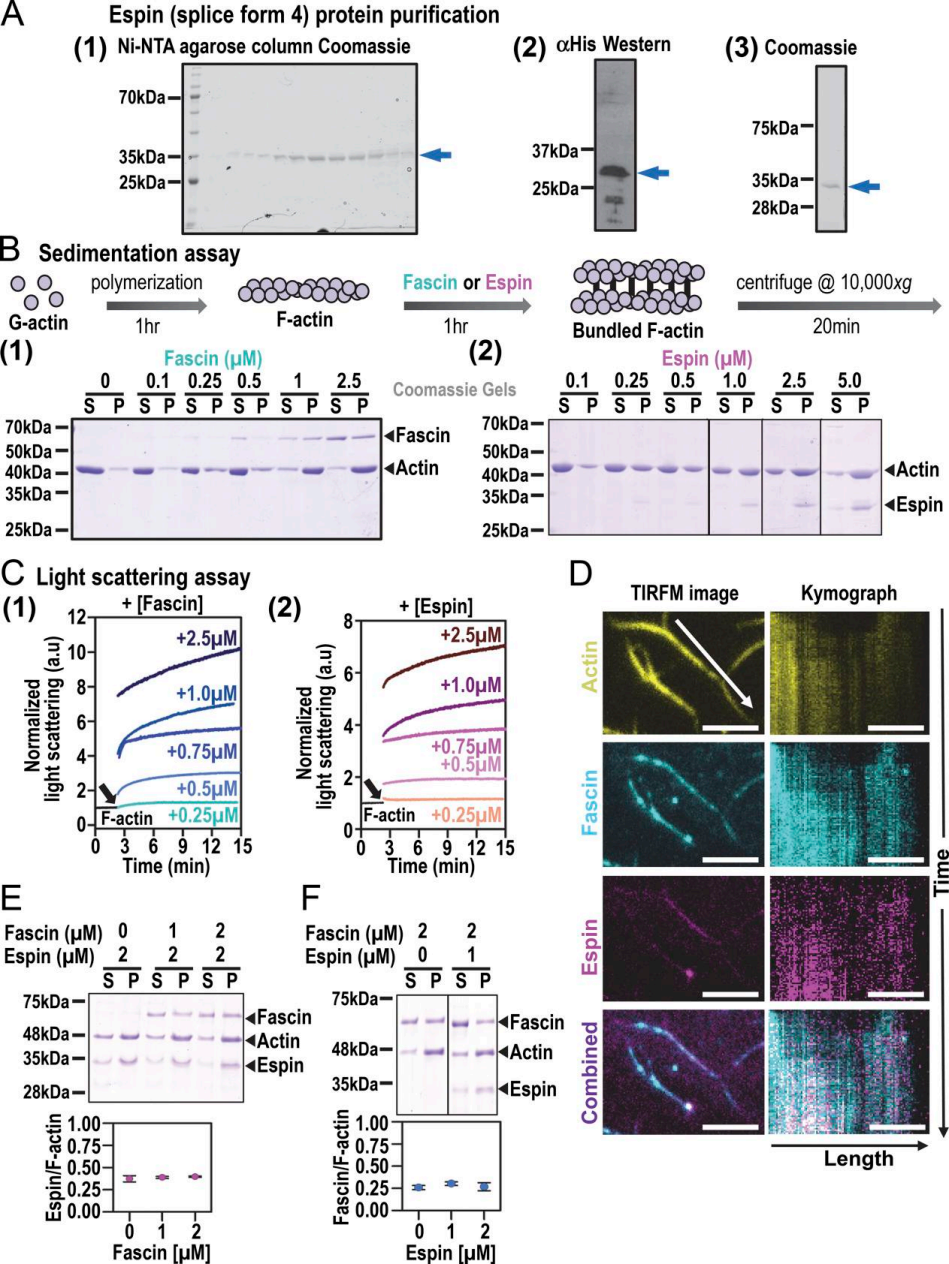
**Further analysis of fascin and espin interactions with F-actin. (A)** Purification of a recombinant espin protein. A plasmid for espin (splice form 4) was obtained (kind gift from J.R. Bartles) and then the standard protocol, as developed and used by others for purifying this protein, was followed (Bartles et al., 1998; Claessens et al., 2008; Kitajiri et al., 2010; Purdy et al., 2007). (1) Shown are different samples eluted from Nickel (Ni)-NTA affinity column using an imidazole gradient (25–250 nM) to enrich for the His-tagged espin (arrow). (2) We noted lower molecular protein bands in the purified His-tagged espin samples, which, based on a western blot using a His-tag antibody, were degraded products of full-length His-tagged espin. In light of this susceptibility of espin to degradation, we used fresh batches of espin in our experiments. (3) The enriched espin in 1 was digested with His-tagged TEV protease to cleave the His-tag. Ni-NTA beads were added to the digested sample and incubated for 1 h at 4°C to separate the cleaved espin from the uncleaved His-tagged espin and His-tagged TEV protease (which remain bound to the beads). Only the flow-through (FT) was saved and then concentrated and analyzed on SDS gels to determine espin’s purity (arrow). **(B)** Both purified fascin (1) and purified espin (2) bundle F-actin in a concentration-dependent manner. (Top diagram) To test for the ability of fascin or espin to bundle actin filaments, purified actin was polymerized, incubated with purified bundling proteins, and then subjected to a low-speed centrifugation (bundled actin filaments pellet at low centrifugation speeds [pellet fractions, P in 1 and 2]); the unbundled actin filaments and G-actin remain in the supernatant (S fractions in 1 and 2). (1 and 2) Representative (out of three independent experiments/conditions) Coomassie blue–stained SDS-polyacrylamide gels of actin filament solutions centrifuged at low-speed in the presence of fascin (1) or espin (2). [F-actin] = 5 μM; [Fascin or Espin] are as indicated in the figure. **(C)** Light-scattering measurements (at λ = 325 nm) to follow the bundling rates of actin filaments (F-actin alone, short black trace) upon addition of different concentrations of fascin (1) or espin (2). Black arrows indicate addition of bundling proteins to actin. A representative experiment (out of three independent experiments/conditions) is shown. [F-actin] = 5 μM; [Fascin or Espin] are indicated in the figure. **(D–F)** Analysis of fascin and espin binding to F-actin. **(D)** Three-color TIRFM images of F-actin bundles decorated by fascin and espin. TIRFM images (left) and the corresponding kymographs (right) of the bundles. 1 μM actin (unlabeled actin decorated with phalloidin-647, yellow), 75 nM Cy3-labeled fascin (cyan), and 75 nM Alexa488-labeled espin (magenta) were used. The kymograph was generated in the direction of the white arrow. The combined image shows fascin and espin (co-bound to actin filaments). Scale bars: 10 μm. **(E)** Espin binding to actin filaments in the presence of different fascin concentrations. Representative Coomassie-stained SDS polyacrylamide gel (top) and plot (mean values ± SEM from *n* = 3 independent experiments/conditions, bottom) show the proteins found in the supernatant (S) and pellet (P) fractions after high-speed sedimentation of the samples. [F-actin] = 5 μM; [Fascin] is shown in the figure; [Espin] = 2 μM. **(F)** Fascin binding to F-actin in the presence of different espin concentrations. Representative Coomassie-stained SDS polyacrylamide gel is shown (top) and fascin binding to actin obtained from it is on the bottom (with *n* = 3 independent experiments/conditions, and mean values ± SEM). S and P refer to supernatant and pellet fractions obtained after high-speed sedimentation of the samples. [F-actin] = 5 μM; [Fascin] = 2 μM; [Espin] is shown in the figure. Note that the gel lanes for [Fascin] = 2 μM and [Espin] = 2 μM are shown in E. Source data are available for this figure: SourceData FS1.

First, we compared fascin and espin’s effects on actin bundling by using low-speed sedimentation assays (Figs. 1, B–D; and S1, A and B). The amount of pelleted actin (bundled F-actin) increased with increasing concentrations of either fascin or espin (Fig. 1, B–D; and Fig. S1 B). However, fascin appeared to be a more potent actin bundler than espin, as it induced greater bundles formation at the same molar ratios to actin (Fig. 1 B). We found that the binding density of *Drosophila* fascin (bound fascin/actin ≈ 0.23; Fig. 1 C) aligned well with the expected fascin:actin ratio in bundles (calculated as 1/13 × 6/2 = 0.23 based on human fascin-1 structural information [Gong et al., 2025]). *Drosophila* fascin’s binding affinity (K_D_) to bundled actin (“bundling affinity”) was also similar (0.57 ± 0.09 µM) (Fig. 1 C) to published work (e.g., similar to zebrafish fascin 2b (K_d_ ≈ 0.37 µM [Chou et al., 2011]). Espin showed slightly lower actin-bundling affinity (K_D_ = 0.84 ± 0.07 µM) and an espin:actin-binding density of ∼0.45 (Fig. 1, C and D). Based on high-speed sedimentation assays (Bartles et al., 1998) and small-angle X-ray scattering (SAXS) data (from espin 3A-actin bundles) (Purdy et al., 2007), it has been reported that one espin molecule binds to ∼3–4 actin monomers. Similar to fascin, espin bundled F-actin with moderate cooperativity (Hill cooperativity coefficient [*n*] = 1.8 ± 0.2 versus 1.8 ± 0.4 for fascin) (Fig. 1, C and D).

To better monitor fascin- and espin-mediated actin bundles formation, light-scattering experiments were performed. Fascin rapidly increased light scattering in a concentration-dependent manner, indicating rapid bundle formation (Fig. 1 E and Fig. S1 C (1) [Rajan et al., 2023c]). Similarly, espin rapidly, and in a concentration-dependent manner, increased light scattering, albeit to a smaller extent than with fascin (Fig. 1 E and Fig. S1 C (2)). Indeed, at the same protein concentrations, fascin and espin exhibited notable light-scattering differences (Fig. 1 E), supporting our low-speed sedimentation results (Fig. 1 B).

To further monitor these bundles, we used transmission electron microscopy (EM). Fascin (at 1:1 molar ratio to actin) organized F-actin into bundles with a well-defined thickness (Fig. 1 F). These bundles had an average width of ∼114 nm and contained ∼18 parallel filaments, with an interfilament distance of ∼8.5 nm (Table 1). These *Drosophila* fascin–actin bundles parameters are consistent with results obtained using mammalian fascins (Ishikawa et al., 2003; Jansen et al., 2011).

**Table 1.** Parameters of different actin bundles^a^

Types of bundles	Number of filaments in bundle	Bundle width (nm)	Inter-filament distance (nm)
Fascin (1:20 actin)^b^	2 ± 0.5	9 ± 0.5	8.9 ± 0.4
Espin (1:5 actin)	5 ± 2	30 ± 14	6.9 ± 0.9
Fascin (1:20 actin) + espin (1:5 actin)	10 ± 3	96 ± 20	7.7 ± 0.5
Fascin (1:1 actin)^c^	18 ± 2.3	113 ± 18	8.5 ± 0.2
Espin (1:1 actin)	10 ± 2.3	69.5 ± 18.5	7 ± 1.8
Espin (2:1 actin)	16 ± 1.4	97 ± 12.3	7.2 ± 0.3

a Values resulting from analysis of five or more bundles.

b Values resulting from analysis of three bundles.

c Values similar to those in previous studies (Ishikawa et al., 2003; Jansen et al., 2011).

Espin also generated parallel actin bundles (Fig. 1 F). However, the bundles formed either at 1:1 or 2:1 espin:actin molar ratios were thinner than fascin–actin bundles, with F-actin more closely spaced in them (∼ 7 nm; Table 1). The closer spacing may be due to espin’s smaller size than fascin (29 versus 55 kDa). The espin–actin bundle formation at higher espin:actin molar ratios is consistent with previous work, in which 4:1 ratio of espin (espin-4) to actin was used (Claessens et al., 2008). However, wider bundles at a lower ratio of espin to actin (1:7 molar ratio) have also been seen (Bartles et al., 1998). These differences may be due to experimental conditions—specifically, the incubation temperature of actin with espin (37°C in Bartles et al. [1998] versus room temperature [RT] in our experiments). Taken together, these results show robust F-actin bundling by both fascin and espin, but indicate that fascin is more potent in that than espin.

### Fascin and espin do not compete for binding to F-actin

To define better fascin and espin’s F-actin bundling, we examined their localization along F-actin. Previously, another espin isoform (espin-2B) was shown to be associated with fascin domains in actin bundles (Winkelman et al., 2016). Therefore, we used three-color total internal reflection fluorescence microscopy (TIRFM) assays to image simultaneously fascin, espin, and F-actin. Notably, we found that fascin and espin co-localize on actin bundles (Fig. S1 D). In light of this co-localization along F-actin, we tested whether fascin and espin compete for F-actin binding. In sedimentation assays, fascin’s presence reduced only marginally the bound espin in actin bundles (Fig. S1 E). Similarly, only a marginal reduction in fascin binding to bundled F-actin was observed in espin’s presence (Fig. S1 F). These results reveal little or no competition between fascin and espin for their binding to bundled F-actin. Thus, as both these two proteins are associated with common F-actin structures, such as filopodia, microvilli, and stereocilia (Rajan et al., 2023a), we conclude that although fascin and espin colocalize along filaments, they likely bind to different regions in them (as they have different ABS sequences, Fig. 1 A). Based on our data (Fig. 1 C) and earlier studies (Aramaki et al., 2016; Gong et al., 2025; Jansen et al., 2011; Song et al., 2025), under saturating conditions, one fascin molecule binds every 13 actin monomers within bundles and most likely leaves enough space for espin binding between two bound fascins.

### Fascin and espin synergize to form actin bundles

**Figure 2.**
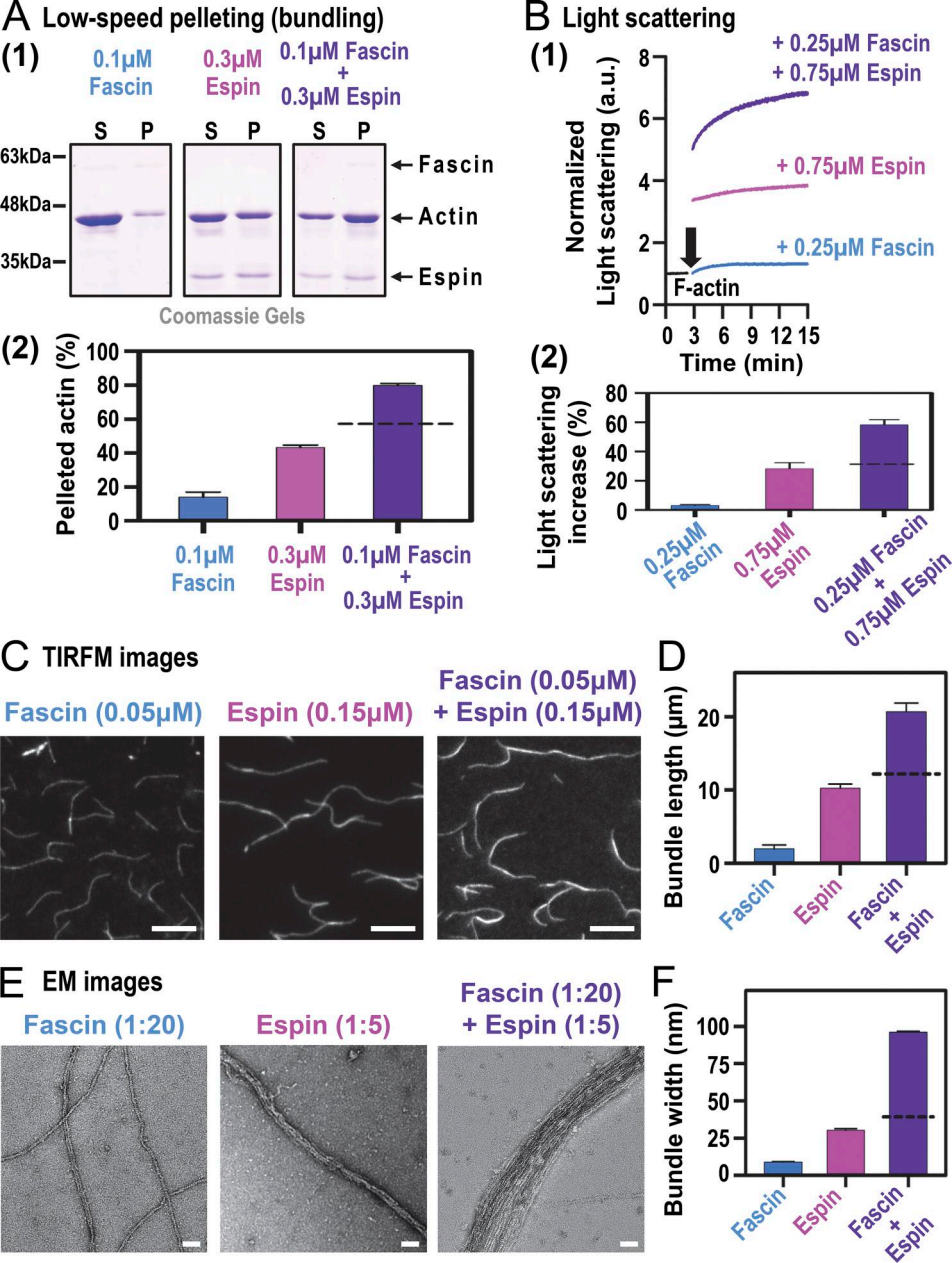
**Fascin and espin synergize in actin bundles formation. (A)** Low-speed sedimentation assays show that together fascin and espin bundle more actin filaments than would be expected from the sum of their individual effects. A representative SDS polyacrylamide gel shows supernatant (S) and pellet (P) fractions of actin in the low-speed sedimentation experiments (1). The corresponding percentage of actin in the pellets (mean values ± SEM) is shown in 2. Dashed line indicates the sum of fascin (blue) and espin (pink) values. [F-actin] = 2 μM; [Fascin] and/or [Espin] are indicated in the figure. *n* = 5 independent experiments/conditions. **(B)** Individual or combined effects of the bundling proteins on actin, as followed by light-scattering changes at λ = 325 nm. Black trace corresponds to polymerized unbundled actin (F-actin), and the arrow indicates the addition of bundling protein(s). Results of a representative experiment are shown in 1, and the corresponding percentage increase of light scattering (mean ± SEM) from *n* = 3 independent experiments/conditions is shown in 2. Dashed line indicates the sum of fascin (blue) and espin (pink) values. [F-actin] = 5 μM; [Fascin] = 0.25 μM; [Espin] = 0.75 μM; *n* = 3 independent experiments/conditions. **(C)** TIRFM images of actin bundles taken 5 min after addition of bundling proteins to the on-slide polymerized actin filaments. [Actin] = 1 µM, 20% Alexa488-labeled; [Fascin] = 0.05 μM and/or [Espin] = 0.2 μM. Scale bars: 10 μm. **(D)** Average bundles length (±SEM) determined from TIRFM images taken in three independent experiments. *n* = 27, 55, and 71 bundles analyzed for fascin only, espin only, and fascin + espin conditions, respectively. Dashed line indicates the expected sum of fascin (blue) and espin (pink) values. **(E)** EM images of actin bundles in the presence of fascin or espin or both. In the presence of both fascin and espin, much thicker bundles are formed than would be expected from the sum of their concentrations (shown as dashed line in F). Scale bars: 50 nm. **(F)** Average bundles width (±SEM) determined from EM images taken in three independent experiments. *n* = 3, 7, and 8 bundles analyzed for fascin only, espin only, and fascin + espin conditions, respectively. [F-actin] = 1 μM, [Fascin] = 0.05 μM, and/or [Espin] = 0.2 μM. Dashed line indicates the expected sum of fascin (blue) and espin (pink) values. Source data are available for this figure: SourceData F2.

Our observations support previous results from multiple labs that fascin and espin are robust F-actin–bundling proteins (Rajan et al., 2023a). Previous results with purified proteins have also revealed that together fascin and espin strongly increase F-actin bundles size (Claessens et al., 2008). We therefore further examined their combined bundling effects. Interestingly, our results—using sedimentation and light-scattering assays—indicated that when fascin and espin were added together, they were more effective at bundling F-actin than the sum of their individual effects—i.e., they showed a synergistic effect in F-actin bundling (Fig. 2, A and B). Thus, we investigated the potential synergistic effect of these two bundlers.

**Figure S2.**
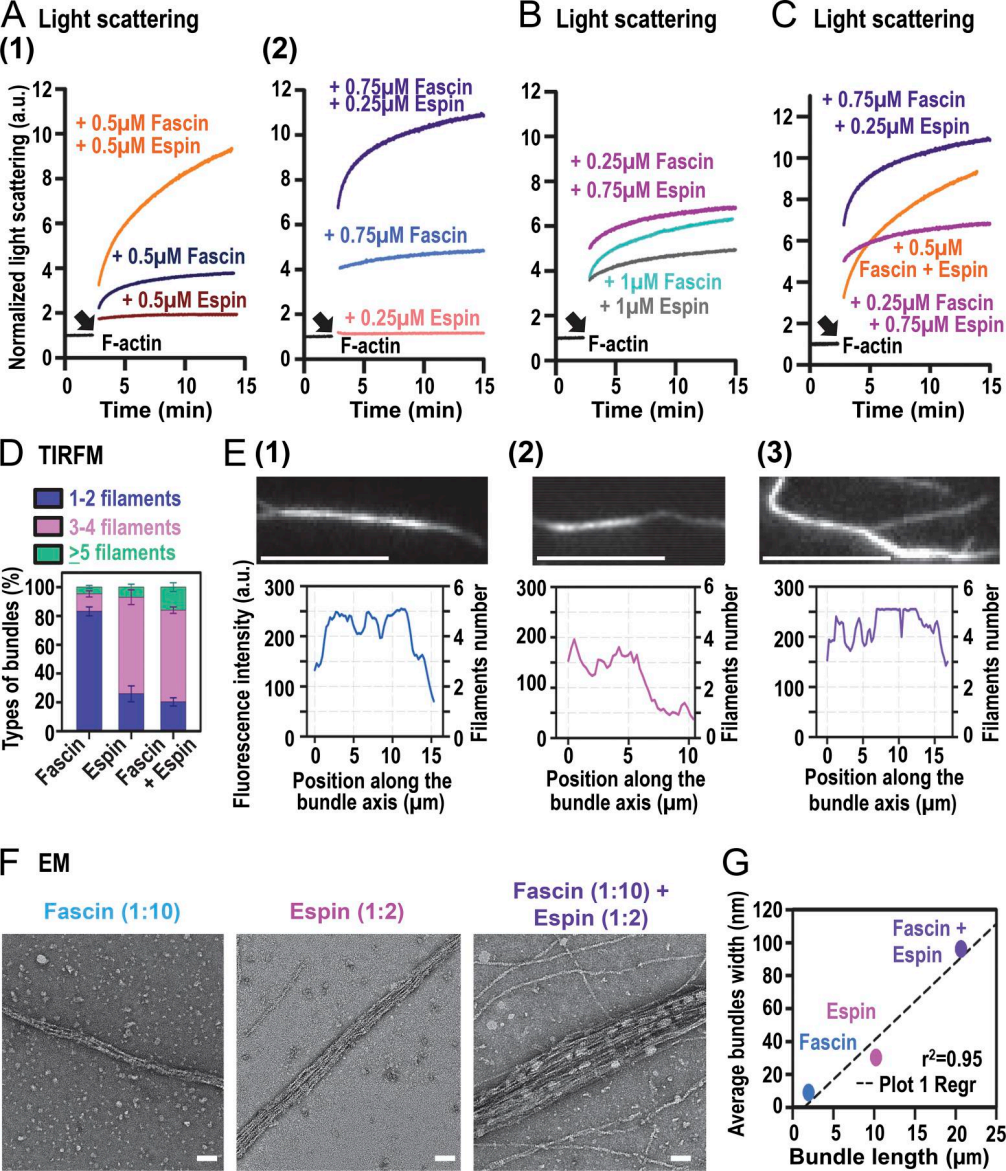
**Further analysis of the effect of different combinations of bundling proteins on actin bundles formation and topology. (A–C)** Light-scattering assays at λ = 325 nm to monitor actin bundling. The arrows indicate the addition of bundling protein(s) to F-actin (short black trace). **(A)** Individual and combined effects of fascin and espin, supporting their synergy in F-actin bundling (since less actin was bundled based on the sum of the amounts when they were individually present compared with being present together). (1) and (2) show different concentrations of fascin and espin. **(B)** Comparison of light scattering at the same total concentrations (1 μM) of fascin or espin added to F-actin *versus* fascin and espin added together to it. The results show a greater increase in light-scattering intensity when both fascin and espin are present. **(C)** Results showing that at the same total molar concentrations there is a greater increase in light-scattering intensity with addition of increasing amounts of fascin than with increasing concentrations of espin. These results indicate a greater bundling contribution of fascin than of espin to their synergistic effect. [F-actin] = 5 μM; [Fascin] and [Espin] are indicated. *n* = three independent experiments/conditions for A–C. Note also that in addition to the low-speed sedimentation assays shown in Fig. 2 A, additional low-speed sedimentation assays were also carried out. Although these assays are not as sensitive/analysis of this data is less accurate compared with the light-scattering, TIRFM, or EM assays we performed (Fig. 2, B–F; and Fig. S2, A–G), their results agree in general terms with them. **(D)** Different types of bundles (consisting of two filaments, three to five filaments, or at least five filaments) are observed using TIRFM (e.g., Fig. 2 C). The plot shows the mean percentage (±SEM) of bundles types. *n* = 62, 112, and 715 bundles analyzed for 0.05 µM fascin, 0.15 µM espin, and 0.05 µM fascin + 0.15 µM espin, respectively, from three to five separate experiments/conditions. **(E)** Fluorescence intensity profiles along the bundles indicate that the final bundles consist of up to five filaments. TIRFM images of representative actin bundles are shown at the top of the plots for (1) fascin, (2) espin, and (3) fascin–espin co-bundled F-actin. [F-actin] = 1 μM; [Fascin] = 0.2 μM, [Espin] = 0.2 μM, and [Fascin] = 0.05 μM and [Espin] = 0.15 μM for fascin and espin co-bundled F-actin, *n* = three independent experiments/conditions. Scale bars: 10 μm. **(F)** EM images of actin bundles in the presence of fascin or espin or both. Much thicker bundles are formed in the presence of both fascin and espin. [F-actin] = 1 μM, [Fascin] = 0.1 μM, and/or [Espin] = 0.5 μM. *n* = three independent experiments/conditions. Scale bars: 50 nm. **(G)** Correlation between bundle’s length and width. Pearson correlation coefficient, r = 0.95 determined using SigmaPlot 14 software. This analysis, although imperfect because it necessitated performing it on both TIRF and EM results, supports the synergistic increase in length and width of bundles that we observe in the presence of fascin and espin. Each dot represents average data (mean values ± SEM) from three independent experiments. For TIRF results, *n* = 27, 43, and 65 bundles were analyzed for fascin only, espin only, and fascin + espin conditions, respectively. For EM results, *n* = 3, 7, and 8 bundles were analyzed for fascin only, espin only, and fascin + espin conditions, respectively. [Fascin] = 0.05 μM, [Espin] = 0.2 μM, and [Fascin] = 0.05 μM and [Espin] = 0.2 μM for fascin and espin co-bundled F-actin.

To simplify this task, we chose protein concentrations at which each bundling protein alone yielded limited bundles. Yet, we found that when present together these two proteins strongly increased the amount of pelleted (bundled) actin to ∼80%, i.e., ∼23% over the sum of their individual pelleting effects (14% [fascin] + 43% [espin] = 57%) (Fig. 2 A). We also examined these effects using light-scattering assays, which we found to be more sensitive for monitoring fascin and espin’s synergy. Similarly, we found that together fascin and espin increased light scattering beyond the sum of their individual effects (Fig. 2 B and Fig. S2 A). In each case, at the same total concentrations of fascin or espin, the light-scattering increase was greater when both proteins were present (Fig. S2 B, blue line [only fascin] and gray line [only espin], and compare it with the orange and purple lines in Fig. S2 A (1 and 2), and the fuchsia line in Fig. S2 B), showing their synergistic effects in actin bundling.

Notably, the light-scattering increase was fascin concentration dependent (Fig. S2 C), indicating its greater than espin’s bundling contribution to their synergistic effect. These synergistic effects are also supported by additional assays described below. Thus, fascin and espin synergize to form actin bundles since together their ability to form F-actin bundles is greater than the sum of their individual effects.

### Fascin and espin synergize to increase actin bundles length and width

To gain more insight into how fascin and espin regulate actin bundles properties, we used TIRFM to compare their individual and combined effects on actin bundles. The images shown in Fig. 2 C are representative of actin bundles after treatment of on-slide polymerized Alexa488-labeled actin with bundling protein(s). First, we measured actin bundles length under these conditions. With fascin (0.05 µM), mainly F-actin was seen, with occasional short bundled actin regions (Fig. 2 C). With espin (0.15 µM), more bundles were seen, with an average length of ∼10 μm (Fig. 2 C). However, with both fascin (0.05 µM) and espin (0.15 µM), the bundles were approximately twofold longer than the combined length of bundles formed with fascin and espin added alone (Fig. 2 D). These results further support fascin and espin’s synergistic effect on F-actin bundling.

Next, we measured the thickness of fascin, espin, and fascin–espin–actin bundles. Using TIRFM, we observed that in the presence of both fascin and espin, versus either fascin or espin alone, there was a trend toward increased bundling and thicker bundles (at least five filaments/bundles) (Fig. S2 D). However, due to fluorescence intensity saturation (after five filaments; Fig. S2 E), only limited filament numbers in the bundles could be measured using TIRFM. Thus, we used EM to measure the bundles’ width.

Consistent with our TIRFM analysis, at low fascin (only) concentrations, mostly single filaments were observed, with just a few bundles of two filaments (Fig. 2, E and F; and Table 1). With espin only, thin bundles (∼30 nm wide) consisting of four to five filaments were observed (Fig. 2, E and F; and Table 1). In the presence of both fascin and espin, the bundles were approximately two to three times wider (∼100 nm) than those of either fascin or espin bundles, with >10 filaments (Fig. 2, E and F; and Table 1). Further increasing fascin and espin levels induced thicker bundles (Fig. S2 F). The interfilament distance in these fascin + espin bundles was ∼7.7 nm, a value intermediate of that observed for fascin or espin-only bundles (Table 1). Our analysis of both TIRFM and EM results are also consistent with there being a correlation between the fascin + espin synergistic increase in length and width (r^2^ = 0.95; Fig. S2 G). Thus, fascin and espin synergize to generate thicker and longer actin bundles compared with the sum of either bundler alone. This synergistic interaction between fascin and espin provides mechanistic insights into previous observations (mentioned above) that the presence of both fascin and espin strongly increases F-actin bundles size (Claessens et al., 2008). This synergistic interaction we have identified also provides mechanistic insights into cellular results revealing that fascin and espin together generate thicker F-actin bundles (Krishnan et al., 2021; Tilney et al., 1995) and longer filopodia (Chou et al., 2011) than either protein on its own.

### Mical extensively dismantles espin-bundled F-actin—as well as complex F-actin bundles composed of both fascin and espin

**Figure 3.**
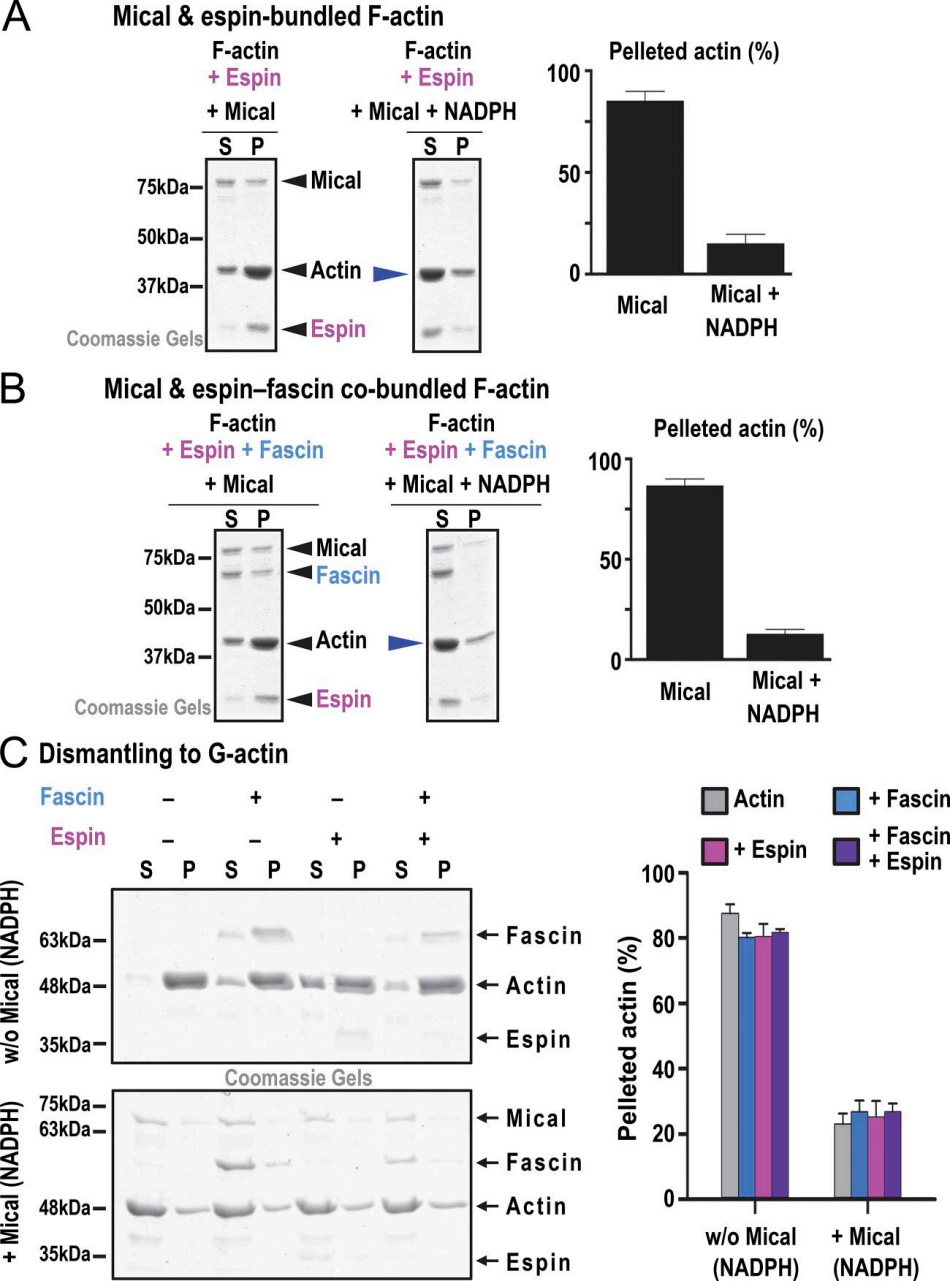
**Mical robustly disassembles complex different protein-bundled F-actin structures. (A)** Mical/NADPH disassembles espin-bundled F-actin. In low-speed pelleting assays, a majority of espin-bundled F-actin is present in the pellets (left). Addition of Mical and NADPH robustly reduces the amount of F-actin in the pellets (blue arrowhead, graph). [F-actin] = 2.5 μM; [Espin] = 1.25 μM; [Mical] = 0.6 μM; [NADPH] = 100 μM. Mean values ± SEM. *n* = 2 independent experiments/conditions. **(B)** Mical/NADPH disassembles fascin- and espin-bundled F-actin. In low-speed pelleting assays, a majority of fascin + espin-bundled F-actin is present in the pellets (left), but the addition of Mical and NADPH decreases strongly the pelleted F-actin (blue arrowhead, graph). [F-actin] = 2.5 μM; [Fascin] = 1.25 μM; [Espin] = 1.25 μM; [Mical] = 0.6 μM; [NADPH] = 100 μM. Mean values ± SEM. *n* = 2 independent experiments/conditions. **(C)** In high-speed pelleting assays, in all cases a majority of actin is present in the pellets (F-actin), but the addition of Mical and NADPH decreases the pelleted F-actin (plot on the right) and disassembles it down to G-actin (gel on the left). [F-actin] = 2.5 μM; [Fascin] = 1.0 μM for fascin only bundles; [Espin] = 1.0 μM for espin only bundles; [Fascin] = 0.5 μM and [Espin] = 0.5 μM for fascin–espin co-bundles; [Mical] = 0.6 μM; [NADPH] = 100 μM. Mean values ± SEM. *n* = 3 independent experiments/conditions. Source data are available for this figure: SourceData F3.

Our previous results revealed that Mical rapidly and extensively dismantles fascin-bundled F-actin (Hung et al., 2010; Rajan et al., 2023c); hence, we examined its effect on espin-bundled F-actin. Notably, using low-speed sedimentation, we found that Mical markedly reduced the amount of espin-bundled F-actin (Fig. 3 A). These results reveal that Mical robustly disassembles espin-bundled F-actin. Thus, we looked at the effects of Mical on fascin + espin co-bundled F-actin. In particular, the actin bundles in cells contain multiple bundling proteins and undergo tightly regulated spatiotemporal disassembly and turnover to support cellular responses (cytoskeletal remodeling) (Blanchoin et al., 2014; Lappalainen et al., 2022). However, to the best of our knowledge, no proteins have been identified that destabilize bundles composed of multiple bundling proteins. Notably, using low-speed sedimentation, we found that Mical markedly reduced the amount of fascin–espin co-bundled F-actin (Fig. 3 B). We also found using high-speed disassembly assays that Mical not only disassembles these fascin–espin co-bundled filaments to unbundled filaments (as revealed by low-speed sedimentation; Fig. 3, A and B) but also to G-actin (Fig. 3 C). Thus, Mical robustly disassembles complex F-actin bundles—i.e., those simultaneously bundled with more than one type of bundling protein—including down to the G-actin form.

### Fascin and espin synergize to increase F-actin’s protection against disassembly by Mical

**Figure 4.**
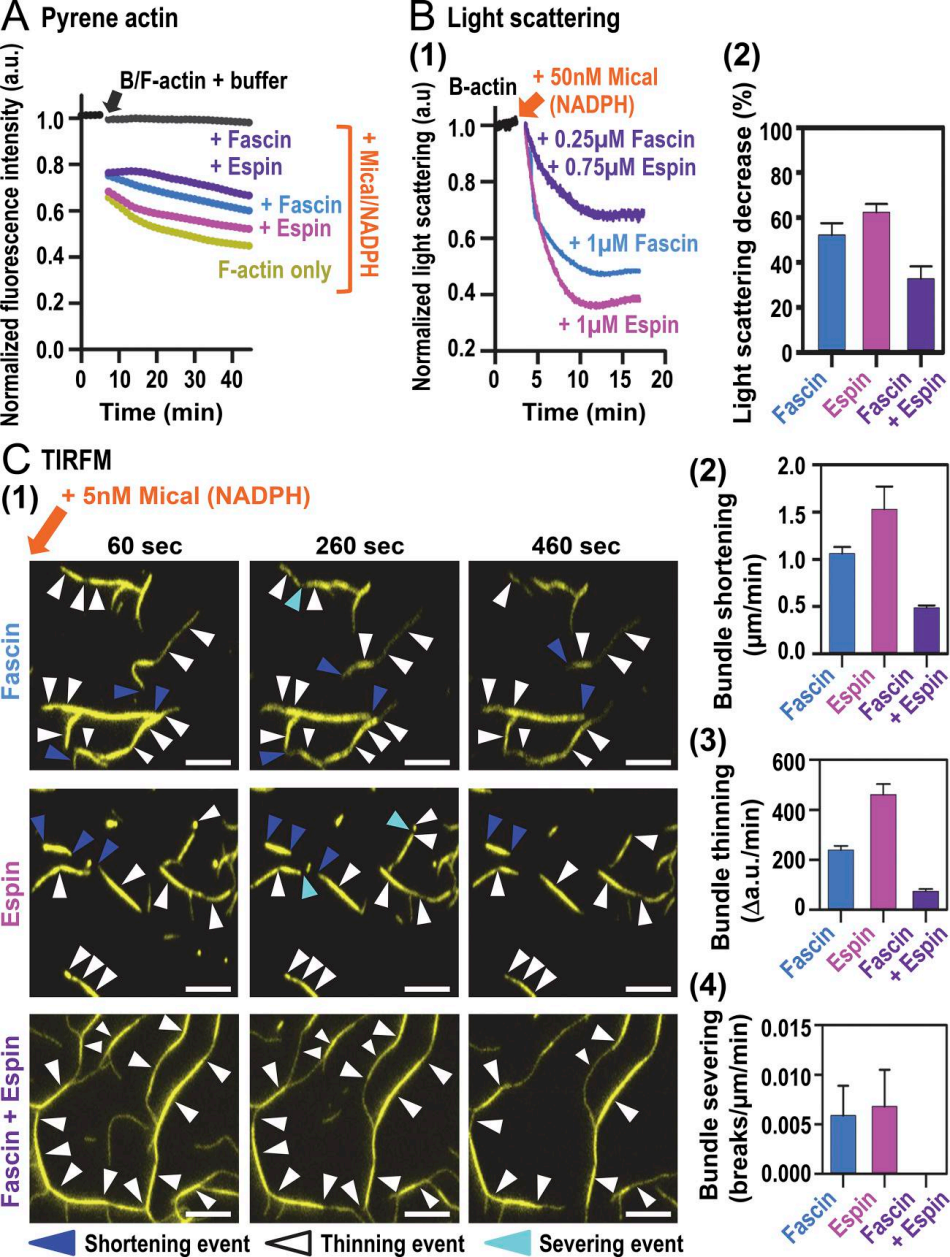
**Fascin and espin synergize to dampen Mical’s disassembly of F-actin. (A)** Comparison of Mical’s effects on unbundled, fascin-bundled, espin-bundled, and fascin–espin co-bundled filaments. Representative pyrene-actin assay is shown. The black trace corresponds to F-actin or B-actin (bundled actin), and the arrows indicate the addition of buffer or Mical/NADPH. [Actin] = 5 μM, [Fascin] = 1 μM for fascin only bundles, [Espin] = 1 μM for espin only bundles, [Fascin] = 0.5 μM and [Espin] = 0.5 μM for fascin–espin co-bundles; [Mical] = 0.2 μM and [NADPH] = 100 μM. One representative experiment of *n* = 3 independent experiments/conditions. **(B and C)** Mical/NADPH disassembles fascin–espin co-bundled F-actin, but less efficiently than F-actin bundled with either fascin or espin alone. **(B)** Light-scattering experiments (at λ = 325 nm): a black trace corresponds to bundled F-actin (B-actin), and the arrow indicates Mical/NADPH addition. Results of a representative experiment from *n* = 3 independent experiments/conditions are shown in 1, and the corresponding percentage decrease in light scattering (mean ± SEM) is shown in 2. [F-actin] = 5 μM; [Fascin] and/or [Espin] = as indicated; [Mical] = 50 nM, and [NADPH] = 200 µM. **(C)** TIRFM assays. (1) Representative time-lapse TIRFM images of fascin-bundled, espin-bundled, and fascin–espin co-bundled F-actin disassembly in the presence of Mical. The arrow indicates Mical/NADPH addition. There was a delay of ∼60 s between the addition of Mical/NADPH and the start of video acquisition. This time is included in the figure. Arrowheads of the different colors designate examples of the noted events (shortening, thinning, and severing). Scale bars: 10 μm. (2–4) Bundle shortening (2), thinning (3), and severing (4) rates (mean values ± SEM) after Mical/NADPH addition (obtained from TIRFM videos analysis). Bundles shortening data were averaged from *n* = 11, 11, and 21 bundles for fascin only, espin only, and fascin + espin conditions, respectively (from three independent experiments/conditions). Bundles thinning rates were determined from *n* = 14, 25, and 24 bundles for fascin only, espin only, and fascin + espin conditions, respectively. [Actin] = 1 µM, 20% Alexa488-labeled, [Mical] = 5 nM, [NADPH] = 100 µM, [Fascin] = 0.2 μM for fascin–actin bundles, [Espin] = 0.2 μM for espin–actin bundles, [Fascin] = 0.1 μM and [Espin] = 0.1 μM for their combined bundles.

We next compared the impact of Mical on unbundled, fascin-bundled, espin-bundled, and fascin–espin co-bundled F-actin. Consistent with our previous results, we found using pyrene actin assays that fascin-bundled F-actin was more resistant to Mical’s F-actin disassembly than unbundled F-actin (Fig. 4 A [Rajan et al., 2023c]). Notably, we also saw a similar protective effect of espin on F-actin: that espin-bundled F-actin was more resistant to Mical’s disassembly than unbundled F-actin (Fig. 4 A). Furthermore, when both fascin and espin were present in bundles, we found that they were even more resistant to Mical’s disassembly (Fig. 4 A). These results indicate that the joint presence of fascin and espin serves to increase F-actin’s protection against Mical-mediated disassembly.

**Figure S3.**
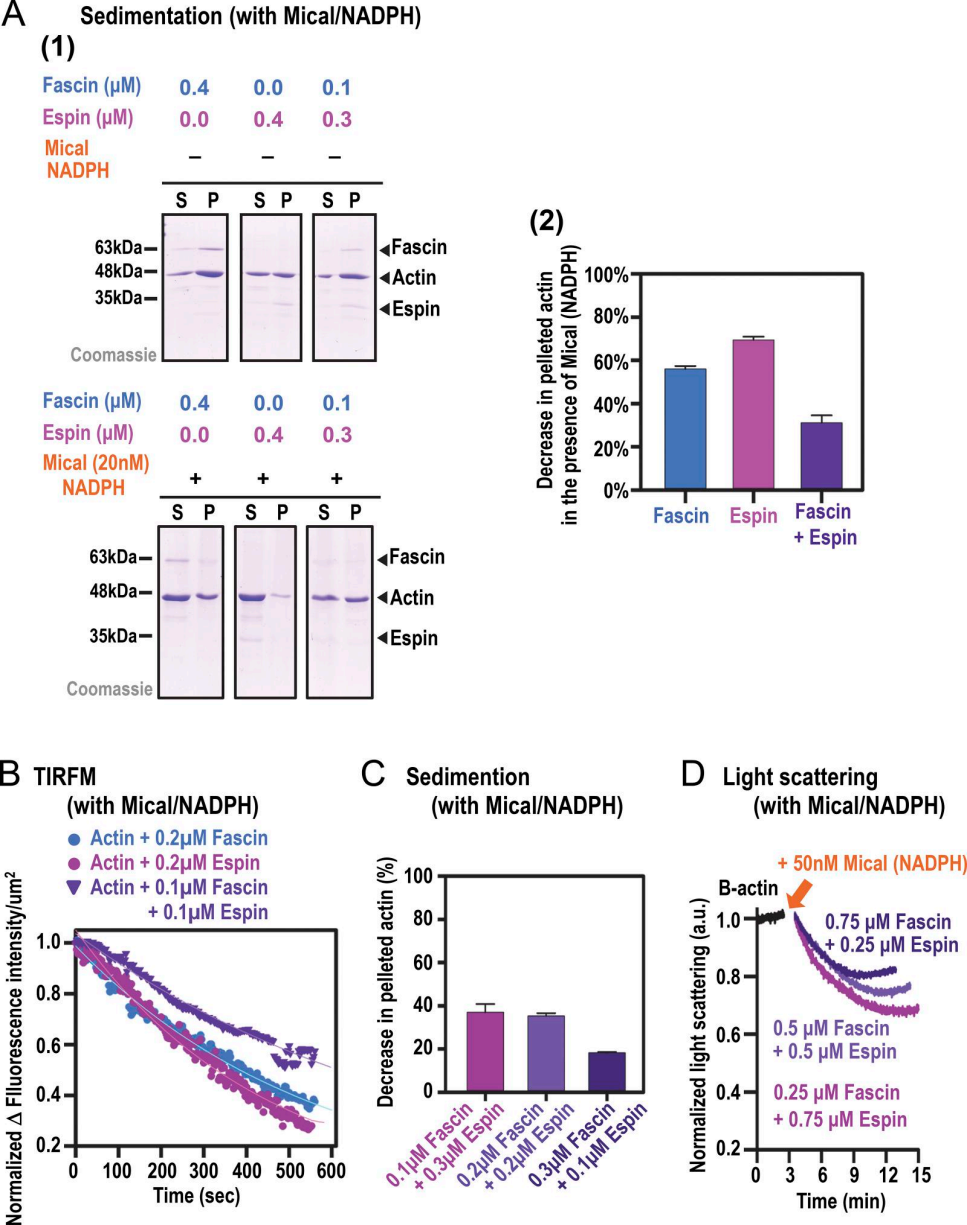
**Further analysis of the Mical’s disassembly effects on different combinations of bundling proteins in actin bundles. (A)** Low-speed pelleting assays using low Mical concentrations reveal that Mical/NADPH robustly disassembles fascin–actin bundles and espin–actin bundles. However, their co-bundles resist this disassembly more effectively. (1) SDS-polyacrylamide gels of supernatant (S) and pellet (P) fractions of different actin–bundling proteins samples in the absence (top) or presence (bottom) of Mical/NADPH after their low-speed sedimentation. (2) The corresponding decrease in the percentage of actin found in the pellets (mean values ± SEM). [F-actin] = 2 μM; [Fascin] and/or [Espin] = as indicated in the figure; [Mical] = 20 nM, and [NADPH] = 200 µM; *n* = 3 independent experiments/conditions. **(B)** Average fluorescence intensities/µm^2^ in TIRFM movies reveal that Mical/NADPH induces a ∼1.5- and 2.1-fold slower fluorescence signal decay in fascin–espin–actin bundles (purple) compared with that seen with equimolar concentrations of fascin (blue) or espin (pink) bundles, respectively. Symbols represent the experimental data and the solid lines are the exponential fits to determine the rates values. Each decay curve is the averaged scan of *n* = 24, 14, and 25 bundles analyzed for fascin + espin, fascin alone, and espin alone conditions, respectively (taken from three separate experiments). [Actin] = 1 µM, 20% Alexa488-labeled, [Fascin] and/or [Espin] are as indicated in the figure, [Mical] = 5 nM, and [NADPH] = 100 µM. **(C and D)** Low-speed pelleting (C) and light-scattering (D) assays using fascin–espin–actin bundles with different concentration ratios of fascin and espin. The efficiency of Mical/NADPH-mediated disassembly decreases with increasing fascin concentrations used for these bundles. **(C)** Mean values ±SEM, [F-actin] = 2 μM; bundling proteins as indicated, [Mical] = 20 nM, and [NADPH] = 200 µM; *n* = 3 independent experiments/conditions. **(D)** [F-actin] = 5 μM; bundling proteins as indicated, [Mical] = 50 nM, and [NADPH] = 200 µM; *n* = 3 independent experiments/conditions. Source data are available for this figure: SourceData FS3.

To better quantify and compare Mical’s effect on these different types of bundled filaments, we reduced its concentration. Using sedimentation assays, we found that these lower Mical concentrations still decreased the amount of pelleted (bundled) actin in all three bundling proteins conditions (Fig. S3 A (1 and 2)). Yet, although we used similar bundling protein concentrations in all conditions (0.4 μM), the percentage decrease in the pelleted (bundled) actin was smaller for bundles containing both fascin and espin than with either fascin or espin alone (Fig. S3 A (2)). We also observed a similar trend in our light-scattering assays (Fig. 4 B). To examine better these effects at a molecular level, we used time-lapsed TIRFM assays. We found that Mical disassembled F-actin bundled with fascin, espin, and fascin + espin (Fig. 4 C and Video 1). However, the joint presence of fascin and espin notably slowed Mical’s F-actin disassembly—these co-bundles persisted longer than those formed in the presence of equal molar concentrations of either bundler alone (Fig. 4 C and Video 1).

**Video 1.** **Mical/NADPH-mediated disassembly of fascin-bundled, espin-bundled, and fascin + espin co-bundled actin filaments. Related to Fig. 4 C.** 5 nM Mical (+100 µM NADPH) was added to 1 µM actin (20% Alexa488) bundled with 0.2 µM fascin or 0.2 µM espin or 0.1 µM fascin + 0.1 µM espin. By comparing the videos, it is clear that fascin-only and espin-only bundles are shorter than those formed in the presence of both fascin and espin. Upon Mical/NADPH addition, disassembly of all bundles types takes place. However, our quantitative analysis reveals that it is more severe for bundles formed in the presence of fascin or espin alone versus both bundlers present together (see also Fig. 4 C (2–4)). White, blue, and cyan arrowheads indicate examples of thinning, shortening, and severing of bundles, respectively. Movie speed: 25 frames/s. Scale bars: 10 μm.

Mical has been found to disassemble actin bundles by shortening and thinning them (Rajan et al., 2023c). Moreover, the thinning sites subsequently become bundles severing sites (Fig. 4 C, cyan arrows). Thus, we compared the shortening, thinning, and severing rates of all three bundles types (Fig. 4 C (2–4)). We found that when fascin and espin were present together, Mical’s bundles shortening was slowed (on average) by 2.2-fold and 3.1-fold compared with its effects on similar molar concentrations of fascin- or espin-bundled F-actin (0.48 ± 0.02 µm/min versus 1.06 ± 0.07 µm/min and 1.53 ± 0.24 µm/min, respectively) (Fig. 4 C (2)). So too, for the first 120 s of data acquisition, the thinning of fascin–espin-bundled F-actin by Mical occurred at ∼3.2- and 6.2-fold slower rate as compared with that of F-actin bundled with fascin or espin alone (Fig. 4 C (3)). Moreover, the overall (for 10 min) thinning rate was ∼1.5- and 2.1-fold slower when both bundlers were present than when they were individually present (Fig. S3 B). These observations suggest that initially, fascin–espin co-bundles robustly resist the Mical-mediated thinning, but eventually begin to disassemble. No severing was observed for all three bundles types for the initial 60 s. Severing was observed subsequently in the fascin-only and espin-only bundles. However, for fascin–espin co-bundled F-actin, no severing was observed even after 400 s (Fig. 4 C (4)), despite the observed bundles thinning. We also noted that similar to the greater contribution of fascin to the fascin–espin synergistic bundling effect (Fig. S2 C), both low-speed sedimentation and light-scattering assays revealed that it also provided a greater contribution to fascin–espin co-bundles resistance to their Mical-mediated disassembly (Fig. S3, C and D). Thus, while Mical disassembles actin bundled with both fascin and espin, these proteins synergize to help protect F-actin from disassembly.

### Mical and cofilin synergize to disassemble fascin–espin co-bundled F-actin—but fascin and espin synergize to help protect it against this disassembly

**Figure 5.**
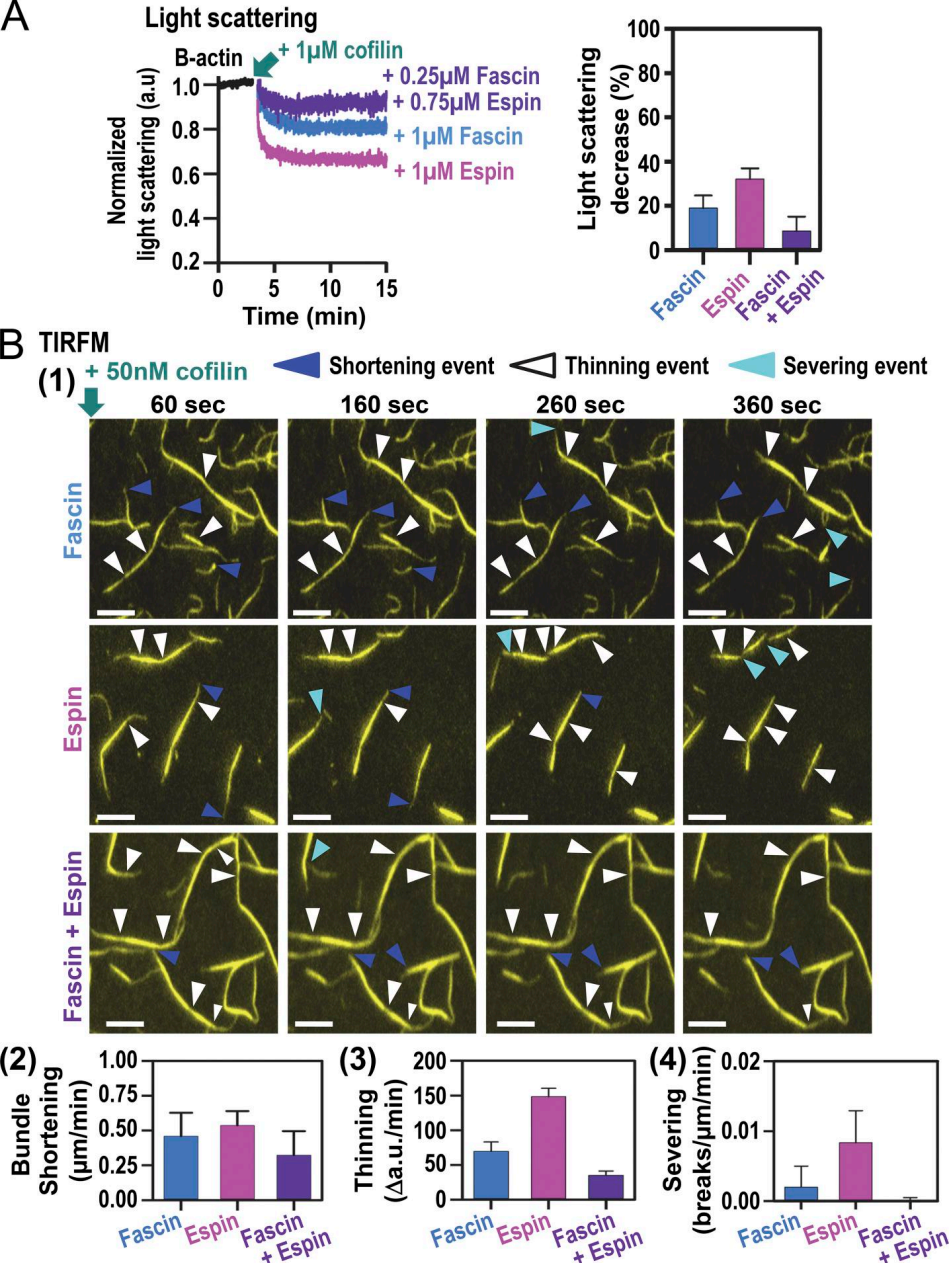
**Cofilin affects espin, fascin, and espin–fascin co-bundled F-actin. (A and B)** Light-scattering (A) and TIRFM (B) assays reveal that cofilin disassembles espin-bundled filaments and has a stronger effect on them than on fascin-bundled filaments. These experiments also reveal that cofilin is even less effective against F-actin bundles containing both fascin and espin. **(A)** Results of a representative light-scattering experiment (at λ = 325 nm) are shown in A (left), and the corresponding percentage decrease in light scattering (mean values ± SEM) from *n* = 3 independent experiments/conditions is shown in A (right). Black trace corresponds to F-actin bundles (B-actin), and the arrow indicates cofilin addition. Note the decrease in light scattering at each condition and a limited decrease when fascin and espin are added together. [F-actin] = 5 μM; [Fascin] and/or [Espin] = as indicated in the figure; [cofilin] = 1 μM. **(B)** Similar results were seen in our TIRFM assays. (1) Representative time-lapse TIRFM images of cofilin-mediated disassembly of fascin-bundled, espin-bundled, and fascin–espin co-bundled F-actin. The arrow indicates cofilin addition. There was a delay of ∼60 s between the addition of cofilin and the start of video acquisition. This time is included in the figure. Arrowheads of the different colors designate examples of the noted events (shortening, thinning, and severing). Scale bars: 10 μm. Rates (mean values ± SEM) of cofilin-mediated bundles shortening (2), thinning (3), and severing (4) determined from TIRFM assays. [Actin] = 1 µM, 20% Alexa488-labeled, [cofilin] = 50 nM, [Fascin] = 0.2 μM for fascin–actin bundles, [Espin] = 0.2 μM for espin–actin bundles, and [Fascin] = 0.1 μM + [Espin] = 0.1 μM for co-bundles. The shown values were obtained from *n* > 10 bundles/conditions in three independent experiments/conditions.

To further investigate the mechanisms underlying fascin–espin co-bundled F-actin disassembly, we looked at cofilin’s effects on them. Cofilin disassembles fascin-bundled F-actin, but not as well as Mical (Rajan et al., 2023c; and also see Breitsprecher et al., 2011; Chikireddy et al., 2024; Elkhatib et al., 2014; Schmoller et al., 2011). We therefore examined cofilin’s effects on espin-bundled F-actin. Using light-scattering and TIRFM assays, we found that cofilin disassembled espin-bundled F-actin, having a stronger effect on them than on fascin-bundled F-actin (Fig. 5, A and B; and Video 2). Furthermore, as we saw with Mical, cofilin caused less disassembly of fascin–espin co-bundled filaments than those with equimolar concentrations of fascin or espin (Fig. 5, A and B; and Video 2).

**Video 2.** **Cofilin-mediated disassembly of fascin-bundled, espin-bundled, and fascin + espin co-bundled actin filaments. Related to Fig. 5 B.** 50 nM cofilin was added to 1 µM actin (20% Alexa488) bundled with 0.2 µM fascin or 0.2 µM espin or 0.1 µM fascin + 0.1 µM espin. Upon cofilin addition, all bundles types were disassembled, but the disassembly was less than observed in the presence of Mical/NADPH. Likewise, as we saw with Mical/NADPH, there was less disassembly in the case of fascin and espin co-bundles versus those formed in the presence of fascin or espin alone (see also Fig. 5 B (2–4)). White, blue, and cyan arrowheads indicate examples of thinning, shortening, and severing of bundles, respectively. Movie speed: 25 frames/s. Scale bars: 10 μm.

**Figure 6.**
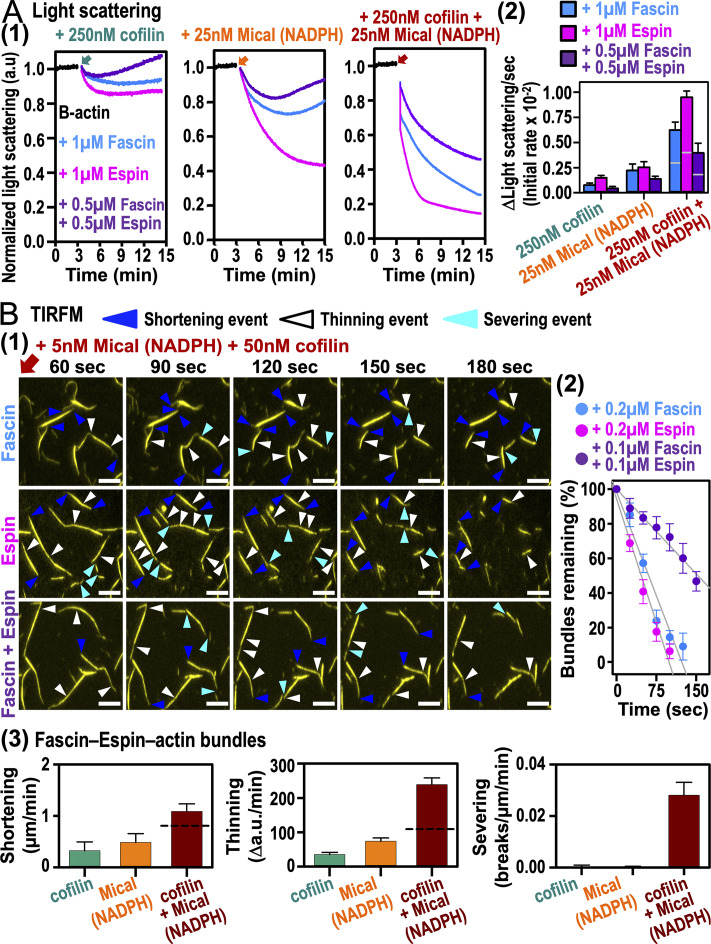
**Mical and cofilin synergize to disassemble fascin- and espin-bundled F-actin—but together fascin and espin function to increase bundles protection against this disassembly. (A)** Representative light-scattering experiments (at λ = 325 nm) are shown in 1, and the corresponding initial decrease in light scattering (mean ± SEM) from *n* = 3 independent experiments/conditions is shown in 2. Gray lines indicate the expected sum of cofilin and Mical/NADPH effects on the light scattering of the bundles. Note the more substantial change in light scattering when both cofilin and Mical are added together *versus* when they are added separately (or the sum of the two added separately). Also note that the change in light scattering is more limited under each condition when fascin and espin are both present. The black trace corresponds to B-actin (bundled actin), and the arrows indicate the addition of cofilin, Mical/NADPH, or cofilin + Mical/NADPH in 1. [Actin] = 5 µM, [Mical] = 25 nM, [NADPH] = 200 µM, [cofilin] = 250 nM, [Fascin] = 1 μM for fascin only bundles, [Espin] = 1 μM for espin only bundles, and [Fascin] = 0.5 μM and [Espin] = 0.5 μM for combined bundles. *n* = 3 independent experiments/conditions. **(B)** TIRFM images of Mical and cofilin effects on actin bundles. The arrow indicates addition of Mical/NADPH and cofilin (1) and the analysis (mean ± SEM) of the percentage of remaining bundles/bundle fragments (2). There was a delay of ∼60 s between the addition of Mical/NADPH + cofilin and the start of video acquisition. This time is included in the figure. Arrowheads of the different colors designate examples of the noted events (shortening, thinning, and severing). Scale bars: 10 μm. Note that in 2, ∼2.3 and 3.2 times slower rates of bundles disappearance are seen for fascin–espin co-bundled F-actin versus F-actin bundled with equimolar concentrations of fascin or espin alone, respectively (the rates were obtained by a linear regression fit [gray lines]). (3) The plots (obtained from TIRFM videos analysis) show changes in the rates (mean ± SEM) of bundles shortening, thinning, and severing after the addition of cofilin and/or Mical/NADPH to fascin–espin–actin bundles. [Actin] = 1 µM, 20% Alexa488-labeled, [Mical] = 5 nM, [NADPH] = 100 µM, [cofilin] = 50 nM, [Fascin] = 0.2 μM for fascin only bundles, [Espin] = 0.2 μM for espin only bundles, and [Fascin] = 0.1 μM and [Espin] = 0.1 μM for the combined bundles. *n* > 15 bundles/conditions, analyzed from three independent experiments/conditions. Dashed lines indicate the expected sum of cofilin and Mical/NADPH effects when added separately.

**Figure S4.**
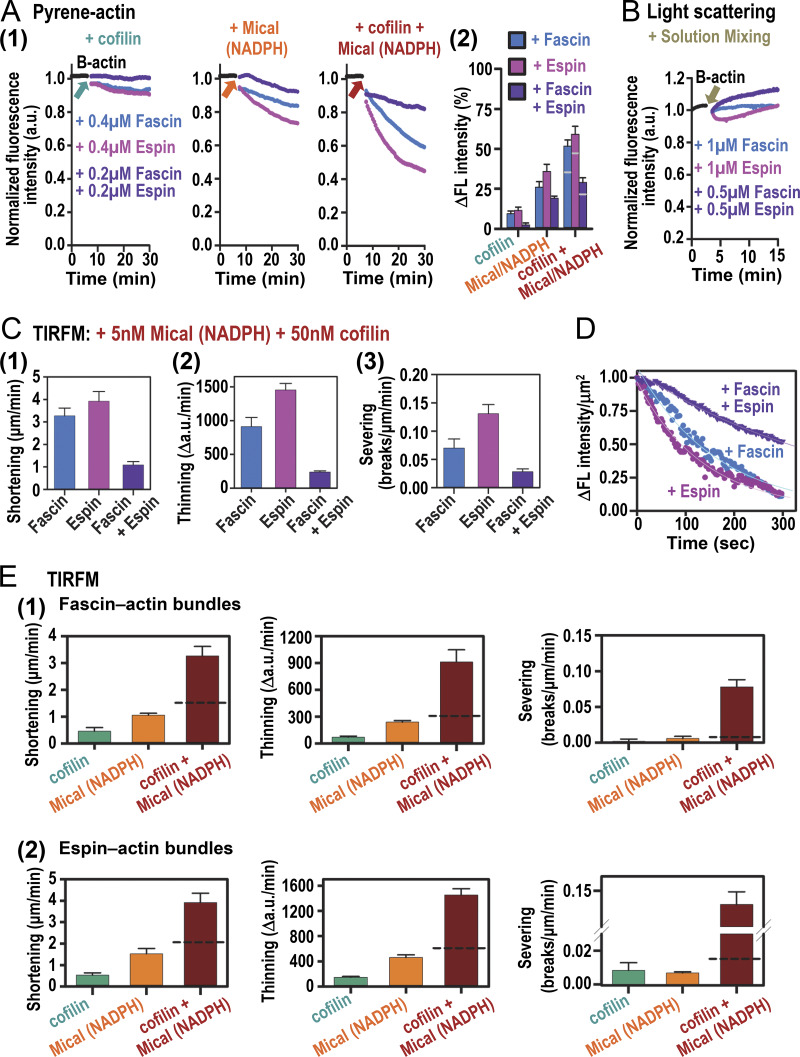
**Further analysis of Mical and cofilin synergy in disassembling fascin, espin, and fascin–espin co-bundled F-actin—and fascin and espin’s combined protection against this disassembly. (A)** Results of representative pyrene-actin fluorescence assays are shown in 1 and the corresponding percentage decrease in the fluorescence (FL) (mean ± SEM) from *n* = 3 independent experiments/conditions is shown in 2. [F-actin] = 2 μM; [Fascin] and/or [Espin] in 1 and 2 = as indicated in the figure; [cofilin] = 50 nM, [Mical] = 10 nM, [NADPH] = 100 µM. Color-coded arrows indicate the addition of cofilin, Mical (NADPH), and cofilin + Mical (NADPH). **(B)** As shown in this representative results of light-scattering experiments at λ = 325 nm (and also noted previously see Fig. 1 D of Rajan et al. [2023c]), the addition of buffer and/or solution mixing can cause initial effects on light-scattering readings (since light-scattering measurements are particle size sensitive real-time measurements). This can lead to marginal decreases in the scattering intensity. However, as shown here (and as also noted previously (see Fig. 1 D of Rajan et al. [2023c]), this transient reduction in light scattering can be reversed with time (as reflected by an increase in the light scattering), including due to reformation of actin bundles in the solution. However, in the presence of Mical/NADPH, this disassembly occurs more rapidly and is not reversed with time (e.g., see Fig. 4 B (1) of this manuscript, and also Fig. 1 D of Rajan et al. [2023c]). These characteristics make light-scattering measurements a sensitive and robust means to examine the effects of different proteins on the disassembly of bundled F-actin. In this example, the black trace corresponds to B-actin (bundled actin), and the arrow indicates mixing using a pipette. [Actin] = 5 μM, [Fascin] = 1 μM for fascin containing bundles, [Espin] = 1 μM for the bundles with espin, and [Fascin] = 0.5 μM and [Espin] = 0.5 μM for bundles with both of them. *n* = 2 independent experiments/conditions. **(C–E)** TIRFM assays. **(C)** Rates (mean ± SEM) of bundles shortening (1), thinning (2), and severing (3) as determined using TIRFM assays. **(D)** Average normalized fluorescence (FL) intensities/µm^2^ after addition of Mical/NADPH + cofilin. Symbols represent the experimental data, and the solid lines are the exponential fits to determine the rates values. *n* = 3 independent experiments/conditions. + Fascin = [0.2 μM Fascin], + Espin = [0.2 μM Espin], and + Fascin + Espin = [0.1 μM Fascin] + [0.1 μM Espin]. **(E)** The plots show changes in the rates (mean ± SEM) of bundles severing, thinning, and shortening after addition of Mical/NADPH and/or cofilin to fascin–actin bundles (1) and espin–actin bundles (2). Dashed lines indicate the expected sum of cofilin and Mical/NADPH effects when added separately. **(C–E)** [Actin] = 1 µM, 20% Alexa488-labeled, [cofilin] = 50 nM, [Mical] = 5 nM, [NADPH] = 100 µM, [Fascin] = 0.2 μM for fascin–actin bundles, [Espin] = 0.2 μM for espin–actin bundles, and [Fascin] = 0.1 μM and [Espin] = 0.1 μM for their combined actin bundles. *n* > 15 bundles/conditions from three independent experiments/conditions.

Mical and cofilin synergize to disassemble unbundled and fascin-bundled F-actin (Grintsevich et al., 2016; Grintsevich et al., 2017; Rajan et al., 2023c; Wioland et al., 2021). We therefore wondered whether Mical and cofilin also worked in a similar synergistic way to disassemble complex fascin–espin co-bundled filaments. Notably, pyrene fluorescence, light scattering, and TIRFM assays revealed that compared with the sum of their individual effects, Mical and cofilin synergized to disassemble both espin-bundled and fascin–espin co-bundled F-actin (Fig. 6, Fig. S4, and Video 3). For example, using TIRFM, we found that Mical + cofilin induced rapid and extensive bundles disassembly of fascin, espin, and fascin–espin co-bundled F-actin (Fig. 6 B (1 and 2); and Video 3). Furthermore, comparing the effects of Mical alone (Fig. 4 C), cofilin alone (Fig. 5 B), and Mical + cofilin (Fig. 6 B)—revealed that Mical + cofilin enhanced more strongly the rate and number of bundles shortening, thinning, and severing events (and note the different time scales between Fig. 6 B (1), Fig. 4 C (1), and Fig. 5 B (1)), compared with the sum of Mical and cofilin’s individual effects (Fig. 6 B (3) and Fig. S4 E).

**Video 3.** **Mical/NADPH and cofilin-mediated disassembly of fascin-bundled, espin-bundled, and fascin + espin co-bundled actin filaments. Related to Fig. 6 B.** 5 nM Mical (+100 μM NADPH) + 50 nM cofilin were added to 1 µM actin (20% Alexa488) bundled with 0.2 µM fascin or 0.2 µM espin or 0.1 µM fascin + 0.1 µM espin. A rapid and extensive disassembly of all bundles types was observed. In each case, this disassembly was more than that observed in the presence of Mical/NADPH or cofilin only (see also Fig. 6B (3) and Fig. S4 E). However, as we observed for Mical/NADPH or cofilin only, our quantitative analysis reveals that Mical + cofilin-mediated bundle disassembly is greater for bundles formed in the presence of fascin or espin alone versus those formed in the presence of both bundlers (see also Fig. S4 C, and also compare Fig. 6B (3) to Fig. S4 E (1 and 2)). Also, note that between the time Mical/NADPH + cofilin were added to the bundles and we could begin the video recording, bundles had already substantially disassembled. This was prominent for the espin–actin bundles, where only remnants of the bundles were visible. On several occasions, the disassembled fragments of bundles came together, leading to the formation of short, thick bundles, as observed at the end of the videos. White, blue, and cyan arrowheads indicate examples of thinning, shortening, and severing of bundles, respectively. Also, note the shortened time frame of this Mical/NADPH + cofilin movie (120 s) versus the 300–400 s time frame of Videos 1 and 2. Movie speed: 15 frames/s. Scale bars: 10 μm.

However, pyrene fluorescence, light-scattering, and TIRFM assays revealed also that even though Mical + cofilin strongly disassembled all three actin bundles types, their impact was reduced when both fascin and espin were present on them together (Fig. 6, A and B; Fig. S4, A–D; and Video 3). Notably, in TIRFM assays, fascin and espin alone bundled F-actin was already shorter and thinner than fascin–espin co-bundled F-actin by the time we started collecting TIRFM data, suggesting their excessive severing and disassembly (in Fig. 6 B (1) compare the 60 s for all conditions, Video 3). Additionally, a ∼2.6-fold and 3.1-fold slower bundles thinning of fascin–espin co-bundled F-actin was observed compared with that seen with equimolar concentrations of fascin or espin containing bundles, respectively (Fig. S4 D). Thus, Mical and cofilin synergize to disassemble F-actin bundles composed of two different bundlers, but when present together in these bundles, fascin and espin synergize to slow down this disassembly.

### Biochemical mechanisms underlying Mical’s disassembly of complex F-actin bundles

**Figure 7.**
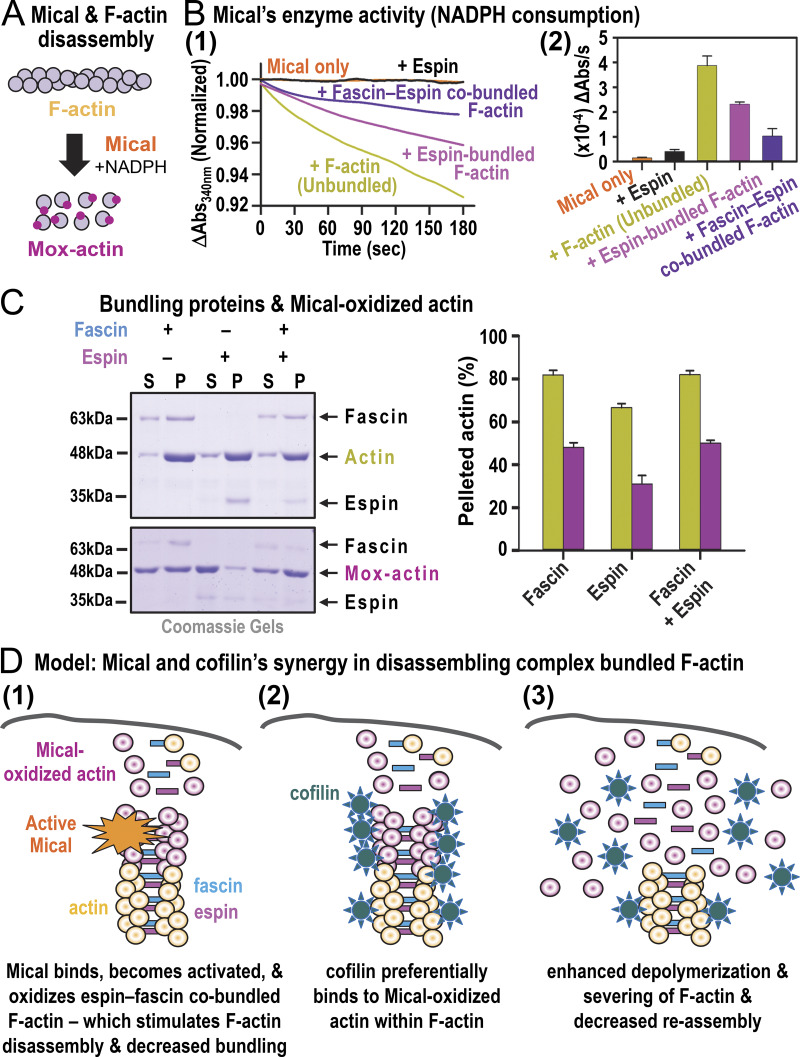
**Biochemical mechanisms underlying Mical and cofilin’s synergy in disassembling complex F-actin bundles—and fascin and espin’s protection against this disassembly. (A)** Mical, in the presence of its coenzyme NADPH, disassembles F-actin by oxidizing (fuchsia circle) it. The resulting product is Mox-actin. **(B)** Mical’s enzymatic activity as monitored by its NADPH consumption (absorption [Abs] change). In the presence of espin alone (+Espin), fascin alone (Rajan et al., 2023c), or espin + fascin (not shown), Mical’s enzyme activity shows no significant difference from Mical alone (Mical only). However, Mical’s enzyme activity is notably stimulated by unbundled, espin-bundled, and espin–fascin co-bundled F-actin. Yet, also note that Mical’s enzyme activity in the presence of espin-bundled and espin–fascin co-bundled F-actin is decreased compared with its activity in the presence of unbundled F-actin. Results of a representative experiment are shown in 1, and the corresponding change in absorbance (mean ± SEM) from *n* = 2 independent experiments/conditions is shown in 2. [F-actin] = 9.2 μM, [Espin] = 4.6 μM for espin only bundles, [Fascin] = 2.3 μM, [Espin] = 2.3 μM for espin + fascin co-bundled F-actin, [Mical] = 0.15 μM, and [NADPH] = 200 μM. **(C)** Low-speed sedimentation of unoxidized actin and Mox-actin shows that fascin, espin, and fascin + espin poorly bundles Mox–F-actin. (Left) SDS-polyacrylamide gel of S, soluble (G-actin); P, pellet (F-actin) fractions of actin solutions after their low-speed sedimentation with fascin, espin, and fascin + espin. (Right) Pelleted F-actin and Mox–F-actin percentage (mean ± SEM) in the presence of fascin, espin, and fascin + espin. [Actins] = 5 µM, [Fascin] = 1 μM for fascin only bundles, [Espin] = 1 μM for espin only bundles, and [Fascin] = 0.5 μM and [Espin] = 0.5 μM for combined bundles. *n* = 3 independent experiments/conditions. **(D)** A model for Mical’s effects on complex bundled F-actin—and Mical and cofilin’s synergistic F-actin disassembly effects on them. (1) Mical binds, becomes activated by, and oxidizes bundled F-actin, producing Mox-actin—which promotes F-actin disassembly and decreased bundling. (2) Actin oxidation by Mical occurs at Met44 and Met47 (in the D-loop of actin) (Hung et al., 2011; Rajan et al., 2023c). This leads to conformational changes in the D-loop of actin and weakens/destabilizes interactions between adjacent filaments subunits (Grintsevich et al., 2017). Cofilin is well-known to sense the conformation of the D-loop (Muhlrad et al., 2004). Additionally, Mical-induced conformational changes in actin filaments are known to enhance cofilin binding, which increases the size of cofilin clusters on them (Grintsevich et al., 2016; Wioland et al., 2021). (3) Cofilin-decorated regions on F-actin are over-twisted compared with undecorated regions, causing its structural instability (Bobkov et al., 2004; Bobkov et al., 2006; Huehn et al., 2020; Lappalainen et al., 2022; Ono, 2020), such that the boundaries between cofilin clusters are filaments’ severing sites (Andrianantoandro and Pollard, 2006; Lappalainen et al., 2022; Ono, 2020; Suarez et al., 2011). These attributes of cofilin, coupled with its increased binding to Mical-oxidized F-actin (Grintsevich et al., 2016; Wioland et al., 2021)—which has weaker inter-longitudinal interactions (Grintsevich et al., 2017)—allow cofilin to more rapidly sever and depolymerize Mox-actin filaments (Grintsevich et al., 2016; Grintsevich et al., 2017; Rajan et al., 2023c; Wioland et al., 2021). Also, the product of Mical’s F-actin disassembly is Mox-actin, which poorly polymerizes (Grintsevich et al., 2016; Hung et al., 2011) and is also more poorly bundled, compared with unoxidized F-actin, by fascin, espin, and fascin + espin (present study). Thus, Mox-actin is ineffectively reused for F-actin elongation or bundling, further increasing F-actin disassembly. Source data are available for this figure: SourceData F7.

In light of our identification above of molecular mechanisms that robustly disassemble complex F-actin bundles, we explored their biochemical basis. MICALs are oxidoreductase (redox) enzymes that use actin filaments as a substrate, acting on them through a catalytic posttranslational mechanism (Hung et al., 2011; Rajan et al., 2023b). In particular, MICALs bind F-actin, and in the presence of their coenzyme (NADPH), they are enzymatically active and oxidize actin’s methionine (M) residues M44 and M47 (producing Mical-oxidized actin [Mox-actin]), which destabilizes F-actin (Fig. 7 A [Hung et al., 2011; Rajan et al., 2023b]). We found that Mical requires NADPH to disassemble espin and espin–fascin co-bundled F-actin (Fig. 3, A and B), indicating that Mical uses its catalytic mechanism to disassemble also these different types of bundled filaments. We therefore looked at whether espin-bundled and espin–fascin co-bundled F-actin are indeed a Mical substrate. In the absence of a substrate, Mical consumes slowly its NADPH coenzyme (Fig. 7 B [Hung et al., 2011]). Adding espin alone or espin + fascin to Mical did not significantly increase its NADPH consumption rate (Fig. 7 B), showing that they are not Mical substrates. However, similar to unbundled F-actin, espin-bundled and espin–fascin co-bundled F-actin enhanced Mical’s NADPH consumption (Fig. 7 B). Notably, these results add to our previous observations that fascin-bundled F-actin also enhances Mical’s NADPH consumption (Rajan et al., 2023c). These results demonstrate that F-actin bundled with espin, and complex bundles composed of espin + fascin, similar to unbundled F-actin and fascin-bundled F-actin, are Mical substrates.

**Figure S5.**
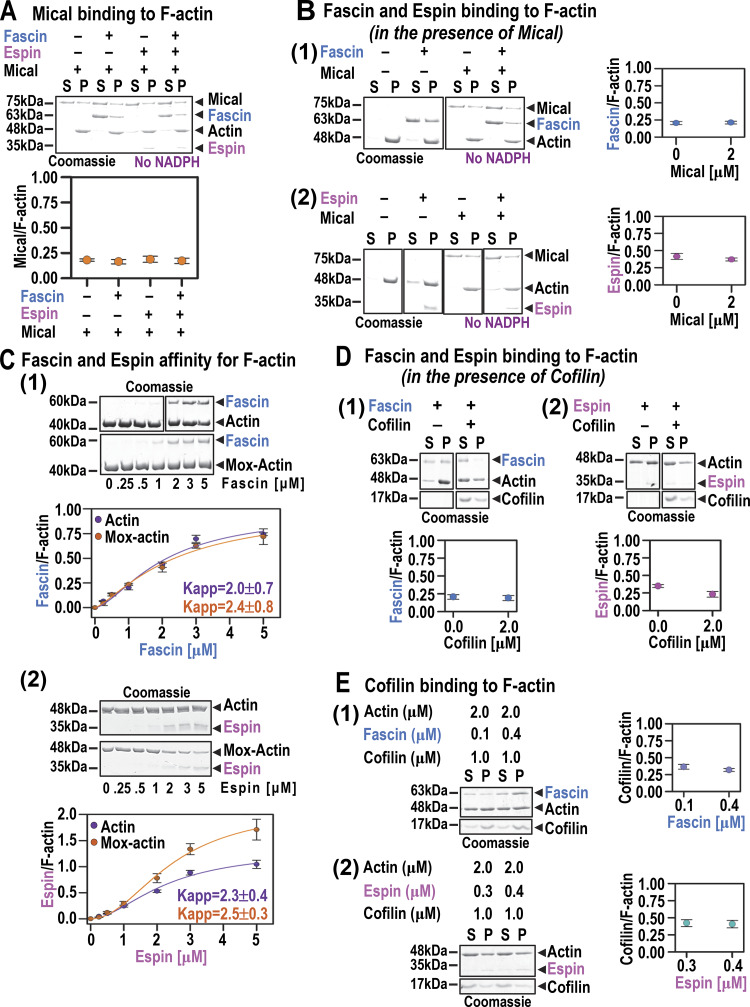
**Further analysis of the biochemical mechanism underlying Mical and cofilin’s effects on bundled forms of F-actin. (A)** Mical’s association with F-actin is not altered in the presence of fascin, espin, or both. (Top) Coomassie-stained SDS-polyacrylamide gel of S, soluble (G-actin); P, pellet (F-actin) fractions after their high-speed sedimentation. (Bottom) The ratio of Mical to actin in pellets (mean values ± SEM) obtained by densitometric analysis of SDS-PAGE gel bands. [F-actin] = 5 μM; [Fascin] = 2 μM; and/or [Espin] = 2 μM; [Mical] = 2 μM. No NADPH present; *n* = 2 independent experiments/conditions. Please note that for the sake of comparison for the experiments in B that we have added some of the lanes from A to B. See B for more information. **(B)** Neither fascin (1) nor espin’s (2) association with F-actin is altered in the presence of Mical (without NADPH). Left side in 1 and 2: Coomassie-stained SDS-polyacrylamide gel of S, soluble (G-actin); P, pellet (F-actin) fractions after their high-speed sedimentation. Right side in 1 and 2: The ratio of fascin (1) and espin (2) to actin in pellets (mean values ± SEM) in the absence or presence of Mical without NADPH obtained by densitometric analysis of SDS-PAGE gel bands. [Actin] = 5 μM; [Fascin] = 2 μM; [Espin] = 2 μM; [Mical] = 2 μM. No NADPH present; *n* = 2 independent experiments/conditions. Note that gel lanes from A were used in B but quantified differently (i.e., Mical binding to actin in the presence of fascin and/or espin [A] and fascin or espin binding to F-actin in the presence of Mical [B]). For ease of comparison among each other, they are shown in both A and B. Also, since the fascin without Mical and espin without Mical experiments were run on the same gel in the presence of F-actin only, the same actin only lanes are shown in 1 and 2. **(C)** Fascin (1) and espin (2) have a similar affinity for Mox-actin and unmodified actin. Top panels in 1 and 2: SDS polyacrylamide gels of pellet fractions of actin and Mox-actin samples after their high-speed sedimentation. Bottom panels in 1 and 2: Plots of bundling protein/F-actin versus total bundling protein concentration as obtained from the analysis of SDS polyacrylamide gels shown in the top panels. K_app_ values of 2.4 ± 0.8 μM versus 2.0 ± 0.7 μM (1) and 2.5 ± 0.3 μM versus 2.3 ± 0.4 μM (2) were determined for these bundling proteins for Mox–F-actin and F-actin, respectively. This indicates that fascin and espin have a similar affinity for unmodified F-actin and Mox–F-actin. [Actin] = 5 μM; [Fascin] or [Espin] = as shown in the figure. *n* = 2–4 independent experiments/condition. Mean ± SEM. **(D)** Low-speed pelleting assays show that the association of fascin (1) or espin (2) with F-actin is not altered in the presence of cofilin (note that the amount of F-actin in the pellet decreases in the presence of cofilin due to its disassembly effects on fascin- and espin-bundled F-actin) (as shown in Fig. 5 using other assays). Top panels in 1 and 2: Coomassie-stained SDS-polyacrylamide gel of S, soluble (G-actin); P, pellet (F-actin) fractions after their low-speed sedimentation. Bottom panels in 1 and 2: The ratio of fascin (1) or espin (2) to actin in pellets (mean values ± SEM) in the absence or presence of different concentrations of cofilin as obtained by densitometric analysis of SDS-PAGE gel bands. [F-actin] = 2 μM; [Fascin] or [Espin] = 0.4 μM; [cofilin] = as indicated in the figure; *n* = 2–4 independent experiments/conditions. **(E)** Low-speed pelleting assays show that at the fascin and espin concentrations used in our Mical + cofilin disassembly assays the association of cofilin with F-actin is not altered in the presence of fascin or espin. Left panels in 1 and 2: Coomassie-stained SDS-polyacrylamide gel of S, soluble (G-actin); P, pellet (F-actin) fractions after their low-speed sedimentation. Right panels in 1 and 2: The ratio of cofilin to actin in pellets (mean values ± SEM) in the presence of different concentrations of fascin (1) or espin (2) obtained by densitometric analysis of SDS-PAGE gel bands. [F-actin] = 2 μM; [cofilin] = 1 μM; [Fascin] and [Espin] = as indicated in the figure; *n* = 3 independent experiments/conditions. Source data are available for this figure: SourceData FS5.

We therefore further explored the biochemical mechanisms through which Mical’s enzymatic activity disassembles these complex bundles. Notably, no differences were observed in Mical binding to espin-bundled, fascin-bundled, or espin–fascin co-bundled F-actin versus unbundled F-actin (Fig. S5 A). Also, Mical (in NADPH’s absence) did not affect espin or fascin’s F-actin binding (Fig. S5 B). Thus, Mical in the absence of its enzymatic activity does not alter fascin’s binding/unbinding dynamics. However, we found that once Mical oxidizes F-actin, although fascin and espin’s affinity for Mox–F-actin is similar to that for unoxidized F-actin (Fig. S5 C), this Mox–F-actin is poorly bundled by espin and espin + fascin (Fig. 7 C). These results, coupled with previous results that Mox–F-actin is highly unstable and Mox–G-actin only poorly polymerizes (Grintsevich et al., 2016; Grintsevich et al., 2017; Hung et al., 2011), provide a biochemical understanding of how Mical disassembles complex bundled filaments. Namely, Mical, in the presence of its NADPH coenzyme, oxidizes actin within complex bundles. This destabilizes F-actin bundles, leads to a decrease in F-actin that espin and fascin can bundle well, and produces Mox–G-actin that cannot readily polymerize (Fig. 7 D (1)).

### Biochemical mechanisms underlying Mical and cofilin’s synergy in complex F-actin bundles disassembly—and fascin and espin’s protection against it

Next, we explored how cofilin and Mical synergize in disassembling these complex bundles. Cofilin targets aged, ADP-enriched F-actin segments (Chin et al., 2016; McCullough et al., 2011; Suarez et al., 2011). It binds cooperatively to F-actin, alters its twist (shortens their helical pitch), and rearranges its inter-protomer contacts, resulting in F-actin disassembly (Bobkov et al., 2002; Bobkov et al., 2004; De La Cruz and Sept, 2010; Galkin et al., 2003; Wioland et al., 2017). We found that cofilin concentrations well above those used in our Mical + cofilin disassembly assays (Fig. 5 and Fig. S4) did not affect espin or fascin F-actin binding (Fig. S5 D). Thus, cofilin does not assist Mical’s bundled F-actin disassembly by preventing bundling proteins from interacting with F-actin. Similarly, cofilin did not increase its binding to bundled F-actin at the fascin and espin concentrations used in our Mical + cofilin disassembly assays (Fig. S5 E). These results are consistent with previous results with fascin and cofilin loading (Breitsprecher et al., 2011; Chikireddy et al., 2024) and indicate that fascin and espin do not assist Mical by increasing cofilin loading onto F-actin. In contrast to that, although Mical alone does not affect cofilin’s F-actin binding, Mical’s F-actin oxidation has been found to increase cofilin’s interaction with F-actin (Grintsevich et al., 2016; Grintsevich et al., 2017; Rajan et al., 2023c; Wioland et al., 2021). These results, therefore present a model consistent with Mical’s effects on unbundled and fascin-bundled F-actin. Namely, Mical’s F-actin oxidation alters F-actin’s conformation and stability—including weakening espin and fascin–F-actin bundling (Fig. 7 D (1)), which increases cofilin’s binding and allows it to disassemble Mox-F-actin more rapidly than the unoxidized F-actin (Fig. 7 D (2 and 3)).

We also sought to understand the mechanisms through which these complex co-bundled filaments are more protected from disassembly by Mical. Notably, although espin-bundled and espin–fascin co-bundled F-actin enhanced Mical’s enzymatic activity (Fig. 7 B), they did so to a lesser extent than unbundled F-actin (Fig. 7 B). Yet, we did not observe any difference in Mical’s binding to espin or espin–fascin co-bundled F-actin versus unbundled F-actin (Fig. S5 A). Thus, these results indicate that espin and fascin’s combined presence in bundled F-actin helps to protect F-actin from Mical-induced disassembly by decreasing Mical’s enzymatic action on F-actin. These effects, in combination with a proposed decreased accessibility of Mical to individual filaments within the thicker bundles produced by espin + fascin (compared with espin and fascin alone), provide mechanisms underlying the increased protection of these complex bundles from Mical + cofilin.

### Fascin and espin together help protect actin bundles from Mical-mediated disassembly *in vivo*

**Figure 8.**
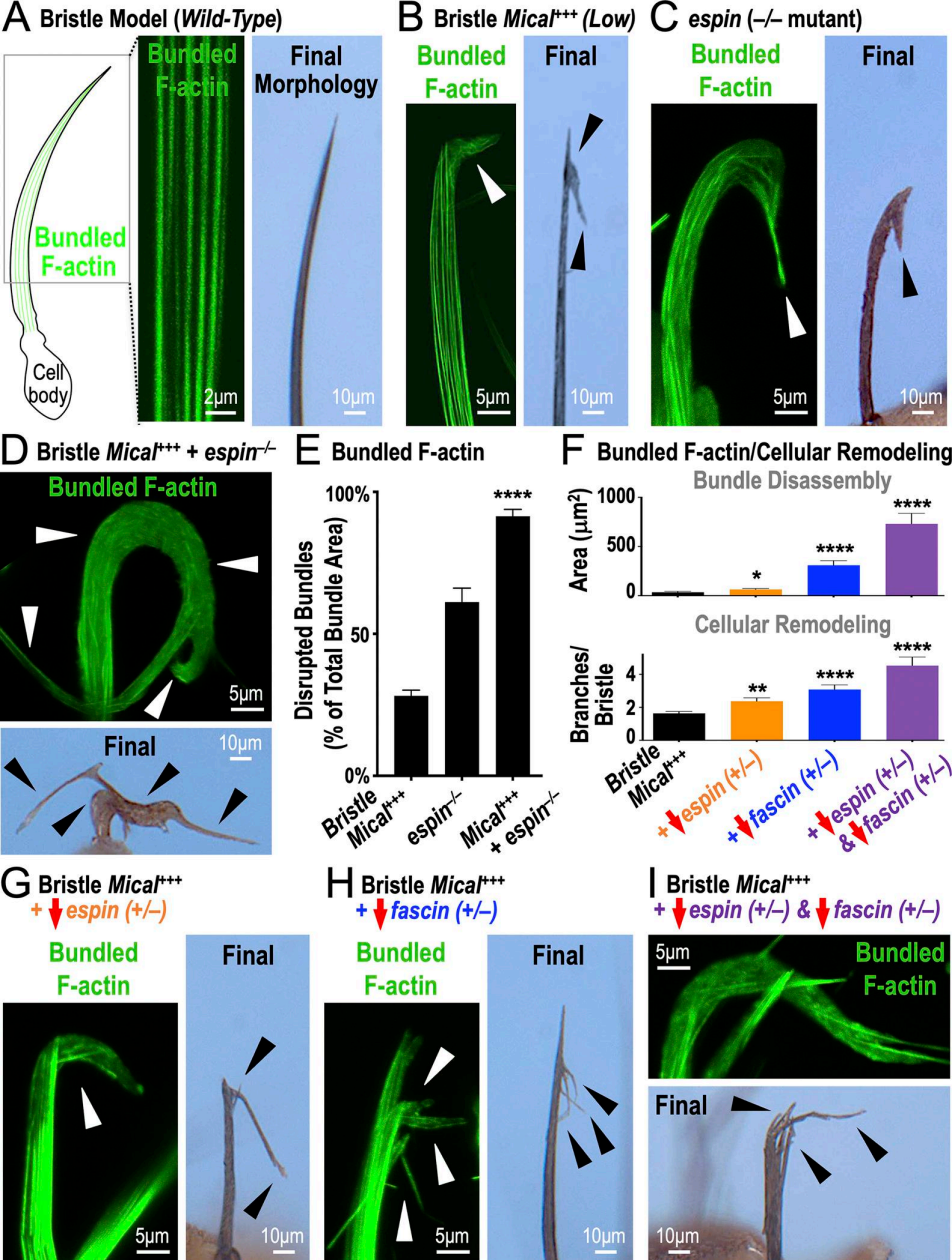
**Espin and fascin combine to help protect F-actin bundles against Mical-mediated disassembly and cellular remodeling *in vivo*. (A)** *Drosophila* bristle cells contain bundled actin filaments (green) cross-linked with bundling proteins (espin and fascin) that stabilize a long unbranched bristle extension. Dashed rectangular region is magnified in the adjacent images of bundled F-actin (middle) and the final bristle morphology (right). **(B)** Expressing low levels of Mical in bristle cells (bristle *Mical*^+++^ [Low]) results in bundled F-actin disassembly at the bristle tip (left, arrowhead) that induces localized remodeling to generate a branch (right, arrowheads). **(C–E)** Espin regulates Mical’s disassembly effects on bundled F-actin *in vivo*. **(C)** Loss of *espin* (–/– mutant) results in bristles with a loss of bundled F-actin, including that they have a branch at the tip (arrowhead), and they resemble the bristles seen after increasing the levels of Mical (B). **(D and E)** In comparison with *espin*^*–/–*^ mutant alone or bristle *Mical*^+++^ (low) alone, increasing bristle Mical levels in an *espin*^*–/–*^ mutant background results in a significant increase in the loss of bundled F-actin (D and E) and cell shape changes (e.g., compare arrowheads in D to B and C). *n* = 6 bristle cells assessed across six animals. Mean ± SEM for graph, ****P < 0.0001, unpaired *t* test (two-tailed). **(F–I)** Espin, fascin, and espin + fascin together regulate Mical’s disassembly effects on bundled F-actin *in vivo*. **(F and G)** Decreasing *espin* levels (*espin* +/−) significantly enhance Mical’s ability to disassemble bundled F-actin and induce cellular remodeling (graph, image [e.g., compare arrowheads between G and B]). **(F and H)** Decreasing *fascin* levels (*fascin* +/−) also significantly enhance Mical’s ability to disassemble bundled F-actin and induce cellular remodeling—including having a stronger effect on this ability of Mical compared with espin (graph, image [e.g., compare arrowheads between H and B and G]). **(F and I)** Mical’s ability to disassemble bundled F-actin and induce cellular remodeling becomes significantly more drastic (e.g., graphs, bundled F-actin image, and arrowheads) when the levels of *espin* and *fascin* are both decreased (*espin* +/− and *fascin* +/−). On their own, *espin* +/−, *fascin* +/−, and *espin* +/− and *fascin* +/− look normal/“wild-type” (i.e., 0 in both graphs in F, not shown). *n* > 10 bristle cells assessed across 10 animals per genotype. Mean ± SEM for graphs, *P < 0.05, **P < 0.01, ****P < 0.0001, and unpaired *t* test (two-tailed).

Lastly, we turned to *in vivo* assays to explore further the mechanisms underlying complex bundled F-actin disassembly. F-actin *in vivo* is often found simultaneously bundled with different bundling proteins (Rajan et al., 2023a). F-actin in *Drosophila* bristle cells, for example, is well-known to associate with multiple bundling proteins—most notably espin and fascin—and it is a model for studying these complex F-actin bundles *in vivo* (Fig. 8 A [Tilney and DeRosier, 2005]). Notably, current models of bristle formation indicate espin appears before fascin and initiates F-actin assembly into bundles (Tilney and DeRosier, 2005). Fascin then further bundles/stabilizes espin-bundled filaments and organizes them into maximally cross-linked F-actin (Tilney and DeRosier, 2005). Removal of either *espin* or *fascin* (*Drosophilaforked* or *singed–/–* mutants, respectively) generates similar types of bristle morphological defects (see below [Tilney and DeRosier, 2005]).

Based on the results of our *in vitro* experiments, we set up *in vivo* experiments using this bristle model system to examine Mical’s effects on the disassembly of espin-bundled, fascin-bundled, and espin–fascin co-bundled F-actin. High Mical levels robustly disassemble bundled F-actin in bristle cells, such that there is very little normal structure remaining in them (Hung et al., 2010). Thus, for better quantification of Mical’s effect on different bundled filaments, we reduced its levels (similar to what we have done *in vitro* [above] to quantify Mical’s effects on different bundled F-actin). Expressing low Mical levels in bristles results in a low-level of bundled F-actin disassembly that induces localized remodeling to generate a branch (Fig. 8 B [Hung et al., 2010]). Removing *espin* (using a CRISPR-generated *espin* mutant knockout [–/–] [Baskar et al., 2019]) also results in bundled F-actin disassembly with effects on bristle extension and shape (Fig. 8 C). Notably, this effect of decreasing *espin* levels strongly resembles the effect of increasing *Mical* levels—including F-actin disassembly and remodeling that generates a branch (compare Fig. 8, B and C). Thus, increasing Mical levels and decreasing espin levels result in similar observable traits (i.e., they phenocopy one another), supporting that they are part of the same genetic pathway. Moreover, removing *espin*, in a background in which low levels of Mical are expressed in bristles, significantly increased Mical’s *in vivo* effects—resulting in increased Mical-mediated bundled F-actin disassembly and cellular remodeling (compare Fig. 8, B–E). Therefore, as we observed with purified proteins, our *in vivo* results also indicate that espin bundling helps protects F-actin from Mical’s disassembly.

We thus tested whether espin and fascin work together to help protect F-actin from Mical *in vivo*. Small decreases in *espin* levels (i.e., *espin^+/−^* heterozygotes) do not produce any observable defects on their own (i.e., these flies look normal/“wild-type”). However, in contrast to that, we found that even small decreases in *espin* levels *in vivo* significantly increased Mical’s ability to disassemble bundled F-actin and remodel cells (Fig. 8, F and G). We also observed similar effects when we decreased *fascin* levels *in vivo*—including that small decreases in *fascin* levels, which did not produce any observable defects on their own, significantly increased Mical’s disassembly/remodeling effects *in vivo* (Fig. 8, F and H). So too, similar to what we observed with purified proteins, changing the levels of fascin alone, compared with changing the levels of espin alone, more strongly altered Mical’s disassembly/remodeling effects (Fig. 8 F). We found also that simultaneously lowering the levels of both *espin* and *fascin*, which did not produce any observable defects on their own, increased even more Mical’s effects on bundled F-actin and cellular remodeling (Fig. 8, F–I). Thus, Mical works better to disassemble bundled F-actin *in vivo* when espin and fascin levels are both decreased. Likewise, fascin and espin help protect F-actin from this disassembly more effectively together than alone. Therefore, as we saw with purified proteins, espin and fascin combine *in vivo* to more robustly protect F-actin against its Mical-mediated disassembly and remodeling.

## Discussion

**Figure 9.**
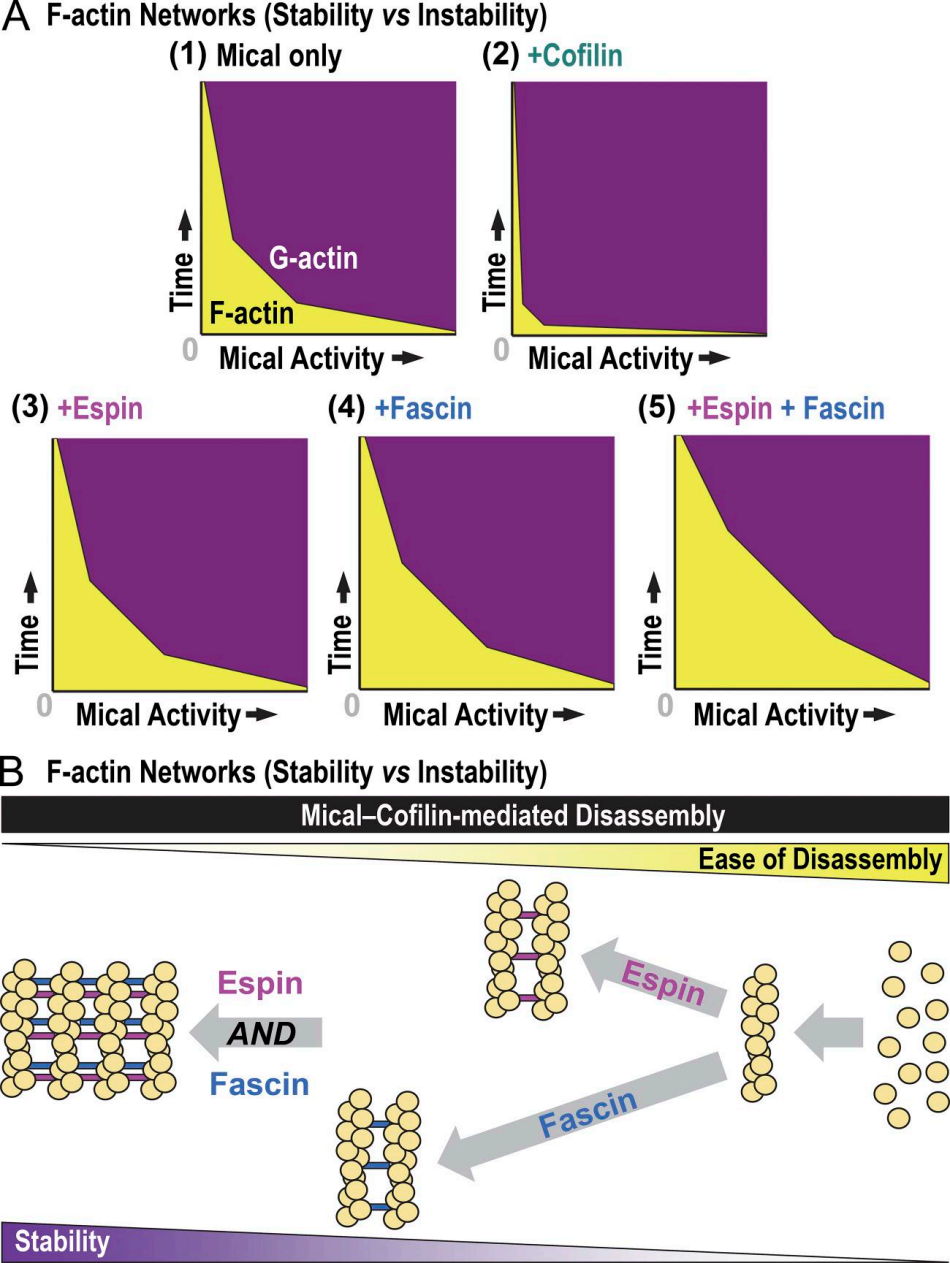
**Summary and model: F-actin network stability and instability. (A)** Summary. (1) Mical in the presence of its NADPH coenzyme (Mical activity) destabilizes unbundled, single protein (espin or fascin) bundled, and multi-protein (espin + fascin) co-bundled F-actin networks. This destabilization increases with increasing Mical activity and time. (2) Mical-induced destabilization of unbundled, bundled, and complex co-bundled F-actin networks synergistically increases in the presence of cofilin. (3 and 4) In the presence of bundling proteins, espin (3) and fascin (4), Mical (and Mical + cofilin’s) destabilizing effects are dampened (e.g., compare 1–3 and 4). (5) This bundler-induced increased protection against Mical (and Mical + cofilin’s) destabilizing effects, synergically increases in the presence of both espin and fascin (e.g., compare 5 to 1, 3, and 4). **(B)** Model. Our results with purified proteins and *in vivo* support the view that espin and fascin work together to stabilize F-actin networks—such that when present together in tandem, F-actin networks are more stable than when espin or fascin are present at equal concentrations on their own. Our results with purified proteins and *in vivo* also support the point that this dual bundling protein-mediated stability helps protect F-actin networks from Mical and cofilin’s synergistic disassembly effects. Lowering the levels of espin and/or fascin makes F-actin networks more susceptible to Mical–cofilin-mediated disassembly. Our results also reveal that this balance between espin–fascin-mediated F-actin bundling and Mical-mediated disassembly is critical for maintaining and regulating normal cellular morphology *in vivo*.

Roles of actin bundles, including why they are bundled often simultaneously and seemingly redundantly, by different proteins, is incompletely understood. It is unknown also how these complex, multi-protein–bundled, stable structures are disassembled to allow for their remodeling per functional needs. We find here that two of the most common bundlers, fascin and espin, synergize to form actin bundles—forming wider, denser, and longer bundles than through the sum of their individual effects. We also find that these complex, multi-protein—bundled F-actin structures are effectively destabilized by a synergism of Mical with cofilin (Fig. 9). Our results also reveal that together fascin and espin help protect F-actin from this disassembly more effectively than each one of them alone (Fig. 9). This interplay between multiple bundling proteins and F-actin disassemblers is critical for cellular remodeling *in vivo* (Fig. 9).

We speculate that the mechanisms underlying espin and fascin’s synergy result from an intersection of their individual properties. Our results support previous observations that fascin and espin (splice forms 3A and 3B) simultaneously bind to F-actin in overlapping patterns (Chanut-Delalande et al., 2006; Chou et al., 2011; Otani et al., 2016; Roy and Perrin, 2018; Vignjevic et al., 2006; Winkelman et al., 2016). Our results also support previous observations that fascin and espin (3A) bundle F-actin in a similarly effective manner (Lieleg et al., 2009), including that they both induce F-actin twisting (∼1° per actin monomer), which may underlie their close coexistence in bundles (Claessens et al., 2008; Gong et al., 2025; Purdy et al., 2007; Shin et al., 2009). Yet, differences also exist between these bundlers—including that espin induces stiffer and more twisted bundles, while fascin produces more flexible bundles (Claessens et al., 2008; Purdy et al., 2007; Shin et al., 2009). Fascin is also more effective at bundling filaments that are pre-aligned (Courson and Rock, 2010; Sherer and Courtemanche, 2022; Tilney et al., 1998; Winkelman et al., 2016; Yang et al., 2013). Thus, we propose that the synergism we observe occurs because espin helps to gather, tether, and rigidly bundle actin filaments and thereby align them to facilitate enhanced fascin-mediated bundling.

Our synergistic results are also supported by several observations: (1) espin is highly expressed prior to fascin in cells that generate espin–fascin bundles (Chanut-Delalande et al., 2006; Guild et al., 2003; Guild et al., 2005; Roy and Perrin, 2018; Tilney et al., 2000; Wulfkuhle et al., 1998), (2) espin and fascin generate together larger/more expanded F-actin bundles *in vitro* and *in vivo* (Chou et al., 2011; Claessens et al., 2008; Krishnan et al., 2021; Tilney and DeRosier, 2005; Velez-Ortega and Frolenkov, 2019; Winkelman et al., 2016), and (3) *espin* and *fascin* mutants generally phenocopy each other (Dickinson and Thatcher, 1997; Guild et al., 2005; Okenve-Ramos and Llimargas, 2014; Tilney and DeRosier, 2005; Velez-Ortega and Frolenkov, 2019), but cannot substitute for one another (Cant and Cooley, 1996). This indicates that they require each other *in vivo*.

Our results provide also important insights into disassembly of F-actin structures that are bundled together with multiple bundlers. Bundled F-actin, including that bundled with fascin or espin, is notably resistant to disassembly (Blanchoin et al., 2014; Breitsprecher et al., 2011; Chikireddy et al., 2024; Elkhatib et al., 2014; Huang et al., 2005; Loomis et al., 2003; Michelot et al., 2007; Mizuno et al., 2018; Schmoller et al., 2011; Yang et al., 2022). We previously found that Mical, and Mical in combination with cofilin, rapidly and extensively disassembles fascin-bundled F-actin (Hung et al., 2010; Rajan et al., 2023c). We now find that both Mical and cofilin, and Mical in combination with cofilin, rapidly and extensively disassembles espin-bundled F-actin, as well as complex bundles containing both fascin and espin. These observations provide critical insights into the mechanisms of remodeling complex and highly stabilized cellular actin networks.

It is also noteworthy that espin and fascin have different expression patterns (Rajan et al., 2023a), timing of expression (as mentioned above), and subcellular locations (Krishnan et al., 2021; Tilney and DeRosier, 2005; Velez-Ortega and Frolenkov, 2019; Vignjevic et al., 2006). Thus, their spatiotemporal overlap may promote structural stability in specific types of cells, subcellular regions, and/or at different ages. For example, in some cells, fascin localizes along the length of filopodia, while espin is predominantly in the proximal parts of filopodia (Vignjevic et al., 2006). Thus, because of these regions of espin–fascin overlap, the proximal parts of these filopodia would likely be more protected from Mical–cofilin-driven disassembly than distal regions. Interestingly, semaphorin repulsive guidance cues employ Mical–cofilin to exert their F-actin disassembly effects (Grintsevich et al., 2016; Hung and Terman, 2011; Rajan et al., 2023b), and they have been found to more strongly disassemble distal versus proximal filopodial regions (Fan et al., 1993; Hung and Terman, 2011). Thus, our data suggest specific mechanisms that limit the effects of these critical repulsive guidance cues.

Our *in vivo* work within the bristle model system supports our *in vitro* results and indicates that a fine-tuned balance between the assembly and disassembly of actin bundles is essential for proper cell functions. Notably, pathologies including carcinomas and neurological abnormalities are linked to dysregulation of bundlers, including fascin and espin (Rajan et al., 2023a). So too, dysregulation of disassembly proteins, including Mical and cofilin, is linked to the same abnormalities (Rajan et al., 2023b). One notable example of such dysregulation is in the stereocilia of inner hair cells—the mechanosensory structures required to transduce sound into sensory information in vertebrates that share functional and structural similarities with *Drosophila* bristles (Muller and Littlewood-Evans, 2001). Fascin, espin, cofilin, as well as other proteins important for MICAL’s function, such as myosin Myo15 and the MsrB/SelR reductase that reverses MICAL’s effects (Rajan et al., 2023b), are required for normal hearing (Park and Bird, 2023). Thus, an interplay between these proteins is likely to not only affect *Drosophila* bristles but also multiple other cell types in mammals, including humans.

In conclusion, our results not only elaborate further the unique individual contributions of F-actin bundlers to bundle structure but also their ability to synergize with each other. Although in some cases bundling proteins can substitute for each other *in vivo* (Cant et al., 1998; Davidson et al., 2019; Revenu et al., 2012), our observations support results that each protein has its unique contributions to F-actin bundling (Bartles, 2000; DeRosier and Tilney, 2000; Park and Bird, 2023; Rajan et al., 2023a). Indeed, bundling proteins are often co-expressed, and their specific combinations—such as of fascin and α-actinin (Bashirzadeh et al., 2020; Claessens et al., 2008; Schmoller et al., 2008; Tseng et al., 2002; Tseng et al., 2005; Winkelman et al., 2016), fascin and plastin/fimbrin (Van Audenhove et al., 2016), α-actinin and filamin (Esue et al., 2009), and Eps8 and IRSp53 (Disanza et al., 2006)—provide new attributes to F-actin networks. Such combinations (including synergistic interactions) may be a general feature of bundling proteins, allowing them (despite their similar bundling function) to provide specific cells and subcellular regions with different biological properties—such as increased stability. Along these lines, it is also intriguing that larger espin splice forms (espin-1 and espin-2) form thicker actin bundles than espin-4 (the splice form used herein) and have higher F-actin affinity (∼3×) and stronger actin-bundling activity (∼2.5×) due to the presence of additional domains (Bartles et al., 1998; Belyantseva et al., 2025; Chen et al., 1999; Zheng et al., 2014). However, large espin splice forms are autoinhibited, thus limiting their abilities in the absence of activators (Liu et al., 2016; Zheng et al., 2014). Therefore, different splice forms of bundling proteins provide additional means to increase bundles stability.

We also identify specific molecules and mechanisms that allow for the disassembly and remodeling of these multi-protein–bundled actin structures. Thus, our results indicate that a fine-tuned mechanism is likely to exist in all cells to control: (1) the extent of F-actin cross-linking by multiple bundling proteins, which stabilizes bundles and decreases cellular remodeling, and (2) the extent of disassembly proteins activation, which drives decreased bundles stabilization/increased cellular remodeling. It seems likely that specific conditions may alter this balance and lead to disorders/diseases that could selectively affect different tissues. Thus, this balance should be further investigated—including the roles of other bundling proteins and their combinations (including different splice forms) in bundles topology and stability and their interplay with Mical and cofilin disassembly proteins.

## Materials and methods

### Plasmids

*Drosophila* fascin and *Drosophila* Mical (Mical^RedoxCH^) DNA constructs described by Hung et al. (2010); Rajan et al. (2023c) were used. Human cofilin-1 plasmid was a kind gift from Dr. E.M. De La Cruz (Yale University School of Medicine, New Haven, CT, USA). These particular constructs were used to compare this study with our previous results using these constructs (Rajan et al., 2023c). A protein expression plasmid containing the espin splice variant espin-4 (small espin), which has the domains needed to rescue the *in vivo* defects that result from a loss of *Drosophila espin* (forked) (Grieshaber et al., 2001), was kindly provided by Dr. J.R. Bartles (Northwestern University Feinberg School of Medicine, Chicago, IL, USA) (Bartles et al., 1998).

### Protein purification

Actin was either purified from rabbit skeletal muscle acetone powder (Pel-Freez) as described by Spudich and Watt (Spudich and Watt, 1971) or obtained from Cytoskeleton, Inc. In brief, lyophilized acetone powder was resuspended in a Pre-G-buffer (5 mM Tris-HCl, pH 8.0, 0.2 mM CaCl_2_ supplemented with 0.2 mM ATP, 1 mM DTT, and 0.4 mM PMSF), then cleared by centrifugation for 1 h at 13,000 rpm in a J2-HS rotor, 4°C. The supernatant was filtered through a 0.2-µm syringe filter, and then actin was polymerized by the addition of 2 mM MgCl_2_ and 50 mM KCl for 2 h while on slow stirring at 4°C. Next, more solid KCl was added to a final concentration of 0.6 M, and stirring was continued for another 30 min at 4°C. F-actin was pelleted by centrifugation in a Ti-60 rotor at 48,000 rpm at 4°C for 100 min. The pellet was homogenized by a Dounce homogenizer and then dialyzed versus G-buffer (2 mM Tris-HCl, pH 8, 0.2 mM CaCl_2_, 0.5 mM DTT, and 0.2 mM ATP) at 4°C for 3 days with buffer changes at 24-h intervals. After 4 days, actin was subjected to centrifugation (48,000 rpm, Ti-60 rotor), 4°C for 100 min, and the supernatant was loaded on a size exclusion Superdex S200 16/60 column (Amersham Biosciences) equilibrated in S200 G-buffer (2 mM Tris-HCl, pH 8, 0.2 mM CaCl_2_, 0.5 mM DTT, 0.2 mM ATP, 0.25 mM TCEP, 0.2 mM PMSF, and 0.02% NaN_3_). Collected peak fractions were stored at 4°C. Actin was used within 1 mo of its purification.

Mox-actin was prepared and purified as described (Grintsevich et al., 2016; Rajan et al., 2023c). In particular, 20 µM of actin was polymerized (F-actin) by incubating G-actin in 1× polymerization buffer (diluted from a stock 10× PB buffer: 100 mM HEPES, pH 7.4, 500 mM KCl, 10 mM MgCl_2_, 10 mM EGTA, supplemented with 2 mM ATP and 10 mM DTT) for 1 h at RT. F-actin was then diluted to 2 μM in 1× PB buffer supplemented with NADPH (0.04 μM) and Mical 0.04 μM (50:1 molar ratio of actin to Mical) and incubated for 1 h at RT. After an hour, the solution was centrifuged at 321,530 × *g* for 20 min at 4°C, and the supernatant (containing Mox-actin) was mixed for 30 min with 250 μl of HisPur Ni-NTA Resin (Thermo Fisher Scientific) equilibrated with G-buffer to remove the residual Mical enzyme. Then the flow-through was collected and dialyzed overnight against S200 G-buffer. Before using Mox-actin in our experiments, its oxidation was confirmed by a subtilisin digestion assay as described (Grintsevich et al., 2016; Rajan et al., 2023c). In particular, G-actin and Mox-actin were digested with subtilisin (we used 1:1,000 [wt/wt] ratio of subtilisin:actin) in a G-buffer for 15 min. Subtilisin cleaves G-actin between residues 47 and 48 (Schwyter et al., 1989), while Mox-actin remains uncleaved, indicating its modification by Mical at position 47.

*Drosophila* Fascin was purified as previously described (Hung et al., 2010). In particular, fascin was expressed in ArcticExpress (DE3) cells (Agilent Technologies). Cells were grown at 30°C until OD_600_ = 0.6–0.8, followed by an induction with 0.5 mM IPTG for 24 h at 14°C. The protein was purified by passing the lysate through a HisTrapFF affinity column (Cytiva) followed by its elution using 250 mM imidazole buffer. The elutant was digested overnight at 4°C with SUMO proteinase (in house purified [Hung et al., 2010; Rajan et al., 2023c]) and further purified using a HisTrapFF affinity column (Cytiva) to remove the His-SUMO protease. The fractions containing fascin protein were further passed through a HiTrap Q column (Cytiva) equilibrated with buffer (10 mM Tris-HCl, pH 8.0, 1 M NaCl, 5% glycerol, and 1 mM DTT). Under these conditions, fascin does not bind to a HiTrap Q column and was collected from the flow-through and dialyzed overnight in fascin storage buffer (10 mM Tris-HCl, pH 8.0, 50 mM NaCl, 5% glycerol, and 1 mM DTT).

*Drosophila* Mical (Mical^RedoxCH^) was purified as previously described (Hung et al., 2010). Mical^RedoxCH^ was expressed in ArcticExpress (DE3) cells (Agilent Technologies). Cells were grown at 30°C until OD_600_ = 0.6–0.8, followed by an induction with 0.2 mM IPTG, for 24 h, at 14°C. The protein was purified by incubating the lysate with HisPur Ni-NTA Resin column (Thermo), followed by elution with 250 mM imidazole buffer, and digestion with thrombin (Cat. No. 69671-3; Millipore). The digested samples were then bound again to the HisTrap HP column (Cytiva) to separate Mical from the Nus tag, and then passed through HiTrap Q FF column (Cytiva) equilibrated with Q buffer (10 mM Tris-HCl pH 8.0, 10 mM NaCl, 5% glycerol, and 2 mM DTT). Mical was eluted then with a linear gradient of NaCl (0–1 M) in 5 column volumes from the HiTrap Q column. The fractions containing the Mical protein were combined and overnight dialyzed versus Mical storage buffer (20 mM Tris-HCl, pH 8.0, 50 mM NaCl, 5% glycerol, and 2 mM DTT) at 4°C.

Human cofilin-1 (referred to as cofilin in this study) were purified as previously described (Grintsevich et al., 2016; Rajan et al., 2023c). In particular, cofilin was expressed in BL21(DE3)pLysS cells (Agilent Technologies). Cells were grown at 37°C until OD_600_ = 0.6–0.8, followed by an induction with 1 mM IPTG for 4 h at 37°C. Clear cell lysate was loaded onto a DE52 (DEAE cellulose, Sigma-Aldrich) column—sequentially connected to a HiTrap SP FF column (GE) at 4°C. Both columns were equilibrated with 10 mM MOPS, pH 7, supplemented with 1 mM DTT and 0.2 mM PMSF. Cofilin was eluted from the SP FF column with a linear gradient of NaCl (0–700 mM in five column volumes). Cofilin containing fractions were combined and further purified using a size exclusion HiLoad 16/60 Superdex 75 column (Amersham Biosciences) equilibrated with 10 mM MOPS, pH 7, 25 mM NaCl, and 1 mM DTT.

Espin-4 was purified by following the instructions developed by J.R. Bartles (who originally identified espins) (Bartles et al., 1998) and as used by others for this espin splice form (e.g., Claessens et al., 2008; Kitajiri et al., 2010; Purdy et al., 2007). Briefly, espin-4 was expressed in *Escherichia coli* BL21-Codon Plus (DE3)-RIL (Agilent). Cells were grown at 37°C until OD_600_ = 0.6–0.8, followed by an induction with 0.5 mM IPTG, for 8 h, at 25°C. Bacterial pellets were collected by centrifugation and resuspended in an extraction buffer (50 mM Tris-HCl, pH 8.5, 10 mM betamercaptoethanol [βME]) with PMSF and protease inhibitors. The protein was purified by incubating the lysate with HisPur Ni-NTA Resin column (Thermo Fisher Scientific) washed with wash buffer 1 (20 mM Tris-HCl, pH 8.5, 100 mM KCl, 10% glycerol, 20 mM imidazole, and 10 mM βME) and wash buffer 2 (20 mM Tris-HCl, pH 8.5, 1.5 M KCl, 10% glycerol, 20 mM imidazole, and 10 mM βME). The bound recombinant proteins were eluted from the column by a 25–250 mM imidazole gradient. The purified espin was cleaved by 6× His-tagged Tobacco Etch Virus protease (Life Technologies) for 8 h, at 4°C, and the uncleaved protein and protease were removed by passing the solution through a HisPur Ni-NTA Resin column (Thermo Fisher Scientific). Cleaved espin, in the flow-through, was collected and dialyzed overnight into espin storage buffer (10 mM Tris-HCl, pH 7.5, 100 mM KCl, 0.02% NaN_3_, 1 mM DTT, and 10% glycerol). The protein was concentrated using a filter concentrator (Amicon filter, Millipore). Similar results with the espin protein purified in this manner were obtained independently in both the Terman and Reisler labs. We also noted, using standard western analysis approaches with a His antibody (1:1,000; #70796; Novagen) (Hung et al., 2010), that espin was susceptible to degradation (Fig. S1 A (2)). Fresh batches of espin protein were employed to overcome this problem.

All purified proteins except actin were stored at −80°C for futher use.

### Protein labeling

Freshly gel-filtered actin was labeled with Alexa488 succinimidyl ester (SE) (Cat. No. A20000; Life Technologies) as described (Rajan et al., 2023c). In particular, 2 mg of G-actin was polymerized for 1 h, at RT, by adding 2 mM MgCl_2_ and 50 mM KCl. The F-actin (in pellet) was collected after centrifugation at 60,000 rpm (TLA110 rotor) for 30 min at 4°C and dialysized against labeling buffer (50 mM PIPES, pH 6.8, 50 mM KCl, 0.2 mM CaCl_2_, and 0.2 mM ATP) for 3 h at 4°C. After dialysis, actin was incubated with a threefold molar excess of Alexa488SE dye, overnight at 4°C. The labeling reaction was stopped using 1 mM DTT, and the resulting fluorescently labeled F-actin was pelleted (TLA100 rotor, 90,000 rpm, 20 min, 4°C), depolymerized in G-buffer, and gel-filtered on a S200 10/300 Gl equilibrated with S200 G-buffer.

Fascin and espin were labeled on surface cysteines using Cy3-Monomaleimide and Alexa488-Monomaleimide (Invitrogen), which were resuspended in dimethylformamide (DMF) to a concentration of 1 mM. Purified proteins were first dialyzed overnight versus the labeling buffer (20 mM Tris-HCl, pH 7.9, 100 mM NaCl, 0.2 mM EDTA, 0.01% NaN_3_, and 10% glycerol) to remove DTT. A 3 to 5-molar excess of dye was added then to the protein and allowed to label it overnight at 4°C. The labeling reaction was quenched using 1 mM DTT, and the excess dye was removed by three serial dialysis versus 500 ml of the protein storage buffer (labeling buffer + 1 mM DTT) for 2.5 h each.

### Actin filaments/bundled filaments sedimentation (pelleting) assays

For sedimentation assays, samples were centrifuged for 20 min either at 10,000 × *g* (low-speed) or 321,530 × *g* (high-speed). The supernatants and pellets were separated and analyzed on SDS-PAGE. The gels were stained with Coomassie blue and quantified with ImageJ (RRID:SCR_003070) (Schindelin et al., 2012).

10 µM of Mg-ATP F-actin or Mox-F-actin were prepared by first incubating Ca-ATP-G-actin for 3 min in a polymerization ME exchange buffer (0.05 mM MgCl_2_ and 0.2 mM EGTA) to exchange bound Ca^2+^ in G-actin with Mg^2+^. They were then incubated in a 1× polymerization buffer (diluted from a stock 10× PB buffer: 500 mM KCl, 10 mM MgCl_2_, 10 mM EGTA, 100 mM HEPES, pH 7.4, supplemented with 2 mM ATP and 10 mM DTT) for 30 min, at 25°C. Actin filaments were then diluted in 1× PB buffer in the absence or presence of bundling proteins and then incubated at RT for 30 min. The formed F-actin or bundles were incubated with Mical (without NADPH) or cofilin (for binding or bundling assays) or Mical/NADPH (for disassembly assays) for 30 min at RT before centrifugation.

For bundling or binding efficiency measurements (Fig. 1, C and D; and Fig. S5 C), the data collected from three independent experiments were fitted into Hill’s equation (Eq. 1) to determine the affinity of bundling of proteins:

v=fnK+fn, (1)

where v = fraction of the bundling protein bound to actin, [f] = concentration of unbound bundling protein, K = K_D_ (bundling affinity in Figs. 1, C and D) or K_app_ (apparent binding constant in Fig. S5 C), and *n* = Hill cooperativity coefficient.

For a decrease in pelleted actin (as shown in Fig. S3, A (2) and C), the data were normalized individually for each condition (using the value of maximum amount of actin pelleted at that condition without Mical/NADPH), and the percentage change was determined using Eq. 2,

$$\%\;\text{Decrease}=\left[\left(P_{0}-P_{M}\right)\;/\;P_{0}\right]\times 100,$$ (2)

where P_0_ = 1, is the value without Mical/NADPH (after data normalization), and P_M_ = value with Mical/NADPH (after data normalization).

### Light-scattering assays

Actin bundling was monitored by the increase in light scattering at λ = 325 nm after the addition of bundling proteins to solutions of pre-polymerized actin filaments as described by Rajan et al. (2023c). In particular, 5.5 μM F-actin was prepared as described above: (1) by first exchanging bound Ca^2+^ in G-actin with Mg^2+^ and (2) polymerizing by diluting in PB buffer. Bundling protein solutions were added to 5.5 μM F-actin stock solution for a final [F-actin] = 5 µM. For bundles disassembly assays, Mical/NADPH, cofilin, or both, were added to a 5 µM bundled actin (B-actin). All light-scattering measurements were done in triplicates at RT. Notably, buffer addition and solution mixing caused minimal bundles depolymerization (reduction in light scattering), which is reversed with time, due to reformation of actin bundles in the solution (as shown in Fig. S4 B). This reduction in light scattering was considered in the normalization of light-scattering data. Light-scattering changes shown in Fig. 1 E and Fig. 2 B (2)) were determined using Eq. 3,

$$∆I=I-I_{\min}/I_{\max}-I_{\min}$$ (3)

where I = highest intensity seen for a given sample (after data normalization), I_min_ = 1, and I_max_ = 1,200 are the minimum and maximum intensities observed in our experiments after data normalization.

To determine the decrease in light scattering, the data were normalized to a maximum intensity of 1, from which the relative decrease in light-scattering intensity was determined over 15 min for Figs. 4 B (2) and Fig. 5 A, and for 1 min for Fig. 6 A (2).

### Pyrene disassembly assays

Unlabeled and pyrene-labeled actin were mixed with 10× PB buffer to yield a 10 µM, (10% labeled) actin stock in 1× PB buffer. Before polymerization, Ca^2+^ in G-actin was exchanged with Mg^2+^ by incubating actin with 0.2 mM EGTA and 0.1 mM MgCl_2_ for 3 min at RT. The polymerized actin was diluted to 5 µM or 2.5 µM with buffer or buffer with bundling proteins for 1 h. Next, samples of F-actin or bundled-actin were incubated with a buffer containing NADPH, Mical, and/or cofilin. The pyrene fluorescence intensity of actin was monitored in a 96-well fluorescence plate reader (M1000; Tecan Infinite) at 407 nm (excitation at 365 nm). The time delay between mixing of the components and the start of fluorescence data collection was 15–20 s.

### TIRFM

Time-lapse TIRFM was performed according to established protocols (Rajan et al., 2023c; Rajan et al., 2024). In short, 12 µl glass flow cells were prepared using GOPTS-mPEG [(3-Glycidyloxypropyl)trimethoxysilane–methoxy(polyethylene glycol)]-coated coverslips. In paricular, the coverslips were tandem-treated first with GOPTS (Cat No. 440167; Sigma-Aldrich) and subsequently with mPEG amine (Cat No. A3085; JenKem Technology USA). Before that, the flow cells were washed with a blocking buffer (1% Pluronic F127 solution [P2443; Sigma-Aldrich] + 0.1 mg/ml casein) and equilibrated with the TIRF buffer (10 mM HEPES, pH 7.4, 50 mM KCl, 2 mM MgCl_2_, and 0.2 mM EGTA), supplemented with 50 mM DTT, 0.2 mM ATP, 20 mM glucose, and 1% methylcellulose. The on-slide actin polymerization was initiated by introducing into flow chambers 1.0 µM and 20% Alexa488SE actin sample (50 μl) in 1× TIRF imaging buffer (TIRF buffer supplemented with 0.05 mg/ml casein, 0.25 mg/ml glucose oxidase, and 0.05 mg/ml catalase to minimize photobleaching during the imaging [1× TIRF-IB]). After polymerizing actin for 15 min, bundling protein(s) (at final concentrations indicated in the figure legends) were added into the flow cells. Actin bundles were allowed to form on the slides’ surface for 5 min. 5–10 images of the bundles were taken per each condition (as shown in Fig. 2 C).

For Mical/NADPH and/or cofilin-mediated disassembly assays,the on-slide actin polymerization was initiated by introducing 1.0 µM, 20% Alexa488SE actin sample (50 μl) in 1× TIRF-IB into the flow chambers along with different combinations of bundling proteins (as indicated in Fig. 4 C, Fig. 5 B, and Fig. 6 B). After that, 2CV of Mical/NADPH mixtures and/or cofilin (in 1× TIRF-IB) were added into flow chambers, and the reactions were allowed to proceed for 1–2 min before recording the time-lapse TIRFM movies. The movies were recorded using a 3i Vector TIRF System mounted on a Zeiss Axio Observer 7 Basic Marianas Microscope, with a Definite Focus 2 and α Plan-Apochromat 63×/1.46NA Oil TIRF objective M27 and an Andor iXon3 897 512 × 512 10 MHz EMCCD Camera, using Slidebook 6 software (RRID:SCR_01430; Intelligent Imaging Innovations). The movies were recorded for 5–10 min, with a 2.5 s frame interval at RT.

### TIRFM data analysis

All TIRFM data were analyzed using Fiji (ImageJ) software (RRID:SCR_002285; NIH) (Schindelin et al., 2012)—the same as described by Rajan et al. (2023c). In particular, before each analysis, background subtraction was done using rolling ball radius algorithm (ball radius 50 pixels). Bundled actin filaments were randomly chosen from the field of view for analysis, but F-actin bundles present at the edges were excluded from the analysis. Unbundled actin filaments—identified based on their fluorescence intensity—were excluded from the analysis. The length of actin filaments was measured manually by using JFilament plugin in Fiji (JFilament 2D) (RRID:SCR_002285). Fluorescence intensities of bundles were analyzed using the line-scan tool of ImageJ software (RRID:SCR_003070). Average intensity values of internal control unbundled filaments (from the same experiment) were corrected for photobleaching and used to calculate the number of filaments per bundle.

To determine the rates of bundles thinning and shortening during disassembly assays, the photobleaching correction of the movies was performed first. Internal control filaments’ fluorescence intensity from the same experiment was monitored over time to obtain a linear photobleaching curve that was utilized for a photobleaching correction. In the presence of disassembly proteins (Mical/NADPH, cofilin, and Mical/NADPH + cofilin), bundles thinning can occur at any point along their length, either at the ends (“peeling mode” as described by Rajan et al. [2023c]) or between them (i.e., thinning by removal of a small filament segment anywhere in the bundle while the remaining bundle’s width stays the same). To avoid bias and to capture both scenarios, we analyzed entire actin bundles, rather than focusing on specific sites. To determine bundles thinning rates (Fig. 4 C, Fig. 5 B, Fig. 6 B, and Fig. S4, C and E), the entire bundles were selected from the observation area with the region of interest (ROI) manager in Fiji, and their fluorescence intensity values were measured over the entire time of data acquisition using the Analyze tool. We calculated the thinning rates of bundles at two different times: the initial thinning rate (over the first 120 s of imaging) and the overall thinning rate (across the entire data acquisition time). We reasoned that each time frame offers distinct insights. The initial rate reflects how rapidly bundles disassembly begins following the addition of disassembly proteins. It is not affected by potential reassociation of severed fragments later in the reaction. In contrast to that, the overall average rate captures the progression of the entire disassembly process, from start to finish. To determine the initial rates of bundles thinning, data for the first 120 s of imaging were fitted by linear regression (Fig. 4 C (3), Fig. 5 B (3), Fig. 6 B (3), and Fig. S4 E). For total rates determination over the entire time of data acquisition (Fig. S3 B and Fig. S4 D), the intensity data were first normalized and then fitted to the exponential decay model with two parameters, using SigmaPlot 14 (RRID:SCR_003210). Small filaments transiently interacting with the surface—before their rapid disappearance—were not included in the analyses.

The shortening rates of bundles were determined over the first 60 s of imaging after addition of the indicated proteins (Fig. 4 C (2), Fig. 5 B (2), Fig. 6 B (3), and Fig. S4, C and E), with their length measured by using the JFilament plugin in Fiji (JFilament 2D) (RRID:SCR_002285). As above, bundles were identified based on their intensity, and their shrinkage was monitored from the point where single filaments extensions at the bundles end (if present) have depolymerized. When observed, paused bundles were excluded from the analysis. The decrease in length due to severing was also not included. Notably, this shrinkage analysis does not account for the thinning mode of disassembly.

Severing events were marked by bundles breaking into two or more distinct fragments. Bundles severing occurs at the sites of drastic thinning in actin bundles due to the action of the added disassembly proteins (in this case Mical/NADPH, cofilin, or Mical/NADPH + cofilin). Severing rates (breaks/micron/minute) in Fig. 4 C (3), Fig. 5 B (3), Fig. 6 B (3), and Fig. S4, C and E were calculated by measuring the initial length of bundles at t = 60 s (first frame of the video, i.e., the necessary delay between the addition of disassembly proteins and the start of video acquisition), and then manually identifying and counting the severing events frame by frame, starting from the frame t = 62.5 s in the time-lapse images after the flow-in of the indicated proteins and for the next 60 s. The measurements were performed only on 5 µm or longer segments of bundles because shorter fragments (<5 µm) tended to diffuse away—so as in our previous publication (Rajan et al., 2023c), they were thus excluded from the analysis. In the absence of disassembly proteins, bundles length and thickness remain constant during the 60 s in which severing was monitored.

To calculate the percentage of remaining bundles as shown in Fig. 6B (2) (comparing the effects of Mical/NADPH + cofilin on the three different types of bundles [fascin alone, espin alone, or fascin + espin]), we sought to find a quantitative measure that best highlighted the observed differences that Mical/NADPH + cofilin had on different bundle types. For these experiments, we randomly selected 15–20 bundles per bundle type and monitored their length for 150 s after the start of imaging (at intervals of 30 s) using the ROI Manager in Fiji. Bundles or bundles fragments that were ≥7 µm were considered, while the rest were omitted from the final count of the bundles present in the field of view. In particular, since severing of bundles leads to an increase in the total number of fragments, we focused solely on the longer fragments (≥7 µm) to simplify the quantification process. Bundles present on the edge of the field of view were excluded from our analysis. The number of bundles at t = 60 s (first frame of the video) was normalized to 100% (individually for each video, *n* = 3 experiments/conditions). The percentage of bundles remaining was quantified using equation: Fraction bundled (%) = (number of bundles at specific time/total number of bundles at t = 60 s) × 100. Time-dependent distribution of the percentage of remaining actin bundles was fitted by linear regression to obtain the rates of bundles disappearance.

For three-color TIRFM analysis (Fig. S1 D), elongation rates of individual actin bundles were determined by monitoring over many time points a ROI of individual bundles in the actin-phalloidin 647 channel. The actin ROIs were then applied to the fascin (555 nm) and espin (488 nm) channels. A kymograph of all three channels was created from the ROIs using Multi Kymograph - ImageJ plugin (RRID:SCR_003070).

### EM imaging

10 μM G-actin was polymerized for 1 h using the PB buffer. The polymerized actin was incubated with bundling proteins for 1 h at RT to form bundles. These samples were diluted then to 1 µM and applied to glow-discharged, carbon-coated EM grids. After 60 s of adsorption, the grids were blotted dry and treated with 2% uranyl acetate for 45 s at RT. The grids were examined in a FEI Technai T12 electron microscope operated at 120 kV. Actin bundles images were taken at a 26,000× magnification, defocus from −3 to −8 with a Gatan Ultrascan1000 4 MP CCD (2 by 2 k) camera, and Gatan digital micrograph software (RRID:SCR_014492) was used for image acquisition. Actin bundles were selected in a randomized manner across multiple grids squares that were identically fixed and stained, with no bias toward a specific morphology and size. The collected images were analyzed using the Gatan digital micrograph software (RRID:SCR_014492) and ImageJ software (RRID:SCR_003070). Even though we imaged more than five bundles per condition (when using high molar ratio of bundling proteins to actin), not all bundles were suitable for quantitative analysis due to overlapping filaments or disassembled filaments on the top of the bundles. Therefore, the only bundles regions in bundles where individual filaments could be seen to make reliable measurements were considered.

### Correlation analysis

We also used our EM and TIRF assays to look for a correlation between the increase in bundle length and width. In particular, for exact correlation determination of these parameters, the length and width of the same actin bundles is required. However, as mentioned, we found that due to fluorescence intensity saturation (after five actin filaments; Fig. S2 E), the thickness of bundles could not be measured using TIRFM assays (which were used to determine the length of the bundles). Furthermore, EM analysis does not allow us to measure over time the length of the filaments/bundles. Thus, to see if our results at least point to the idea that these two parameters are correlated, we plotted our results using TIRFM assays to determine the length of the bundles and EM assays to determine the width of the bundles and presented our results in Fig. S2 G.

### NADPH consumption assays

To determine whether espin alone or espin-bundled F-actin activates Mical’s catalytic activity, we looked at Mical’s utilization/consumption of its co-enzyme. Unbundled actin filaments activate the catalytic activity of Mical (Hung et al., 2011). Fascin-bundled actin filaments also activate Mical’s enzyme activity, but at a lower rate and extent than unbundled filaments (Rajan et al., 2023c). Therefore, we wondered if actin filaments bundled with espin or fascin + espin could also activate Mical—or if espin’s or fascin + espin bundling of F-actin might prevent/block Mical’s active site binding to or activation by F-actin. NADPH consumption (conversion of NADPH to NADP^+^) by Mical was measured as described by Grintsevich et al. (2016); Hung et al. (2011); Rajan et al. (2023c), with a decrease in the reduced form of NADPH determined from the decreased light absorption at 340 nm. In particular, espin, fascin + espin, or F-actin preincubated with espin or espin + fascin in 1× PB buffer was added to a polymerization buffer containing NADPH and supplemented with Mical. The change in NADPH consumption was calculated by subtracting its consumption in the absence of Mical from that in its presence.

### Fly stocks, molecular biology, and genetics

Fly stocks, including Mical, were the same as previously reported (Hung et al., 2010; Rajan et al., 2023c), except for stocks of *espin*, *fascin*, and *espin* and *fascin* double mutants, which were obtained from the Bloomington *Drosophila* Stock Center, Bloomington, Indiana, USA, or were a kind gift of U. Abdu (Ben-Gurion University of the Negev, Be'er Sheva, Israel). The B11-GAL4 bristle driver was employed for bristle expression, as previously done (Hung et al., 2010; Rajan et al., 2023c). Multiple *espin* (*forked* [*f*]) and *fascin* (*singed* [*sn*]) alleles and their combinations were examined including *f*^*36a*^ (which is a well-characterized null allele; RRID:BDSC_43 [Petersen et al., 1994]) an*d sn*^*3*^ (a well-characterized hypomorphic allele; RRID:BDSC_113 [Cant et al., 1994; Paterson and O'Hare, 1991]).

### *In vivo* analyses of bristle cell remodeling and bundled F-actin organization

Fascin and espin are associated with common F-actin structures, such as filopodia, microvilli, stereocilia, and *Drosophila* bristles (Bartles, 2000; Bartles et al., 1998; Chou et al., 2011; DeRosier and Tilney, 2000; Grieshaber and Petersen, 1999; Grieshaber et al., 2001; Perrin et al., 2013; Petersen et al., 1994; Rajan et al., 2023a; Shin et al., 2010; Shin et al., 2013; Tilney et al., 2004). *Drosophila* bristles, in particular, have become a model for studying these complex F-actin bundles *in vivo* (Tilney et al., 1995; Tilney et al., 1998; Tilney et al., 2000; Tilney and DeRosier, 2005; Wulfkuhle et al., 1998). We therefore used *Drosophila* bristles as a model for studying these complex F-actin bundles *in vivo* and the effects of Mical on them. Analysis of bristle cell remodeling, genetic interactions, and their imaging, drawings, quantification, and statistics were done as described (Hung et al., 2010; Rajan et al., 2023c). In particular, flies were crossed at 25°C and newly emerging adult offspring were collected, genotyped, and analyzed under a dissecting microscope (Leica Stereo Zoom S8 APO). Bristles were then examined for defects in morphology. The number of branches on each scutellar bristle was counted, and the results were presented as the mean number of branches per bristle (± SEM). Data were plotted and statistically analyzed using GraphPad Prism software (RRID:SCR_002798). Adult bristle imaging and drawings were done with the aid of a Zeiss Discovery M2 Bio stereomicroscope using a Zeiss 1.5× PlanApo S objective at RT (22°C) with a motorized focus and zoom, a Zeiss Axiocam HRc camera, three-dimensional reconstruction software (Zeiss Axiovision [RRID:SCR_002677] and Extended Focus [a kind gift from B. Lee (via Zeiss, Oberkochen, Germany)]), and Microsoft Office Powerpoint software (RRID:SCR_023631).

Imaging of *Drosophila* pupae for bundled F-actin was also done as described (Hung et al., 2010; Rajan et al., 2023c). In particular, white prepupae from each genotype were identified with the aid of Tb balancers and were marked to collect. After 44 h of prepupal development, the pupae were collected onto double-sided tape placed in a Petri dish. The pupae were then carefully removed from their pupal cases and positioned dorsal side down on glass-bottom Petri dishes (Part No. P35GC-1.5-14-C; MatTek). Imaging of notal bristles was performed at RT (22°C) using a Zeiss LSM880 confocal microscope with Zen 2.3 software (RRID:SCR_013672; Zeiss) equipped with a Zeiss 20 × Plan-Apochromat objective (0.8 numerical aperture) to visualize actin labeled with GFP. Then, representative images of bristles (in JPEG format) were analyzed using ImageJ software (RRID:SCR_003070). Fluorescent regions were isolated by adjusting the color threshold. The ROI in each bristle was manually selected using the polygon selection tool. Measurement parameters were configured to record area, integrated density, and mean gray value. Fluorescence intensity within each ROI was quantified using the “Measure” function, and the integrated density values were recorded for each genotype. This allowed us to determine the percentage of disrupted bundles for a given genotype as compared with the total bundle area. This also allowed us to determine the area in µm^2^ of bundle disassembly for each analyzed genotype. Data were plotted and statistically analyzed using GraphPad Prism software (RRID:SCR_002798).

The following genotypes were used in Fig. 8: Fig. 8 A: *normal* (*UAS:GFP-actin5C/+, B11-GAL4/+*). Fig. 8 B: Bristle *Mical*^*+++*^ (low) left image (*UAS:GFP-actin5C/+, B11-GAL4/+*, *UAS:Mical/+*). Bristle *Mical*^*+++*^ (low) right image (*B11-GAL4/+*, *UAS:Mical/+*). Fig. 8 C: *espin* (–/– mutant) left image (*espin*^*CRISPRKO*^*/espin*^*CRISPRKO*^*, UAS:GFP-actin5C/+, B11-GAL4/+*). *espin* (–/– mutant) right image (*espin*^*CRISPRKO*^*/espin*^*CRISPRKO*^)*.*Fig. 8 D: Bristle *Mical*^*+++*^*+ espin*^*–/–*^ top image (*espin*^*CRISPRKO*^*/espin*^*CRISPRKO*^*, UAS:GFP-actin5C/+, B11-GAL4/+*, *UAS:Mical/+*). Bristle *Mical*^*+++*^*+ espin*^*–/–*^ Bottom Image (*espin*^*CRISPRKO*^*/espin*^*CRISPRKO*^*, B11-GAL4/+*, *UAS:Mical/+*). Fig. 8 E: Bristle *Mical*^*+++*^ (*UAS:GFP-actin5C/+, B11-GAL4/+*, *UAS:Mical/+*), espin^–/–^ (*espin*^*CRISPRKO*^*/espin*^*CRISPRKO*^*, UAS:GFP-actin5C/+, B11-GAL4/+*), *Mical*^*+++*^*+ espin*^*–/–*^ (*espin*^*CRISPRKO*^*/espin*^*CRISPRKO*^*, UAS:GFP-actin5C/+, B11-GAL4/+*, *UAS:Mical/+*). Fig. 8 F, top graph: Bristle *Mical*^*+++*^ (*UAS:GFP-actin5C/+, B11-GAL4/+*, *UAS:Mical/+*), bristle *Mical*^*+++*^*+* decreased *espin* (+/−) (*f*^*36a*^*/+, UAS:GFP-actin5C/+, B11-GAL4/+*, *UAS:Mical/+*), Bristle *Mical*^*+++*^*+* decreased *fascin* (+/−) (*sn*^*3*^*/+, UAS:GFP-actin5C/+, B11-GAL4/+*, *UAS:Mical/+*). Bristle *Mical*^*+++*^ + decreased *espin* (+/−) and *fascin* (+/−) (*f*^*36a*^, *sn*^*3*^*/+, B11-GAL4/+*, *UAS:Mical/+*). Fig. 8 F, bottom graph: Bristle *Mical*^*+++*^ (*B11-GAL4/+*, *UAS:Mical/+*), Bristle *Mical*^*+++*^*+* decreased *espin* (+/−) (*f*^*36a*^*/+, B11-GAL4/+*, *UAS:Mical/+*), Bristle *Mical*^*+++*^*+* decreased *fascin* (+/−) (*sn*^*3*^*/+, B11-GAL4/+*, *UAS:Mical/+*). Bristle *Mical*^*+++*^ + decreased *espin* (+/−) and *fascin* (+/−) (*f*^*36a*^, *sn*^*3*^*/+, B11-GAL4/+*, *UAS:Mical/+*). Fig. 8 G: Bristle *Mical*^*+++*^*+* decreased *espin* (+/−) left image (*f*^*36a*^*/+, UAS:GFP-actin5C/+, B11-GAL4/+*, *UAS:Mical/+*), Bristle *Mical*^*+++*^*+* decreased *espin* (+/−) right image (*f*^*36a*^*/+, B11-GAL4/+*, *UAS:Mical/+*). Fig. 8 H: Bristle *Mical*^*+++*^*+* decreased *fascin* (+/−) left image (*sn*^*3*^*/+, UAS:GFP-actin5C/+, B11-GAL4/+*, *UAS:Mical/+*). Bristle *Mical*^*+++*^*+* decreased *fascin* (+/−) right image (*sn*^*3*^*/+, B11-GAL4/+*, *UAS:Mical/+*). Fig. 8 I: Bristle *Mical*^*+++*^ + decreased *espin* (+/−) and *fascin* (+/−) top image (*f*^*36a*^*/+*, *sn*^*3*^*/+, UAS:GFP-actin5C/+, B11-GAL4/+*, *UAS:Mical/+*). Bristle *Mical*^*+++*^ + decreased *espin* (+/−) and *fascin* (+/−) bottom image (*f*^*36a*^*/+*, *sn*^*3*^*/+, B11-GAL4/+*, *UAS:Mical/+*).

### Statistics, reproducibility, and additional information

For each representative protein purification, image, gel, immunoblot, graph, or *in vivo* experiment, the experiments were repeated at least two separate independent times, and there were no limitations in repeatability. No statistical method was used to predetermine the sample size, which was based on what is published in the field. Differences between experimental and control animal conditions were highly notable, with relatively little variability, and so the sample size was larger than needed to ensure adequate power analysis to detect an effect. Animal studies complied with the Institutional Animal Care and Use Committee guidelines. Both males and females of *Drosophila* pupae and adults were used. Animal studies were based on pre-established criteria to compare against age-matched animals. Animal experiments were not randomized. Animals of the correct genotype were determined, and those collected of that genotype were included as data. Likewise, for biochemical experiments, samples were grouped together based on experimental conditions and collected data points for those experiments are presented. For all genetic experiments, the genotype needed to be determined based on different genetic/chromosome markers, so blinding was not employed. Likewise, for the biochemical experiments, different proteins and reagents for the particular data set needed to be added and then analyzed using specific technical approaches and expertise, and so blinding was not employed. For both genetic and biochemical experiments, differences between the control and experimental conditions were highly notable and reproducible in both biological and technical replicates. Images and gels show representative examples of experimental results, including that brightness, contrast, sharpness, background, and/or color balance were adjusted uniformly across the whole image using Adobe Photoshop software (RRID:SCR_014199) or Microsoft Office Powerpoint software (RRID:SCR_023631). Graphs show mean ± SEM or the percentage of animals with that defect. For each graph, the value of *n* and what *n* represents are stated in the figure legend. For statistical analysis, the statistical test used and the P value for each comparison are stated in the figure legend. Statistical methods used were based on what is standard in the field, and no statistical tests were used to determine whether the data met assumptions of the statistical approach. All statistical analyses were performed using GraphPad Prism Version #10.5 (RRID:SCR_002798). A P value of P > 0.05 is not considered statistically significant. Single asterisk (*) indicates P < 0.05, double asterisks (**) indicate P < 0.01, triple asterisks (***) indicate P < 0.001, and quadruple asterisks (****) indicate P < 0.0001. To the best of our knowledge, the statistical tests are justified as appropriate.

### Online supplemental material

Fig. S1 shows further analysis of fascin and espin interactions with F-actin. Fig. S2 shows further analysis of the effect of different combinations of bundling proteins on actin bundles formation and topology. Fig. S3 shows further analysis of the Mical’s disassembly effects on different combinations of bundling proteins in actin bundles. Fig. S4 shows further analysis of Mical and cofilin synergy in disassembling fascin, espin, and fascin–espin co-bundled F-actin—and fascin and espin’s combined protection against this disassembly. Fig. S5 shows further analysis of the biochemical mechanism underlying Mical and cofilin’s effects on bundled forms of F-actin. Video 1 shows Mical/NADPH-mediated disassembly of fascin-bundled, espin-bundled, and fascin + espin co-bundled actin filaments. Video 2 shows cofilin-mediated disassembly of fascin-bundled, espin-bundled, and fascin + espin co-bundled actin filaments. Video 3 shows Mical/NADPH and cofilin-mediated disassembly of fascin-bundled, espin-bundled, and fascin + espin co-bundled actin filaments.

## Supplementary Material

Review History

SourceData F2 is the source file for Fig. 2.

SourceData F3 is the source file for Fig. 3.

SourceData F7 is the source file for Fig. 7.

SourceData FS1 is the source file for Fig. S1.

SourceData FS3 is the source file for Fig. S3.

SourceData FS5 is the source file for Fig. S5.

## Data Availability

The data are available in the published article and its online supplemental material and are also available from the corresponding authors upon request.
