# Supplementary figures and images for: Synergistic assembly, disassembly, and protection of complex forms of bundled F-actin
Source: J Cell Biol. 2026 Jul 1;225(8):e202509039. doi: 10.1083/jcb.202509039 (PMC13322139; doi:10.1083/jcb.202509039)

SourceData Fig2A

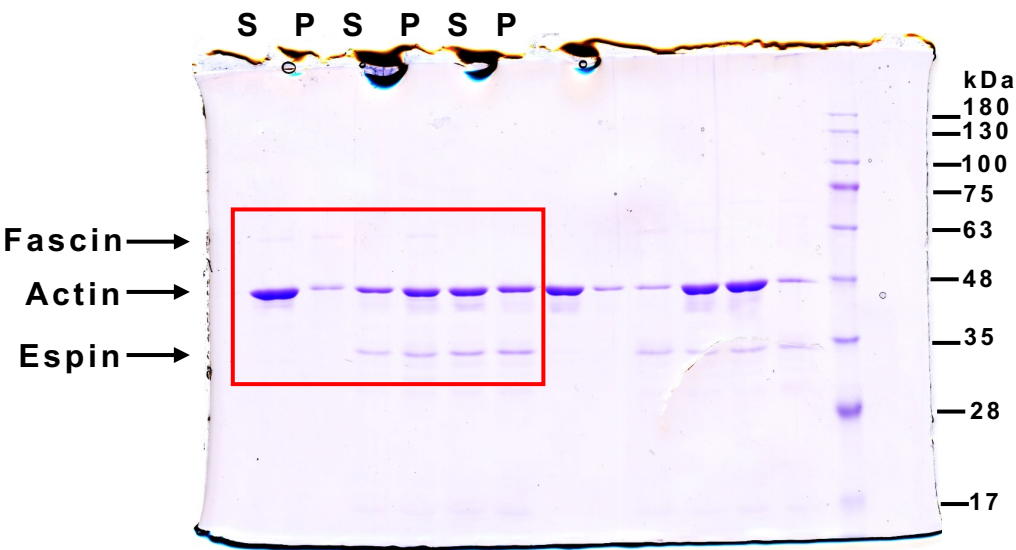

|             |     |     |     |
|-------------|-----|-----|-----|
| Fascin (μM) | 0.1 | 0.1 | 0   |
| Espin (μM)  | 0   | 0.3 | 0.3 |

Supplement: SourceData F2 — is the source file for Fig. 2. [file jcb_202509039_sourcedataf2.pdf]

SourceData Fig 3A-B

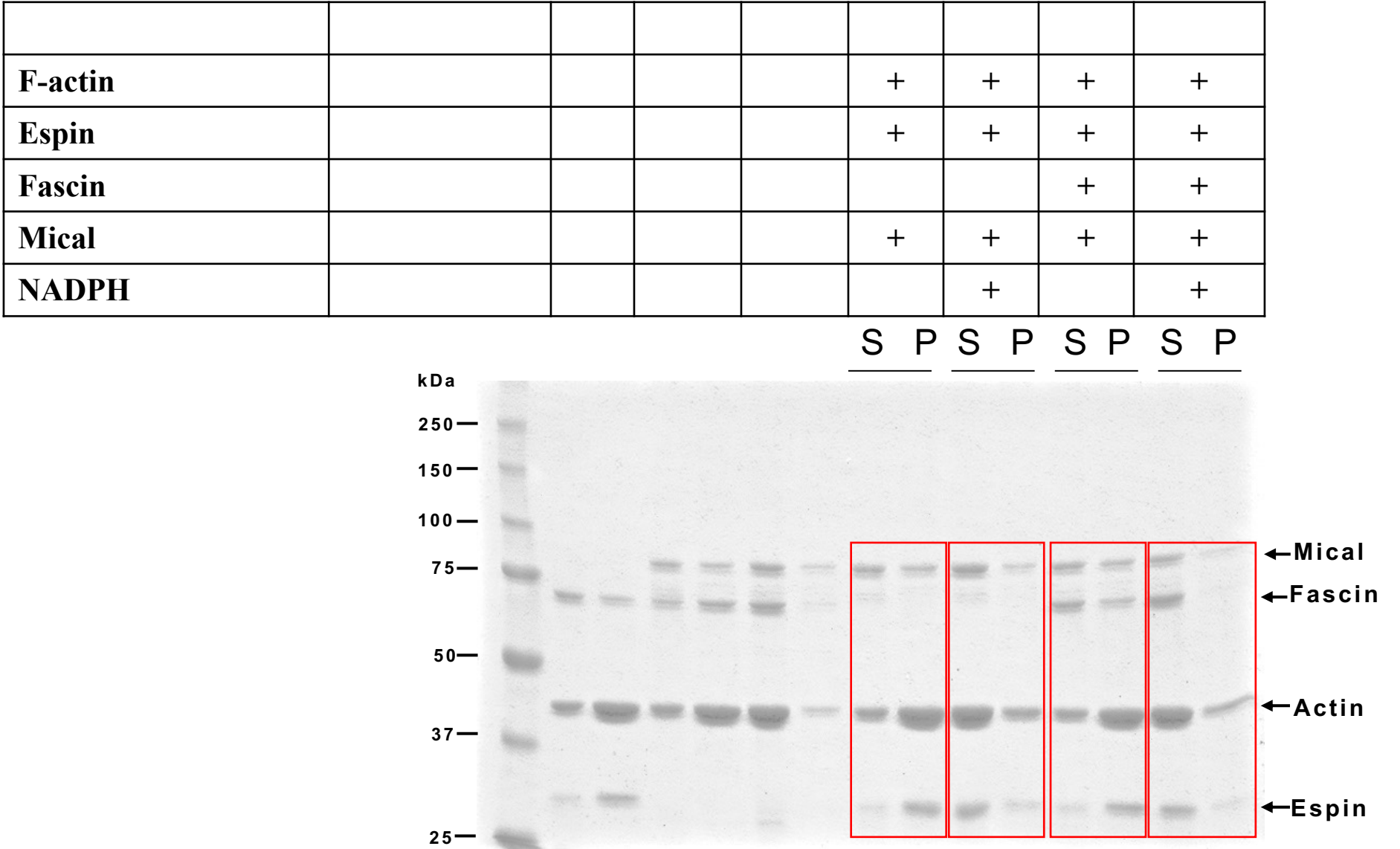

SourceData Fig 3C

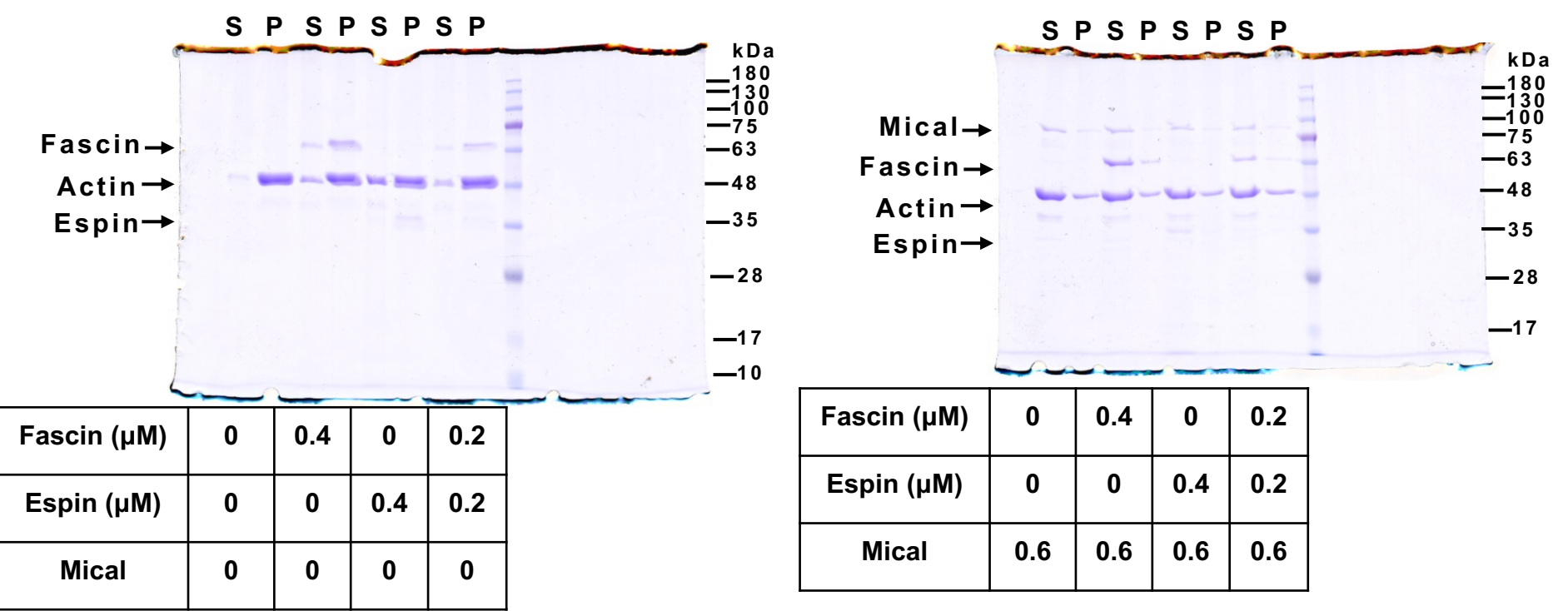

Supplement: SourceData F3 — is the source file for Fig. 3. [file jcb_202509039_sourcedataf3.pdf]

SourceData Fig 7C

Actin

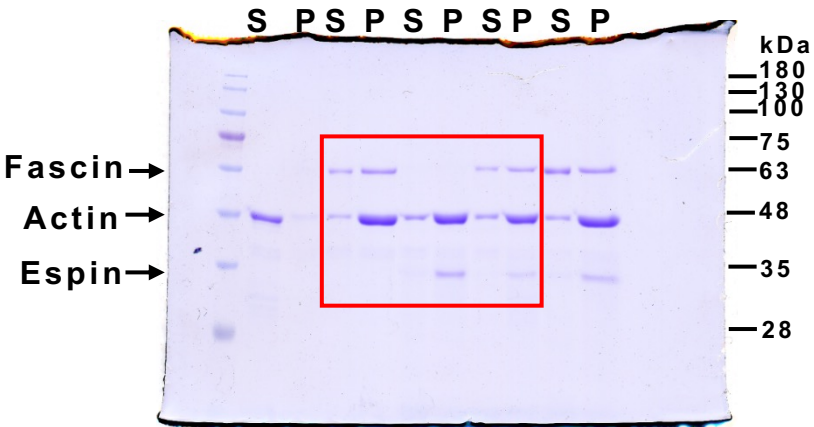

|        |   |   |   |
|--------|---|---|---|
| Fascin | + | - | + |
| Espin  | - | + | + |

Mox-actin

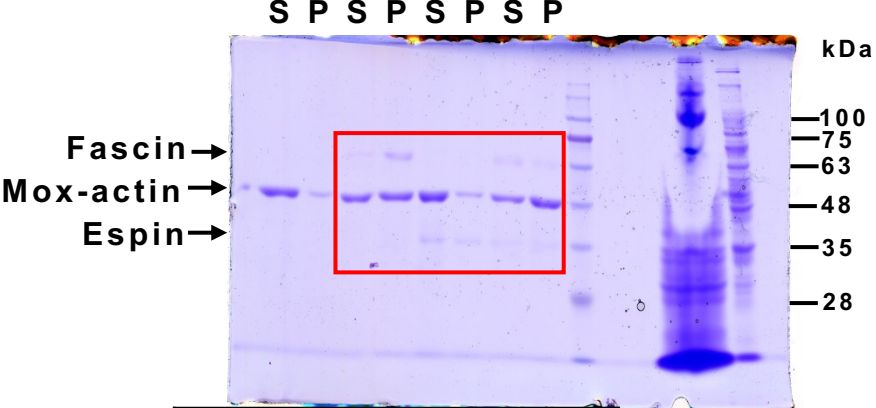

|        |   |   |   |
|--------|---|---|---|
| Fascin | + | - | + |
| Espin  | - | + | + |

Supplement: SourceData F7 — is the source file for Fig. 7. [file jcb_202509039_sourcedataf7.pdf]

SourceData Fig S1A

(1) Ni-NTA agarose column

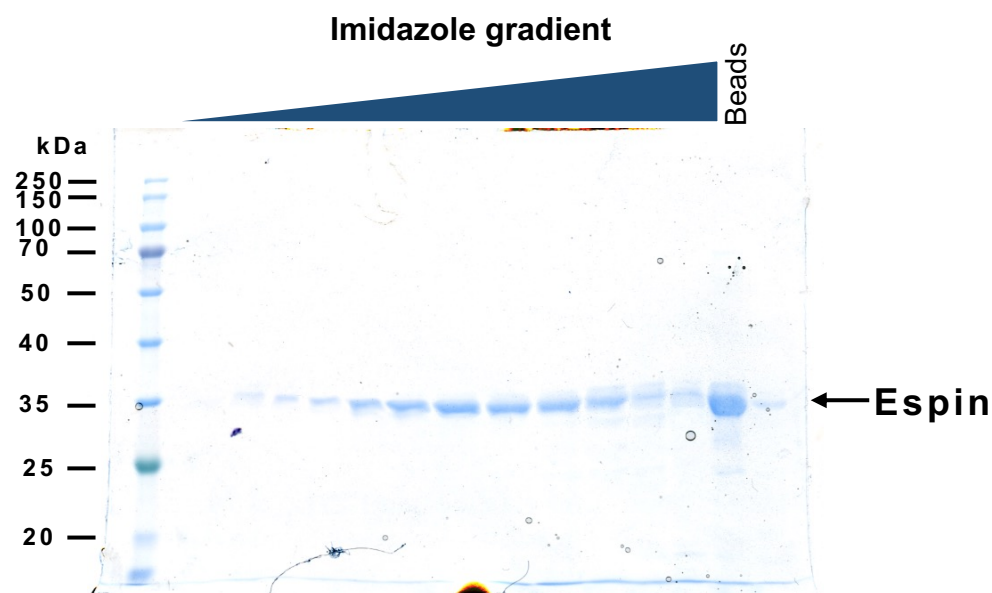

(2) Western Blot

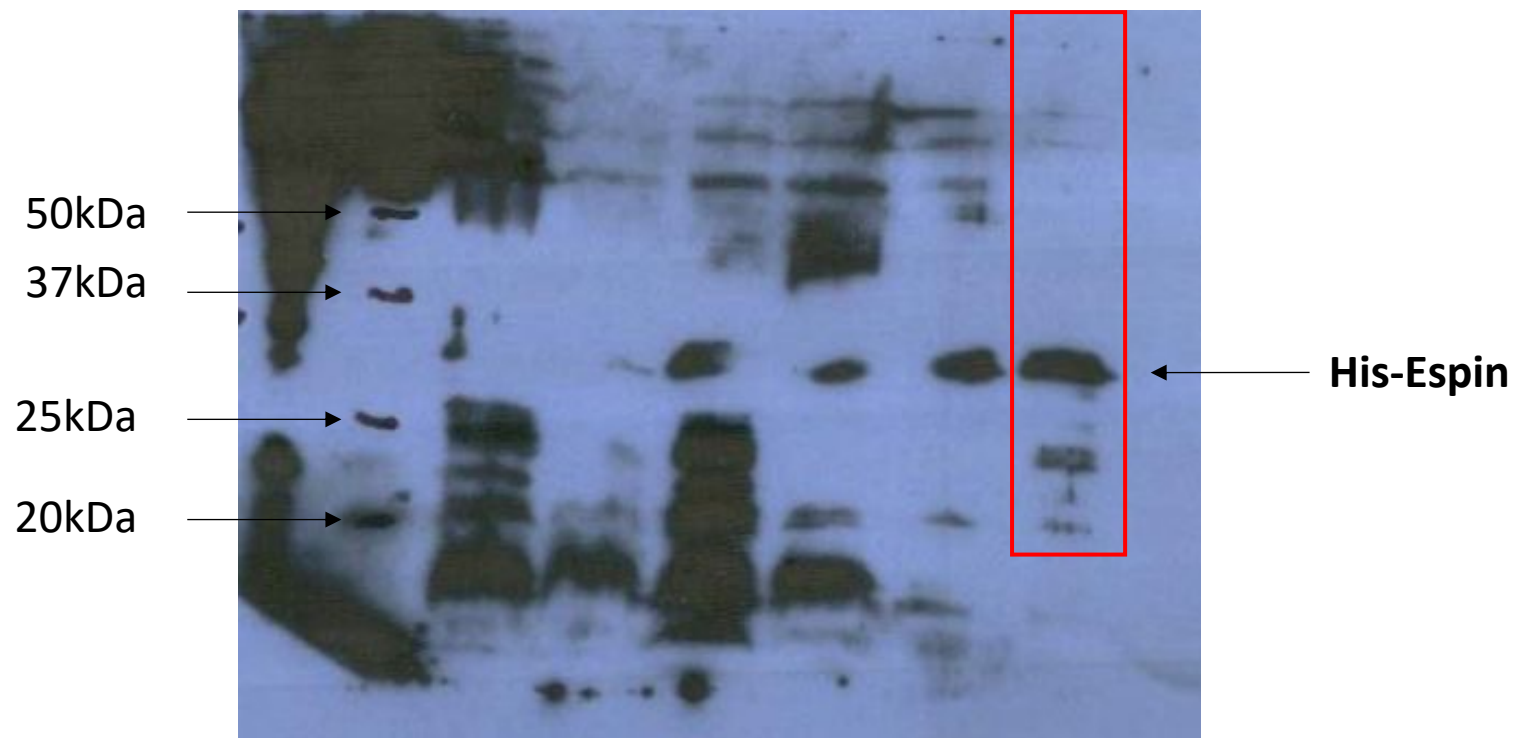

(3) Purified Protein

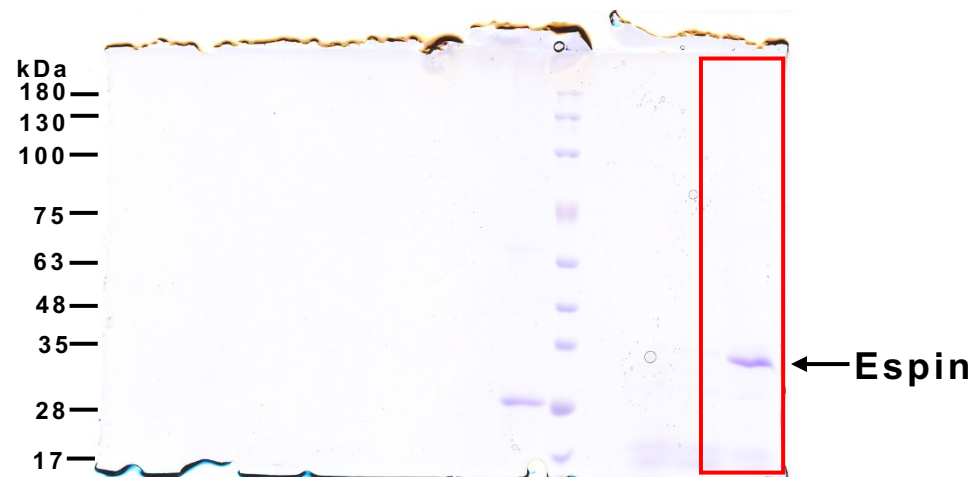

(1)

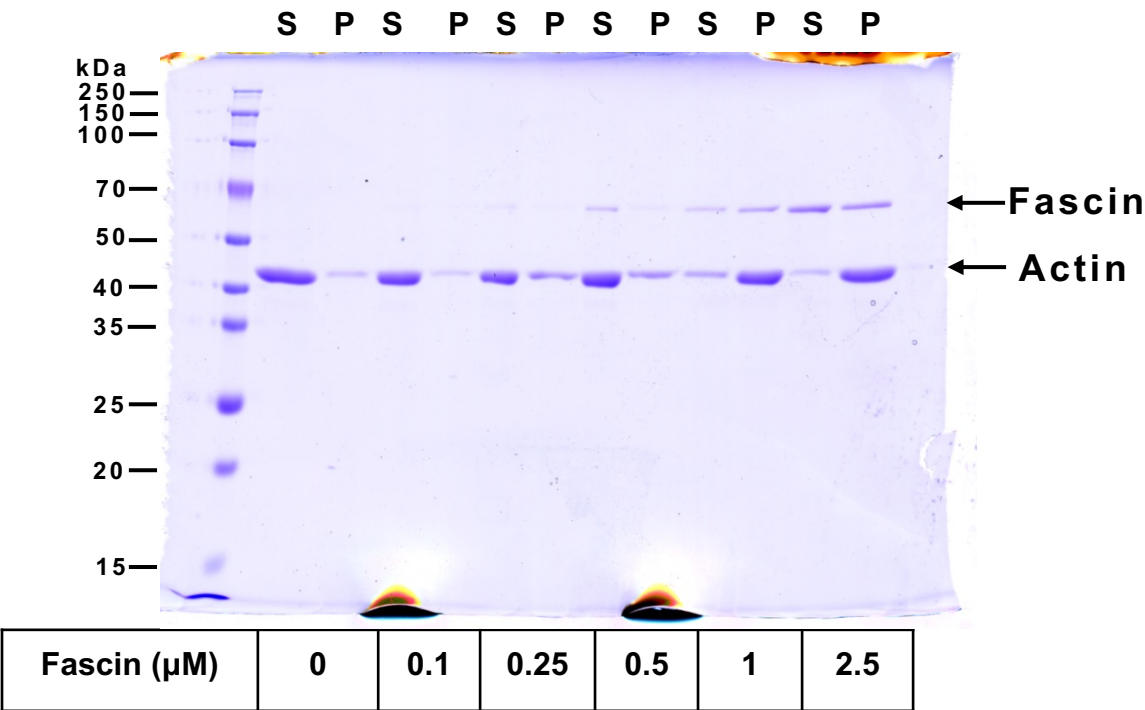

(2)

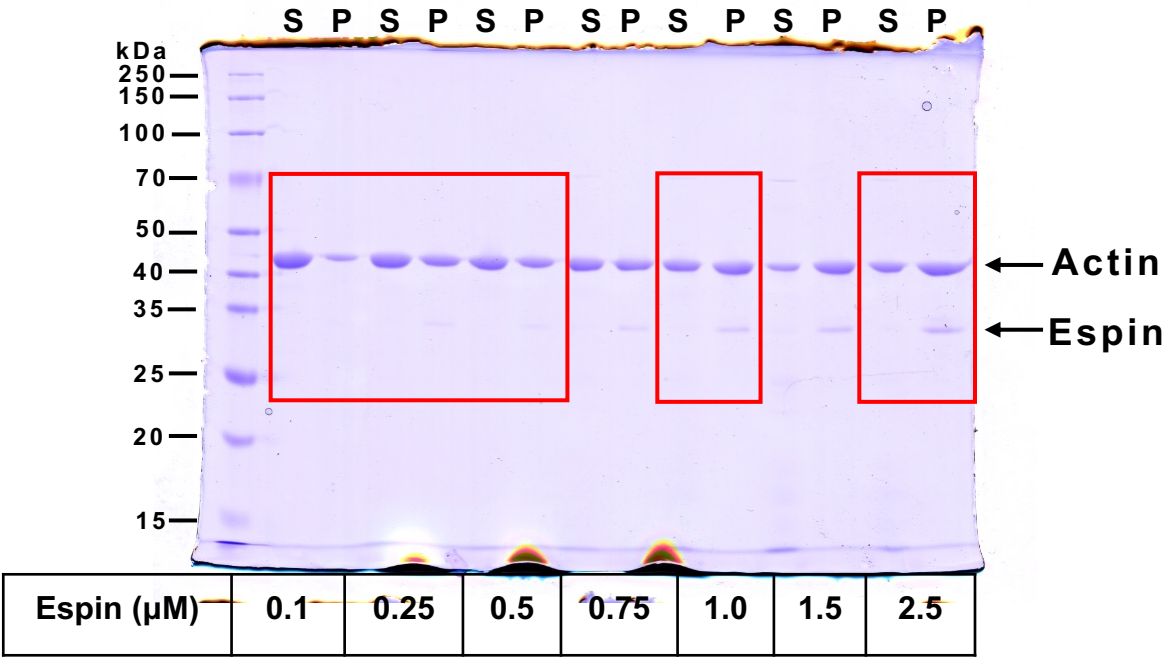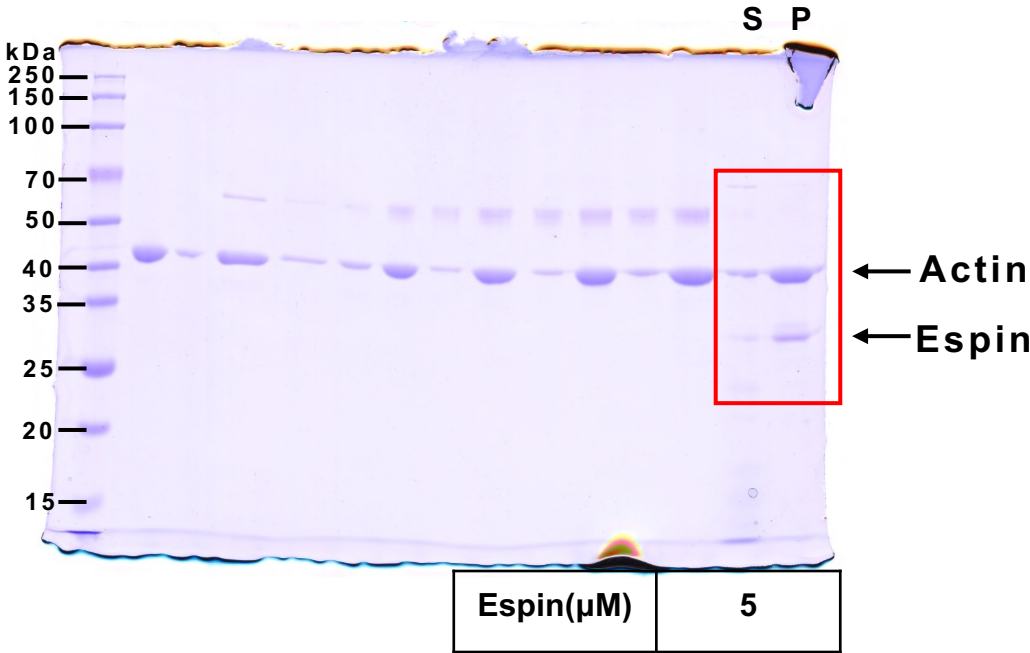

E

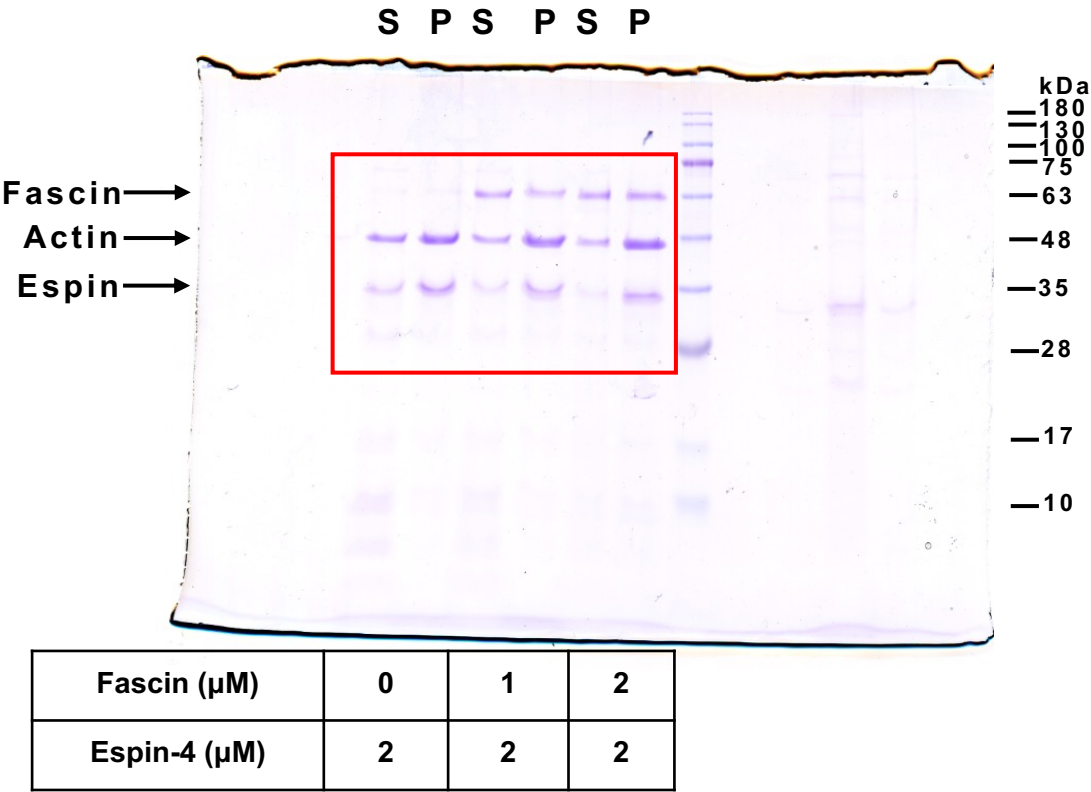

F

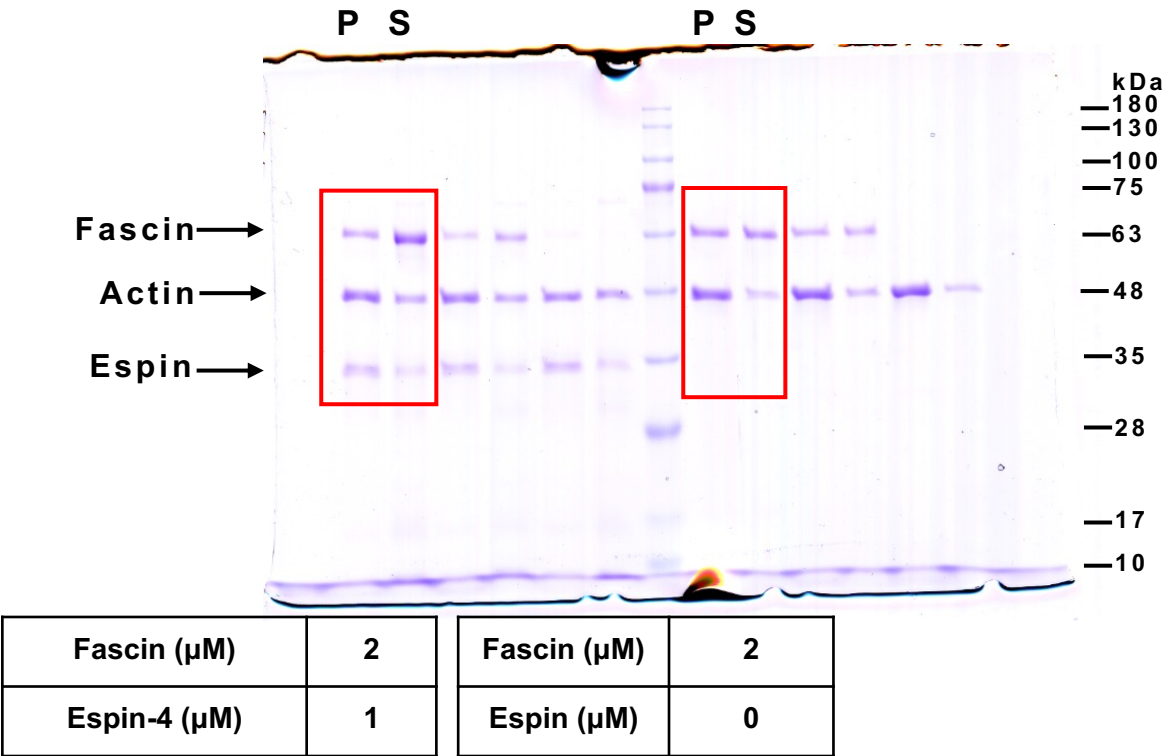

Supplement: SourceData FS1 — is the source file for Fig. S1. [file jcb_202509039_sourcedatafs1.pdf]

Without Mical/NADPH

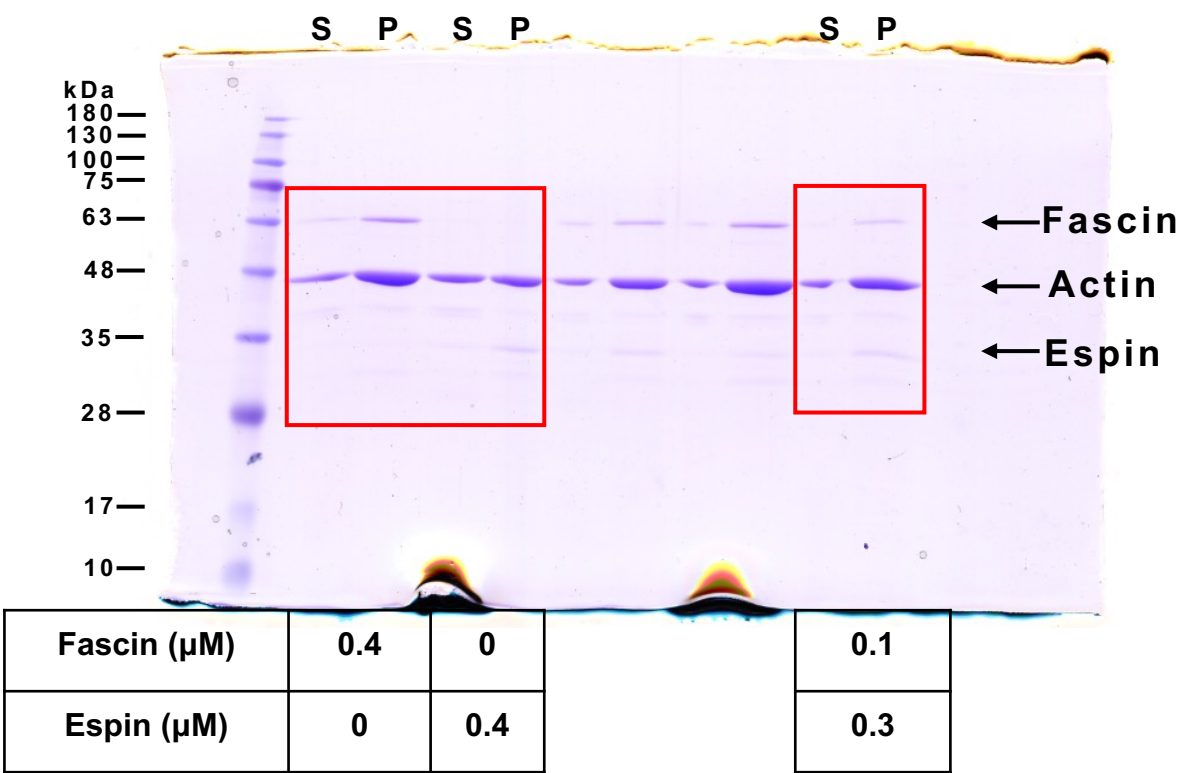

With Mical/NADPH

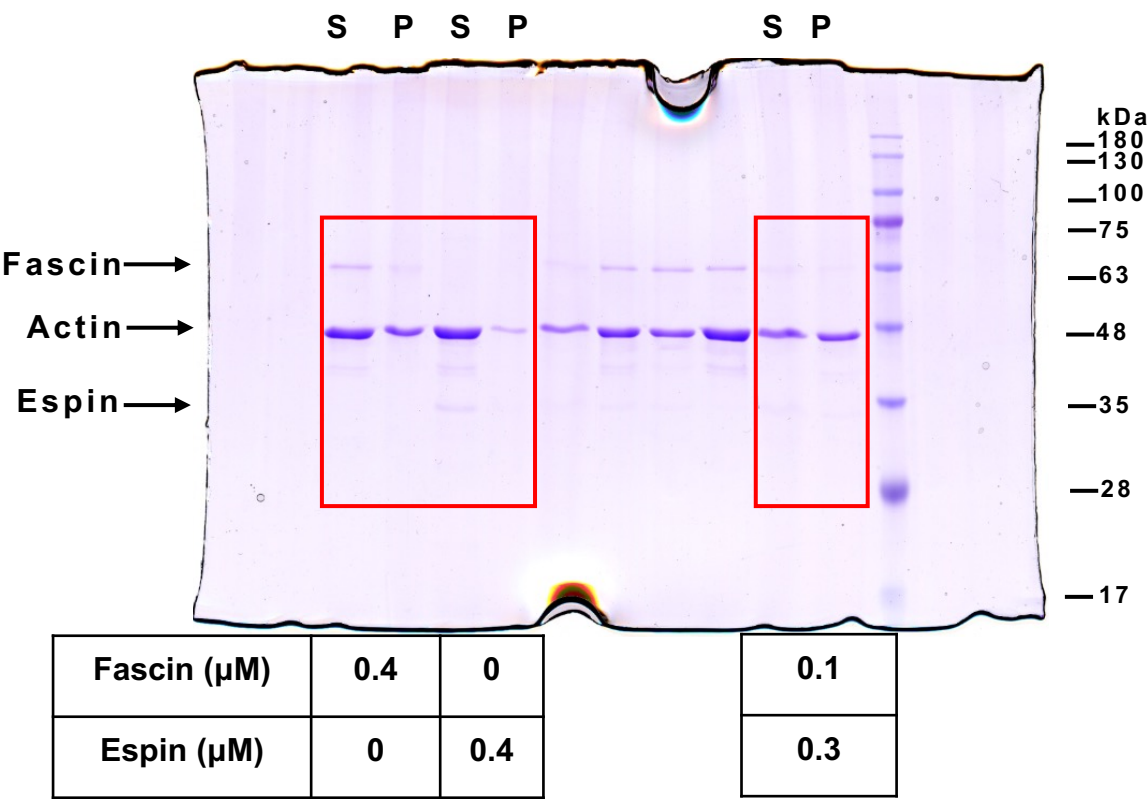

Supplement: SourceData FS3 — is the source file for Fig. S3. [file jcb_202509039_sourcedatafs3.pdf]
