# Supplementary material for: Synergistic assembly, disassembly, and protection of complex forms of bundled F-actin
Source: J Cell Biol. 2026 Jul 1;225(8):e202509039. doi: 10.1083/jcb.202509039 (PMC13322139; doi:10.1083/jcb.202509039)

SourceData Fig S5A-B

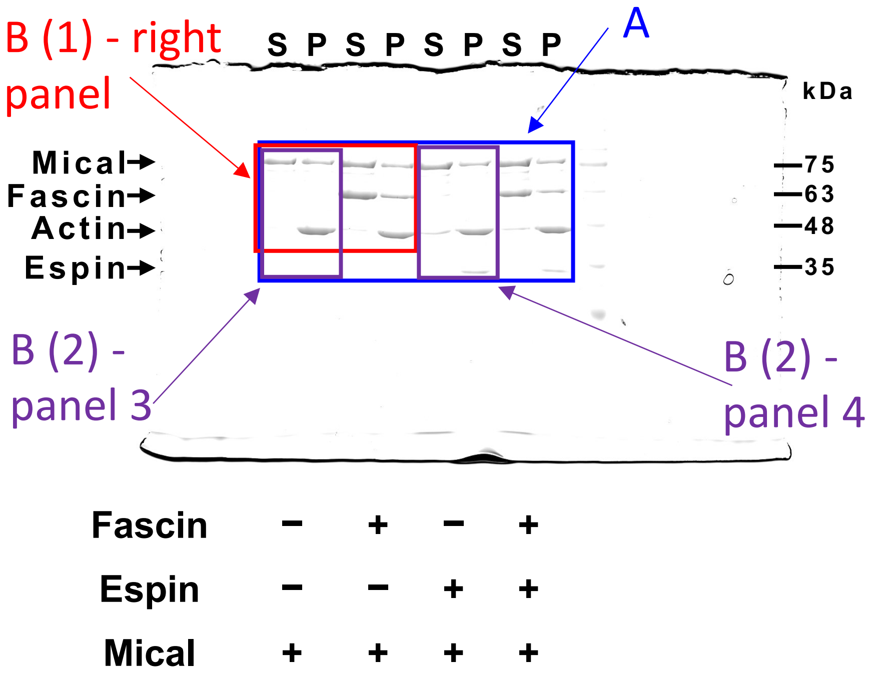

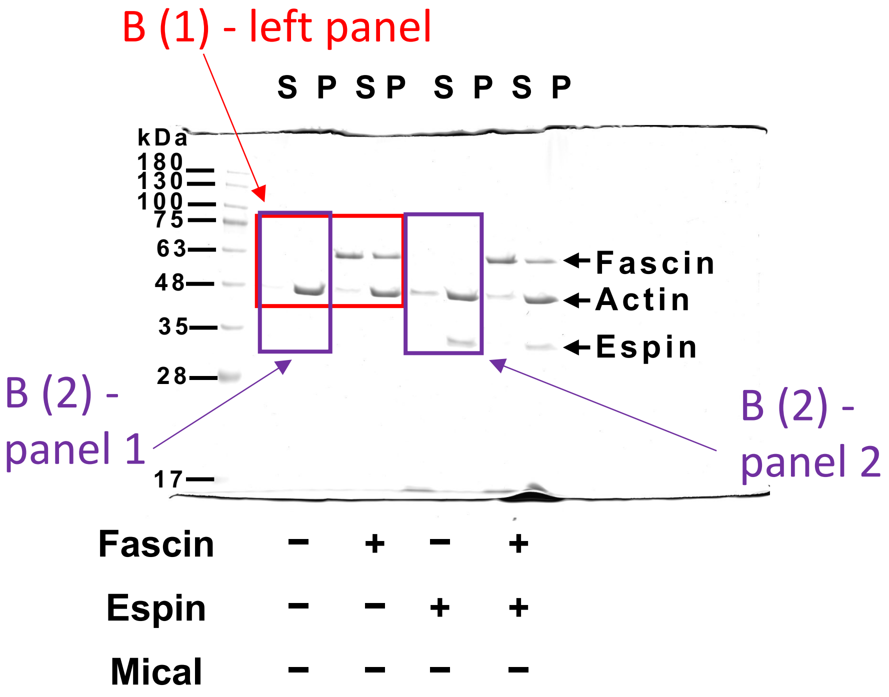

SourceData Fig S5C(1)

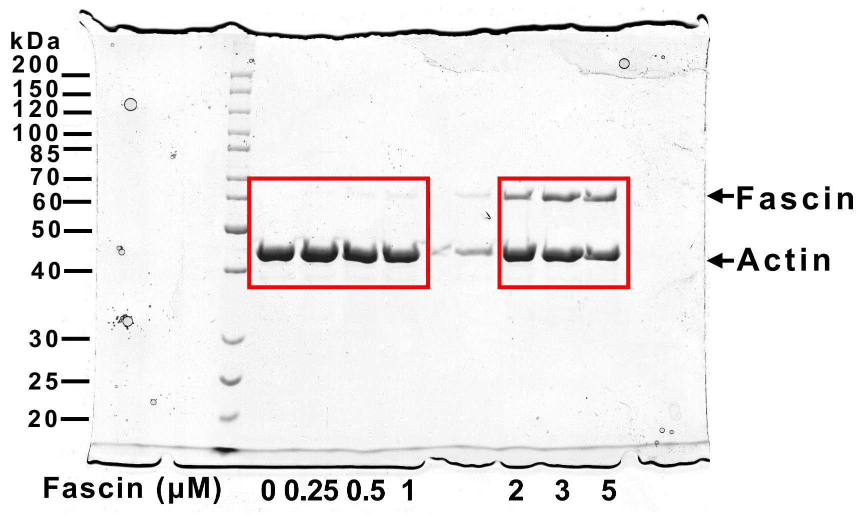

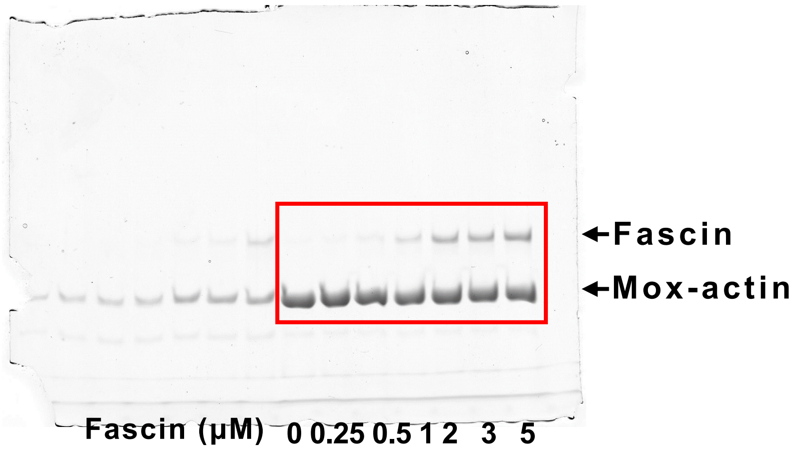

SourceData Fig S5C(2)

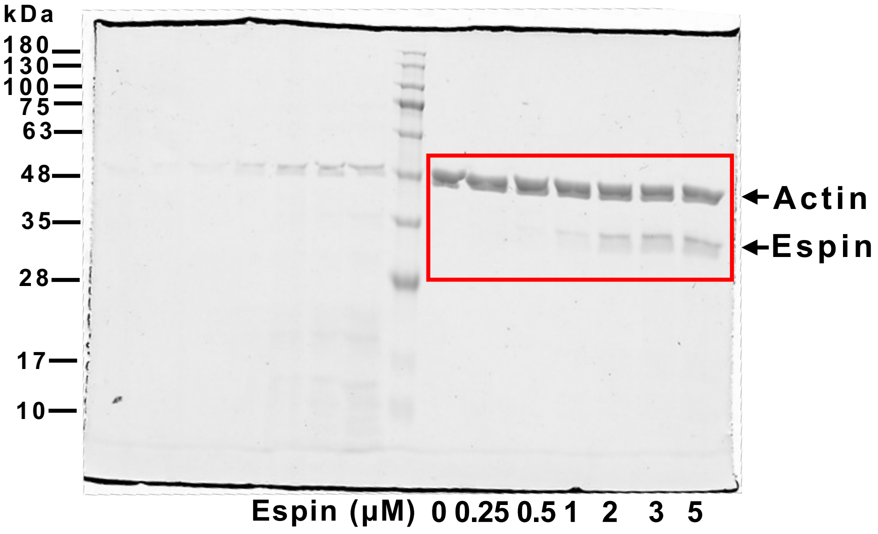

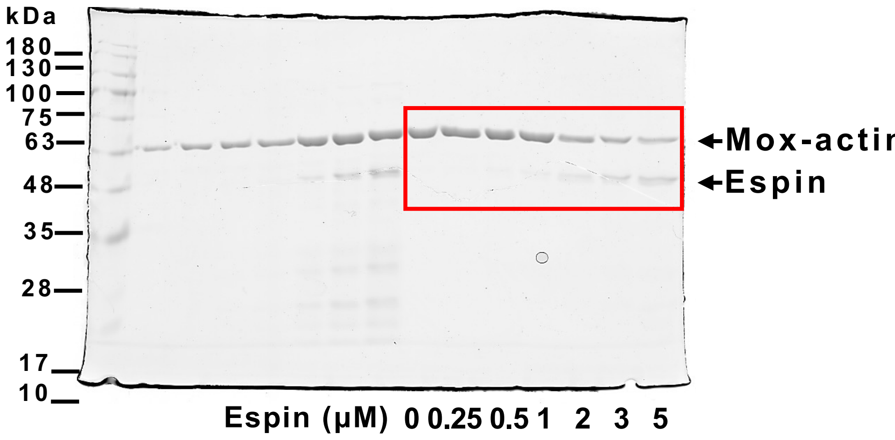

SourceData Fig S5D (1&2)

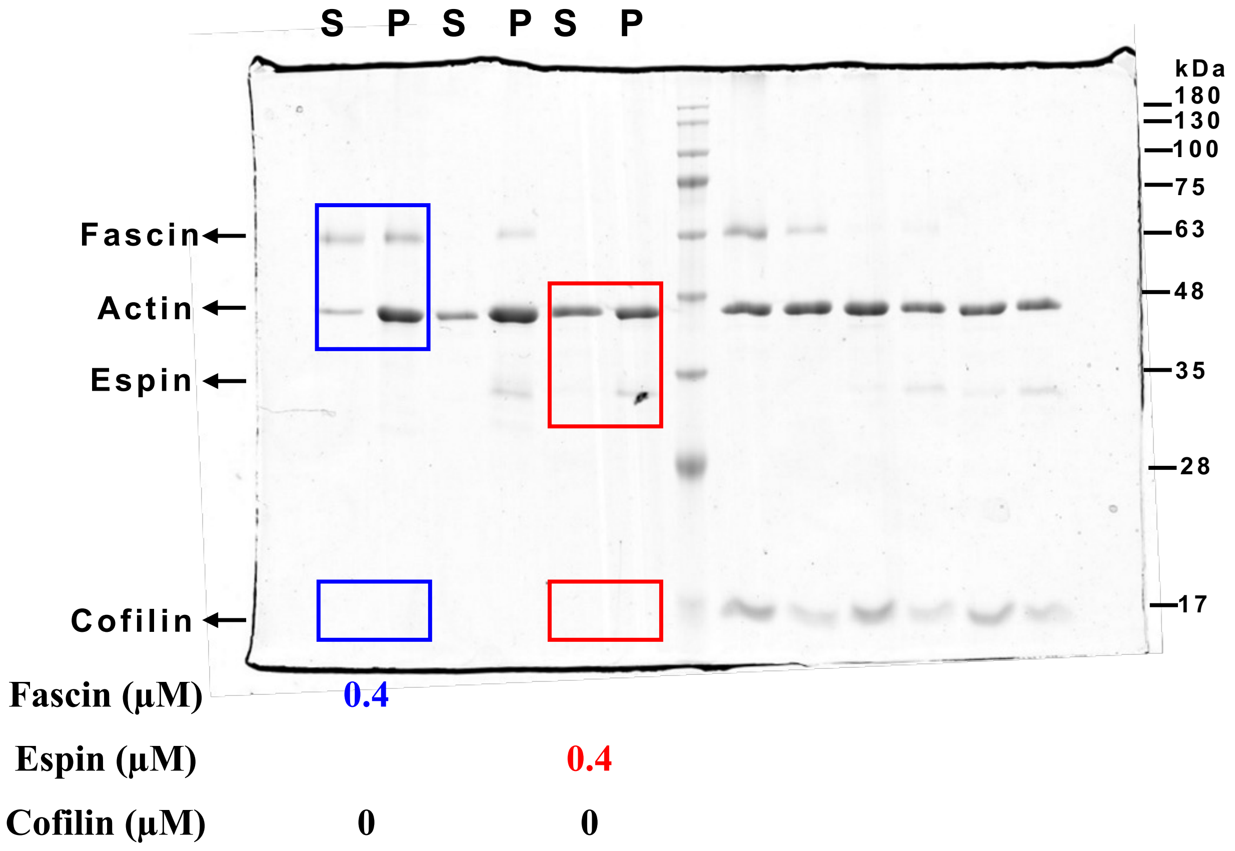

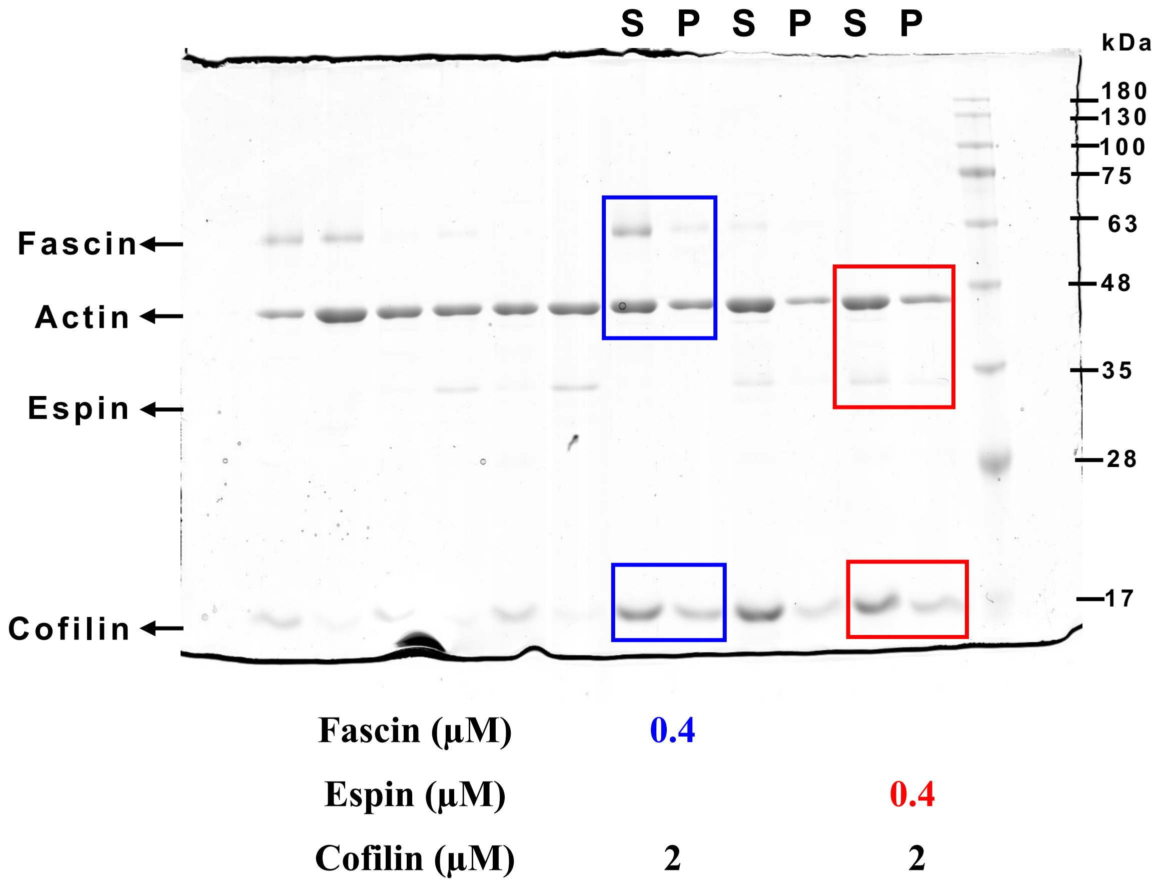

SourceData Fig S5E

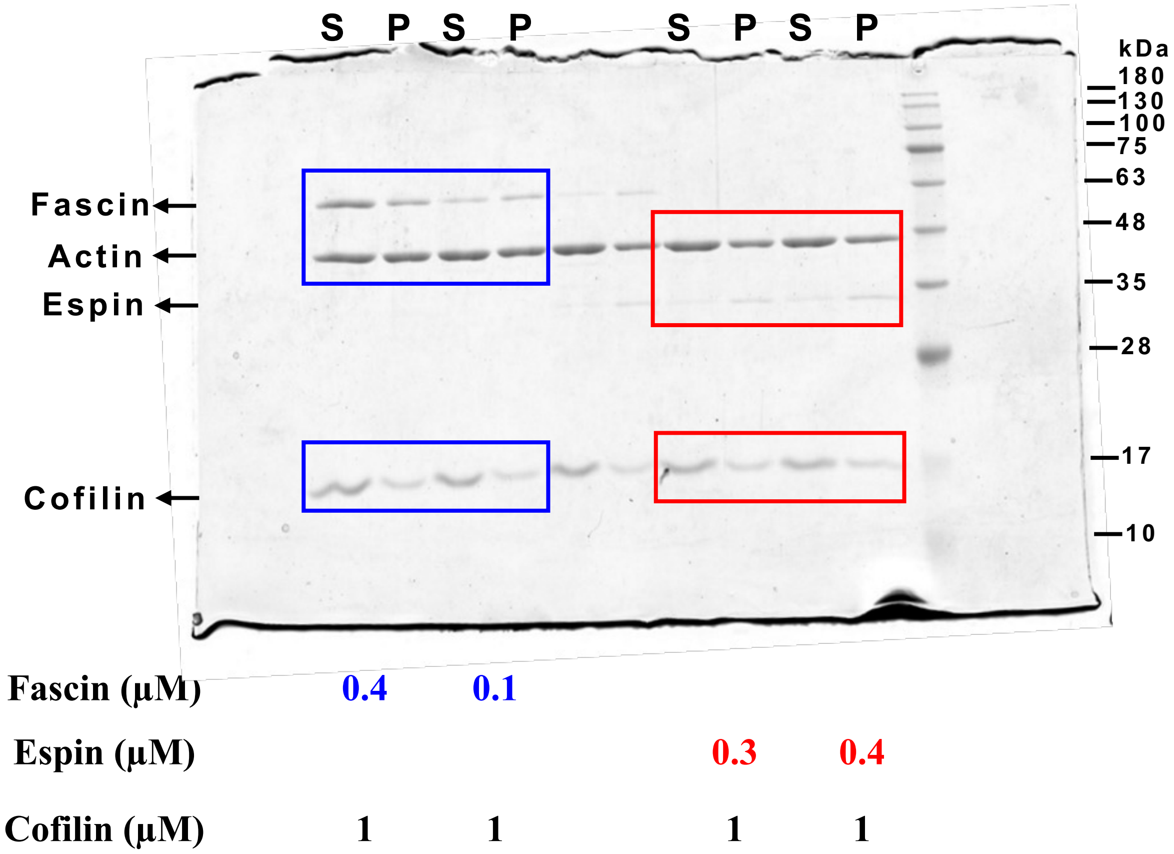

Supplement: SourceData FS5 — is the source file for Fig. S5. [file jcb_202509039_sourcedatafs5.pdf]
