# Supplementary material for: Synergistic assembly, disassembly, and protection of complex forms of bundled F-actin
Source: J Cell Biol. 2026 Jul 1;225(8):e202509039. doi: 10.1083/jcb.202509039 (PMC13322139; doi:10.1083/jcb.202509039)
Supplement: Review History [file jcb_202509039_review_history.pdf]

JCB

Synergistic Assembly, Disassembly, and Protection of Complex Forms of Bundled F-actin

Sudeepa Rajan, Jimok Yoon, Heng Wu, Raju Baskar, Roman Aguirre, Jonathan Terman, and Emil Reisler

Corresponding Author(s): Jonathan Terman, The University of Texas Southwestern Medical Center and Emil Reisler, University of California, Los Angeles

Review Timeline:

Submission Date:	2025-09-05
Editorial Decision:	2025-10-07
Revision Received:	2026-02-28
Editorial Decision:	2026-03-24
Revision Received:	2026-04-30

Monitoring Editor: Pekka Lappalainen

Scientific Editor: Gabriele Stephan

Transaction Report:

(Note: With the exception of the correction of typographical or spelling errors that could be a source of ambiguity, letters and reports are not edited. The original formatting of letters and referee reports may not be reflected in this compilation.)

DOI: https://doi.org/10.1083/jcb.202509039

October 7, 2025

Re: JCB manuscript #202509039

Jonathan Terman
The University of Texas Southwestern Medical Center

Dear Dr. Terman,

Thank you for submitting your manuscript entitled "Synergistic assembly, disassembly, and protection of complex forms of bundled F-actin". Your manuscript has been assessed by expert reviewers, whose comments are appended below. Although the reviewers express potential interest in this work, significant concerns unfortunately preclude publication of the current version of the manuscript in JCB.

While all reviewers think the paper is timely and presents important findings towards actin filament bundle formation and turnover, and while reviewers #1 and #2 list mostly relatively minor concerns, reviewer #3 voices more substantial critiques. We think your paper could improve if you were able to address all of the reviewers' comments, with a particular focus on the mechanistic extensions requested by reviewer #3. This includes providing mechanistic insights into how espin and fascin (individually and together with each other) stabilize actin filament bundles against cofilin and MICAL-mediated disassembly. Additional explanations requested by reviewer #1 as well as experimental optimizations and clarifications as suggested by reviewer #2 should further be included in a revised manuscript.

Please let us know if you are able to address the major issues outlined above and wish to submit a revised manuscript to JCB. Note that a substantial amount of additional experimental data likely would be needed to satisfactorily address the concerns of the reviewers. The typical timeframe for revisions is three to four months. If you anticipate any difficulties in meeting this aforementioned revision time limit, please contact us and we can work with you to find an appropriate time frame for resubmission.

Prior to resubmission, please provide a detailed revision plan, outlining how to plan to address the reviewers' concerns.

Please note that papers are generally considered through only one revision cycle, so any revised manuscript will likely be either accepted or rejected.

If you choose to revise and resubmit your manuscript, please also attend to the following editorial points. Please direct any editorial questions to the journal office

GENERAL GUIDELINES:
Text limits: Character count is < 40,000, not including spaces. Count includes title page, abstract, introduction, results, discussion, and acknowledgments. Count does not include materials and methods, figure legends, references, tables, or supplemental legends.

Figures: Your manuscript may have up to 10 main text figures. To avoid delays in production, figures must be prepared according to the policies outlined in our Instructions to Authors, under Data Presentation, https://jcb.rupress.org/site/misc/ifora.xhtml. All figures in accepted manuscripts will be screened prior to publication.

IMPORTANT: It is JCB policy that if requested, original data images must be made available. Failure to provide original images upon request will result in unavoidable delays in publication. Please ensure that you have access to all original microscopy and blot data images before submitting your revision.

Supplemental information: There are strict limits on the allowable amount of supplemental data. Your manuscript may have up to 5 supplemental figures. Up to 10 supplemental videos or flash animations are allowed. A summary of all supplemental material should appear at the end of the Materials and methods section.

Please note that JCB now requires authors to submit Source Data used to generate figures containing gels and Western blots with all revised manuscripts. This Source Data consists of fully uncropped and unprocessed images for each gel/blot displayed in the main and supplemental figures. For assays performed using capillary electrophoresis and/or immunoassay-based detection, authors should instead provide the electropherogram graph(s) for each experiment, plotting fluorescence/chemiluminescence intensity vs. molecular weight/size. Please be sure to provide one Source Data file for each figure gels, blots, and/or capillary electrophoresis assays along with your revised manuscript files. File names for Source Data figures should be alphanumeric without any spaces or special characters (i.e., SourceDataF#, where F# refers to the associated main figure number or SourceDataFS# for those associated with Supplementary figures). For traditional gels and blots, the lanes of the gels/blots should be labeled as they are in the associated figure, the place where cropping was applied should be

marked (with a box), and molecular weight/size standards should be labeled wherever possible. For capillary electrophoresis assays, each trace in the graph should be color-coded and labeled to indicate which protein, gene, or sample is being measured (please try to avoid red/green combinations to accommodate our color-blind readers).

Source Data files will be made available to reviewers during evaluation of revised manuscripts and, if your paper is eventually published in JCB, the files will be directly linked to specific figures in the published article.

Source Data Figures should be provided as individual PDF files (one file per figure). Authors should endeavor to retain a minimum resolution of 300 dpi or pixels per inch. Please review our instructions for export from Photoshop, Illustrator, and PowerPoint here: https://rupress.org/jcb/pages/submission-guidelines#revised

If you choose to resubmit, please include a cover letter addressing the reviewers' comments point by point. Please also highlight all changes in the text of the manuscript.

Regardless of how you choose to proceed, we hope that the comments below will prove constructive as your work progresses. We would be happy to discuss them further once you've had a chance to consider the points raised. You can contact the journal office with any questions at cellbio@rockefeller.edu.

Thank you for thinking of JCB as an appropriate place to publish your work.

Sincerely,

Pekka Lappalainen
Monitoring Editor
Journal of Cell Biology

Gabriele Stephan, PhD
Scientific Editor
Journal of Cell Biology

Reviewer #1:

This manuscript by Rajan and colleagues investigates the combined effect of two actin bundling proteins, fascin and espin, on the formation of actin filament bundles and on their ability to restist disassembly when exposed to the redox enzyme MICAL1 and/or the filament severing protein cofilin. The work is mostly done in vitro using purified proteins, complemented with a series of experiments on Drosophila bristles.

This is an interesting and timely question, as there is more and more evidence that the way actin filaments are connected together will affect their targeting by regulatory proteins, and because there is still very little data on the combined action of different bundlers. Overall, the work in this manuscript is well done and well presented. However, I think the following points need clarification before it can be published.

1 - Several aspects of the data analysis and quantification are unclear.

For example, is the rate of bundle shortening determined using a population of randomly picked bundles, or only the bundles that were observed to shorten? How were the regions of interests (for a thinning event, for example) chosen? For severing events, are all the bundles candidate sites, or only above a certain intensity or size? Does the bundle length and thickness remain constant during the 60 seconds during which severing is monitored? When a severing event occurs, can the fragments continue to be monitored or do they diffuse away?

On page 25, the explanations for the way the percentage of remaining bundles was computed is unclear. Were fragments counted as gone when they became shorter than 7 µm?

In the legend of Figure 3, the authors explain that bundle thinning rates are determined by an exponential fit as shown in Fig S4D, but in Fig S4D the fits are not shown and the data do not look like exponentials. Looking in the Methods, things get more confusing because the data is said to be "fitted by a linear regression" (page 24). This should be clarified. What would make more sense, a linear decrease or an exponential decrease?
Also confusing, though it is more of a detail, the arrowheads in Fig 3F (and Fig 4B) suggest that thinning and shortening (and severing, in 4B) are already visible at t=0. I assume this is not the case.

2 - I find the measured ~0.6 fascin/actin (Fig S2C) hard to understand. Recent structural data from the Alushin lab (Gong et al.,

Nat SMB 2025, not cited) reports a spacing of 13 actin subunits between fascins that bridge two neighboring filaments. With 6 neighbor filaments (hexagonal lattice) that would mean a maximum fascin:actin ratio of 1/13*6/2=0.23 (the last division by 2 is because each fascin is shared by 2 filaments). This is an overstimate because it neglects the fact that filaments at the bundle periphery will not have as many fascins. Regardless of this last point, the fascin:actin ratio reported here is far above the expected maximum. Note that, in contrast with Fig S2C, Fig 1C seems to indicate a saturation of fascin at a ratio below 0.25 ("bound fascin/actin"). All this should be explained, or at least discussed.

3 - Information on the proteins is missing. The methods indicate (page 20) that fascin and Mical have been purified "as previousy described", but it would be nice to know at least what orthologs were used. Cofilin is said to be human, but which isoform is it? I assume it is cofilin-1, but it would be worth saying so explicitly. On page 4 : "Espins comprise four isoforms" in which species? The in vitro work here is done with espin-4 which is a somewhat peculiar (short) version of espin. This specificity is worth mentioning, and it should be discussed when comparing with published observations on other espins (in the discussion section).

Minor

- Page 21. It is unclear to me why polymerization is done in 10x buffer instead of 1x buffer.

- statistics for EM (Fig 1F). The table indicates that "5 or more bundles" were analyzed. This is a rather low number. How were these bundles chosen? Randomly?

- in Fig 3A, the red circle could be misunderstood as representing MICAL (in spite of the legend, which is a bit ambiguous).

Reviewer #2:

The actin cytoskeleton is essential for diverse cellular processes, including motility, polarity, cell division, and endocytosis. Its dynamic regulation depends on the rapid assembly and disassembly of actin filaments, mediated by numerous actin-binding proteins (ABPs). To orchestrate specific cellular functions at the microscale, actin filaments must be organized into higher-order networks with distinct subcellular architectures and compositions. This spatial organization is established through the coordinated action of crosslinking proteins at defined cellular locations. These structures undergo continuous remodeling to enable cells to adapt to changing environment. While the mechanisms of actin filament network assembly have been extensively characterized, the processes governing their disassembly remain comparatively less understood.

This manuscript investigates the dynamics of assembly and disassembly of parallel actin filament bundles organized by the crosslinking proteins fascin and espin. Through both in vitro and in vivo analyses, the authors demonstrate that fascin and espin act synergistically to generate larger and longer filament bundles. They further show that, although the disassembly factors Mical and cofilin can dismantle bundles crosslinked by fascin and espin, these bundles exhibit increased resistance to Mical- and cofilin-mediated disassembly. This study provide important insights into the dynamic assembly and disassembly process of higher-order actin structures crosslinked by fascin and espin.

Overall, the experiments are well-designed and the results are clearly presented. This study makes an important contribution to our understanding of the remodeling and plasticity of filament bundles crosslinked by multiple crosslinkers. Below are a few specific comments and minor points which I believe should be addressed prior to acceptance for publication:

1. The fascin-F-actin interface and the inter-filament distance have been precisely defined by recent studies (PMID:39833469, 40403900), Thus, the statement on page 5 citing "three putative actin-binding sites" from earlier studies is inaccurate and should be updated.

2. Espin in this study was purified by affinity chromatography without further polishing steps such as ion exchange or size exclusion. As shown in Supplementary Figure 1A-2, the flowthrough after tag removal contains substantial amounts of contaminating proteins. In S1A-3, the contaminating bands below purified espin are not shown. Consequently, the effective concentration of espin used in this study may be considerably lower than measured, which potentially affect all concentration-dependent results. For instance, espin crosslinks F-actin into hexagonal bundles with a saturating stoichiometry of approximately 1:4.33, yet the bundles in Figures 1E and S3F appear extremely thin, even at saturating or near-saturating espin. In contrast, much thicker bundles were observed under similar conditions at lower espin ratio (1 espin per 7 actin subunits, PMID: 9763424). Please clarify the possible reasons for this discrepancy.

3. Since fascin and espin synergistically increase both bundle length and width, please provide data or analysis demonstrating whether these two parameters are correlated.

4. Figure 3 focuses on Mical mediated bundle disassembly by tracking changes in bundle integrity, but it does not reveal the nature of the disassembly products. After disassembly, do the filaments remain as single filament, or are they further converted

into G-actin? Such information would provide important insight into the dynamics of bundle disassembly by mical. At minimum, include high-speed co-sedimentation assay to track whether the disassembled bundles are all converted into G-actin. Related to this, the model in figure 3A shows mical disassembles the filament into G-actin, whereas the model in supplementary 6A suggests that mical oxidized filaments remain intact, which is further disassembled into G-actin by cofilin. Tracking the disassembly products would enable a more detailed and consistent mechanistic model.

5. Do espin-bundled filaments resist Mical-mediated disassembly relative to unbundled F-actin? If so, how do they stimulate Mical activity at a similar rate (Fig. 2B)? Please reconcile these observations.

6. In figure 3E, the green curve declines gradually, consistent with Mical-driven disassembly of fascin-espin-4 bundle. However, in the similar experiment shown in figure 4A-2, the green curve first decreases but then rises sharply to a level comparable to the starting point. This pattern is inconsistent and difficult to interpret, and the blue curve in this panel shows a similar trend. Moreover, in the first panel the green endpoint exceeds the starting value, suggesting increased bundling in the presence of cofilin. Please clarify these discrepancies or optimize the experiments to obtain more consistent results.

Minor points:

1. Espin and espin-4 are interchangeably used throughout the figures and captions, the terminology should be unified for consistency.

2. In figure 3B-1, I assume the Mical only and Mical + Espin-4 curves are overlapping. However, the Mical only curve is not discernable. Please adjust styling to ensure both curves are clearly distinguishable.

3. In figure 4B-2, the espin concentration is labeled as 0.2mM but should be 0.2 uM, please correct this.

Reviewer #3:

The authors are examining how two actin bundling proteins, espin and fascin, work together to build complex filament bundles and how their combined activities control bundle stability in the presence of MICAL and cofilin.

The research traces its historical roots back to pioneering work by Lew Tilney and colleagues who investigated actin-bundle dependent bristle formation in Drosophila. Those early studies led to a model in which bristle formation depended on the sequential action of two bundling proteins, forked and fascin. Forked acts first to initiate the formation of loose bundles that are subsequently zippered together to form the packed bundles inside the bristle. Bristle formation emerged as a useful model for describing other bundled structures like stereocilia which are formed using related bundling proteins espin and fascin-2. Subsequent work revealed additional complexity layered upon this basic model, including critical roles for actin remodeling through cofilin and MICAL that can destabilize filaments and bundles through severing and depolymerization. The importance of espin, fascin, cofilin, and MICAL for bundle formation is vivo is clear. Underlying mechanisms are areas of active research. This manuscript is divided into two sections. The first half focuses on bundle formation to show that espin plus fascin are more effective at bundling than either alone. The second half of the manuscript focuses on bundle stability to show that bundles built through a combination of espin and fascin are more resistant to MICAL and cofilin disassembly than bundles built from either factor alone. Assembly, maintenance, and turnover of filament bundles are important question in the field, and in vitro reconstitution with confirmation of principle in vivo is an excellent approach. However, that said, in my opinion, the work does not constitute a significant, conceptual step forwards for the following reasons:

1. Figures 1 and 2 show that espin and fascin together bundle filaments more extensively than either alone. However, this has been shown previously in earlier publications (PMID: 18579789). Therefore, figures 1 and 2 are technically sound, but the results are confirmatory.

2. Figures 3 and 4 assess bundle stability in the presence of MICAL and cofilin. These experiments are more novel, but the results and conclusions are descriptive. The study would be greatly improved if the authors were to identify underlying mechanisms of bundle disassembly. Does MICAL alter the affinity of espin or fascin for F-actin? How do MICAL, espin, and fascin work alone or together to control cofilin loading on to F-actin? Cofilin severing activity is related to the fractional occupancy on the filament. Do the other 3 factors change the binding rules for cofilin? Does MICAL or cofilin alter the fascin binding/unbinding dynamics seen in simpler systems (PMID: 20551315)? What about for espin? Can a phase diagram be drawn that provides some mechanistic insight into what combinations and concentrations of factors favor bundle formation versus disintegration? These sorts of experiments would help reveal mechanisms that go beyond phenotypic description.

Minor points:

1. The experiments are performed using preformed filaments and then adding bundling proteins. What happens during

copolymerization? Which would better reflect the in vivo situation? Prepolymerize and then bundle, or copolymerize the actin in the presence of the bundling proteins?

2. The in vivo work on bristles was very nice. Could more be done in the discussion to relate the in vitro work to the in vivo phenotypes and current models of bristle-stereocilia formation? Maybe not much more can be said, but I found myself interested to hear more from the authors to relate the current studies back to the historical context of the field. It's just a thought.

In summary then, I think bundle formation and bundle dynamics are important, understudied questions in the actin field. While the current experiments are technically sound, and I agree with the conclusions, the results do not provide mechanistic insight into the observations.

Response to Reviewers

We thank the reviewers for their positive comments on the manuscript. We also appreciate the reviewers for their thoughts, comments, and suggestions for strengthening our manuscript. We believe that we have been able now to address each of their points with new experiments and modifications to the text and figures. In particular, with the new experiments and other additions, we have added extensively to the manuscript, including within the main figures (Figs. 3, 4, 5, 7, and 9) and in the Supplementary Figures (Figs. S1, S2, S4, and S5). We find that the reviewers' experimental suggestions and other helpful comments have added further clarity to our previous results, and have notably strengthened our observations and their significance.

Summary of Revisions:

(1) We have performed the new requested experiments to "*identify underlying mechanisms of bundle disassembly.*" Our results reveal:

[1] Specific underlying **molecular mechanisms** of bundle disassembly: that specific molecules Mical, and Mical in combination with cofilin, robustly disassemble F-actin bundled with different individual bundling proteins. This includes complex F-actin structures that are bundled with two different bundling proteins (espin and fascin) (Figs. 3, 6).

[2] Specific underlying **biochemical mechanisms** of bundle disassembly: [i] Mical requires its NADPH-dependent enzyme activity to disassemble complex bundled filaments (Fig. 3A-C), [ii] espin and espin+fascin-co-bundled filaments directly activate and serve as a substrate of Mical's oxidoreductase enzyme activity (Fig. 7B), and [iii] these Mical-oxidized actin filaments are more poorly bundled by espin, fascin, and espin+fascin compared to unmodified actin filaments (Fig. 7C). These results, which are also suppored by additional requested experiments in Fig. S5 (see (2) below), provide a biochemical understanding of how Mical, and Mical in tandem with cofilin, disassembles complex bundled filaments (summary model in Fig. 7D).

[3] Specific underlying **cellular mechanisms** of bundle disassembly (Fig. 8), including: [i] Mical's disassembly of complex bundled F-actin *in vivo*, [ii] the importance of both espin and fascin for helping to protect F-actin from Mical-mediated disassembly *in vivo*, and [iii] espin + fascin's combined help in protecting F-actin against Mical is critical for regulating the morphology, extension, and remodeling of cells *in vivo*.

Our results therefore identify a first molecular mechanism to disassemble complex bundles composed of two different bundling proteins (espin and fascin), the biochemical mechanism of how these disassembly molecules (Mical and cofilin) are causing this disassembly, and that these molecular and biochemical mechanisms are part of the cellular mechanisms that allow for complex bundled F-actin disassembly *in vivo*. Together, these results are therefore not simply *"descriptive"*, nor simply a *"phenotypic description"*, but *"constitute a significant, conceptual step forward"* into the molecules and mechanisms underlying the disassembly of higher-order bundled F-actin networks.

(2) We have performed the additional new requested experiments that are related to the above request for experiments. Our results reveal:

[1] That without its enzymatic activity, Mical does not alter the affinity of espin or fascin for F-actin (Fig. S5B). So too, our new experiments reveal that the presence of fascin, espin, or both does not alter the binding of Mical to bundled actin (Fig. S5A). However, as mentioned above, once actin filaments are oxidized by Mical, they are more poorly bundled by espin, fascin, and espin+fascin compared to unmodified actin filaments (Fig. 7C).

[2] That at the concentrations used in our assays, neither Mical (without NADPH) nor cofilin alters fascin's or espin's F-actin binding/unbinding dynamics (Figs. S5B, S5D). Yet, as mentioned above, we found that once

Mical oxidizes actin filaments, although the affinity of fascin and espin for Mical-oxidized F-actin is similar to that for unoxidized F-actin (Fig. S5C; compare the Kapp in (1) and (2)), these Mical-oxidized actin filaments are more poorly bundled by espin, fascin, and espin + fascin compared to unmodified actin filaments (Fig. 7C).

[3] That bundling proteins do not assist Mical by increasing cofilin loading onto F-actin, since we find no difference in cofilin's binding to F-actin in the presence of concentrations of espin or fascin used in our TIRFM Mical+cofilin F-actin disassembly assays (Fig. S5E). Instead, these results support previous results that once the Mical enzyme has oxidized F-actin, cofilin binds better to Mical-oxidized F-actin than unoxidized F-actin (Grintsevich et al., *NCB*, 2016; Wioland et al., *EMBO J*, 2021). Thus, as requested by the reviewers, these new observations, together with those new results discussed above and our previous observations, have allowed us to update the model in Fig. 7D: that Mical's oxidation of F-actin alters F-actin's conformation and stability – including weakening espin and fascin's F-actin bundling – and this oxidation of actin by Mical also allows cofilin to bind better and disassemble Mical-oxidized filaments more rapidly than unoxidized filaments.

(3) We have performed new requested experiments that show that espin-bundled filaments resist Mical-mediated disassembly relative to unbundled F-actin (Fig. 4A). These observations, coupled with other related requested experiments (Fig. 7B-C), demonstrate that espin and espin+fascin bundling help protect filaments from Mical-induced disassembly by weakening Mical's activation/oxidation of its F-actin substrate.

(4) As requested, we have performed experiments that show that after disassembly of bundled F-actin by Mical/NADPH, the filaments do not remain as single filaments, but are further converted into G-actin (Fig. 3C).

(5) As requested, we have added a diagram (Fig. 9A) that illustrates what factors favor bundle formation versus disintegration.

(6) We have performed analysis (Fig. S2G) that indicates that fascin and espin's synergistic increase in both bundle length and width are correlated.

(7) We have performed the requested clarification within the text, as well as provided an experiment (Fig. S4B), to better detail the results of some of our light scattering assays. In particular, we discuss how our previous work demonstrated that addition of solution and solution mixing can cause initial marginal decreases in light scattering measurements (e.g., Fig. 1D, Rajan et al., 2023, and we now also show this in Fig. S4B). However, in these situations, this transient reduction in light scattering is reversed with time. In contrast to that, in the presence of Mical/NADPH this disassembly occurs more rapidly and is not reversed with time. These characteristics make light scattering measurements a sensitive and robust means to examine the effects of different proteins (and combinations of them) on bundled F-actin disassembly.

(8) We have performed the requested updates to the text/figures to address the confusion related to the two different ways we presented data related to the fascin:actin ratio. We have now clarified that our data do indeed, as pointed out by Reviewer #1, match previous data from the Alushin lab (Gong et al., *Nat SMB*, 2025) that indicate a saturation of fascin at about ~0.23 ratio to actin.

(9) We have performed the requested updates to the Results and Methods to clarify (and provide in a more centralized location) the species of the proteins we chose to work with. This also includes our rationale for using the splice form of espin we chose to use. We have also updated the Results and Methods to more indepthly discuss how our espin protein purification strategy followed previous published results, and the likely temperature-dependent reasons (performing bundling experiments at 37 °C vs 25 °C) why some differences have been seen in the thickness of espin-bundled F-actin despite the fact that the same espin was used.

(10) We have performed the requested updates to the Methods to clarify several aspects of the experimental approaches, data analysis and quantification – including how bundles were selected for analysis, how regions of interest on bundles were chosen, the details of timing of our TIRFM imaging, the quantification of shortening, thinning, and severing of bundles, and how specific data were presented.

(11) We have updated the text to indicate that since work in our *in vivo* model indicates that filaments are formed and then bundled, we have performed our experiments in a similar way (i.e., pre-polymerization followed by bundling versus co-polymerization with bundling proteins). This type of strategy also allows us to demonstrate that bundled filaments are disassembled versus not being formed.

(12) We have added to the results and discussion to further relate the *in vitro* work to the *in vivo* phenotype and current models of bundle formation in bristle-stereocilia formation.

(13) We have corrected our overlooking of two recent papers on fascin–F-actin's interfacing and inter-filament distance, corrected a noted typo in a figure, and corrected the two stylistic issues noted on the figures. We have also performed the requested unification of our terminology for espin in the manuscript.

Detailed Point-by-Point Response to Each Reviewer:

Referee #1:

We thank Referee #1 for positive comments on the manuscript including: "***This is an interesting and timely question***" and ***"Overall, the work in this manuscript is well done and well presented."*** We also thank the Reviewer for his/her helpful comments on the manuscript including ***"I think the following points need clarification before it can be published."***

Specific Comments:

1a. *"Several aspects of the data analysis and quantification are unclear. For example, is the rate of bundle shortening determined using a population of randomly picked bundles, or only the bundles that were observed to shorten?"*

We thank the Reviewer for these questions and requests for clarifications. These clarifications (and those below (*1b-1h*)) have now been included in the Materials and Methods section under the heading "TIRFM data analysis". Related to this specific question, all analyses were performed by randomly selecting bundles within the field of view. Unbundled actin filaments—identified based on their fluorescence intensity—were excluded from the analysis.

1b. *"How were the regions of interests (for a thinning event, for example) chosen?"*

In the presence of disassembly proteins (Mical/NADPH, cofilin, and Mical/NADPH+cofilin), thinning can occur at any point along the bundle length, either at the ends ("peeling mode" as described previously in Rajan et al., 2023) or between them (i.e., thinning by removal of a small filament segment anywhere in the bundle while the remaining bundle's width stays the same). To avoid bias and to capture both possibilities, we analyzed entire actin bundles, rather than focusing on specific sites.

1c. *"For severing events, are all the bundles candidate sites, or only above a certain intensity or size?"*

To analyze severing events, time-lapse images were manually examined, frame by frame, to identify and count visible severing occurrences. Bundle lengths were measured at the beginning of data acquisition (the start of video acquisition after the addition of the disassembly proteins) using ImageJ, and the number of severing events was

manually counted for each frame starting from t = 2.5 sec after beginning the imaging (see Comment #1h, below), over the subsequent 60 seconds. Furthermore (and see also question *#1e* below), since shorter fragments (<5 µm) tended to diffuse away upon severing (as noted in our previous publication (Rajan et al., 2023)), only bundles measuring 5 µm or longer were included in the analysis. A severing event was defined as a single filament breaking into two or more distinct fragments.

1d. ***"Does the bundle length and thickness remain constant during the 60 seconds during which severing is monitored?"***

In the absence of disassembly proteins, the answer is yes (i.e., bundle length and thickness remain constant during the 60 seconds in which severing is monitored). In the presence of disassembly proteins, the answer is no: bundle length and thickness do not remain constant during the 60 seconds over which severing is monitored. In particular, in the presence of these disassembly proteins, we found that bundles undergo significant thinning at specific sites along actin bundles. Thus, bundles thickness does not remain constant in the presence of these disassembly proteins. Further, as these disassembly proteins induce bundles to become progressively thinner over time, they become more prone to severing by them. Thus, bundles length also does not remain constant in the presence of these disassembly proteins. We also observe that in the presence of these disassembly proteins, bundles length decreases not only due to thinning/severing, but also through depolymerization at the bundles ends. This bundles depolymerization process becomes more pronounced over time (as the bundles are thinned/peeled at their ends). In the case of Mical/NADPH + cofilin, this change in bundles length and thickness by thinning, severing, and depolymerization occurs rapidly and simultaneously. Therefore, to quantify these changes and try to differentiate them between the additions of the different disassembly proteins (Mical/NADPH only, cofilin only, and Mical/NADPH + cofilin), we monitored these events during the initial 60 seconds (after beginning the imaging (see Comment #1h, below)) after the addition of low amounts of these disassembly proteins. Using this time scale and low amounts of disassembly proteins, we found that notable bundles shortening and thinning events did occur with Mical/NADPH only and cofilin only in the presence of all three bundles (Figs. 4C (2-3), 5B (2-3)), but severing was minimal (consequently, the standard error of the mean (s.e.m.) values closely matched the mean values) (Figs. 4C(4), 5B (4)). In the presence of Mical/NADPH + cofilin, substantially more shortening, thinning, and severing of bundles occurred (Fig. 6B (3)).

1e. ***"When a severing event occurs, can the fragments continue to be monitored or do they diffuse away?"***

The fate of fragments generated by severing typically depended on their length. Longer fragments (≥5 µm) generally remained visible and were sometimes tracked further if they stayed within the field of view. Shorter fragments (<5 µm) tended to diffuse away, and as in our previous publication (Rajan et al., 2023), were excluded from the analysis.

1f. *"On page 25, the explanations for the way the percentage of remaining bundles was computed is unclear. Were fragments counted as gone when they became shorter than 7 µm?"*

Yes, this comment (previous page 25) relates to Fig. 6B (2): the effects of Mical/NADPH+cofilin on the three different types of bundles (fascin alone, espin alone, or fascin+espin) and is now better detailed in a "TIRFM Data Analysis" section of the Materials and Methods. In particular, based on our observations, we sought to find a quantitative measure that best highlighted the differences we were observing that Mical/NADPH+cofilin was having on the bundle types. For our calculations, we randomly selected 10-15 bundles per bundle type, and monitored their length over time. The bundles with decreasing length (over time), in the presence of disassembly proteins (due to end shortening or severing), that reached <7 µm were omitted from the final bundles present in the field of view. This was done because severing leads to an increased number of bundles fragments, the majority of which are relatively small – and thus could either randomly decrease (via diffusing away) or increase (via making one bundle into many) the number of bundles. Thus, in order to simplify the quantification of the effects

of Mical/NADPH+cofilin on the three different types of bundles, we focused only on longer fragments of bundles (≥7 μm).

<u>1g.</u> *"In the legend of Figure 3, the authors explain that bundle thinning rates are determined by an exponential fit as shown in Fig S4D, but in Fig S4D the fits are not shown and the data do not look like exponentials. Looking in the Methods, things get more confusing because the data is said to be "fitted by a linear regression" (page 24). This should be clarified. What would make more sense, a linear decrease or an exponential decrease?"*

Our apologies for the confusion regarding the determination of the thinning rates. We calculated the thinning rates of bundles at two different times: the initial thinning rate (over the first 120 seconds), and the overall thinning rate (across the entire duration of data acquisition). To improve clarity, we have now reorganized the relevant section and included this in the TIRFM Data Analysis section of the Materials and Methods.

Each time frame offers distinct insights. The initial rate reflects how rapidly bundles disassembly begins following the addition of disassembly proteins. It is not affected by the potential re-association of severed fragments later in the reaction. In contrast to that, the overall average rate captures the progression of the entire disassembly process, from start to finish.

We included both sets of data in the manuscript because they highlight different aspects of the reaction dynamics. However, based on our understanding, the initial thinning rate is more informative and has therefore been presented in the main figure (Figs. 4B (3), 5B (3), 6B(3)), while the overall thinning rate is included as supporting information. The exponential fits lines are now added in Figs. S3B and S4D (previously *Figs. S4D* and *S7B*).

<u>1h.</u> "*Also confusing, though it is more of a detail, the arrowheads in Fig 3F (and Fig 4B) suggest that thinning and shortening (and severing, in 4B) are already visible at t=0. I assume this is not the case.*"

We apologize for the confusion on this issue. There was a necessary delay of approximately 1 minute between the addition of the disassembly proteins and the start of video acquisition (the time it took to add the proteins, mix the samples, and then focus and begin the imaging). This has now been made clearer in the Materials and Methods, the Results, and in the Figures/Figure legends. In particular, to prevent confusion, we have now updated the time in the images accordingly to reflect this delay. Thus, since ~60 secs have passed before data acquisition, the thinning and shortening of bundles as indicated by arrowheads in Figs. 3F and 4B (now Figs. 4C and 6B), as well as severing events (now Fig. 6B), are in fact observable at t=0 sec (now listed as t=60 sec). This is particularly evident in samples in which both Mical/NADPH and cofilin were added, as their combined effect significantly accelerates bundles disassembly (now Fig. 6B).

<u>2.</u> "*I find the measured ~0.6 fascin/actin (Fig S2C) hard to understand. Recent structural data from the Alushin lab (Gong et al., Nat SMB 2025, not cited) reports a spacing of 13 actin subunits between fascins that bridge two neighboring filaments. With 6 neighbor filaments (hexagonal lattice) that would mean a maximum fascin:actin ratio of 1/13*6/2=0.23 (the last division by 2 is because each fascin is shared by 2 filaments). This is an overestimate because it neglects the fact that filaments at the bundle periphery will not have as many fascins. Regardless of this last point, the fascin:actin ratio reported here is far above the expected maximum. Note that, in contrast with Fig S2C, Fig 1C seems to indicate a saturation of fascin at a ratio below 0.25 ("bound fascin/actin"). All this should be explained, or at least discussed.*"

We thank the Reviewer for pointing out this confusing issue, and we have now modified the manuscript with this comment in mind. As you mention, our own data (in Fig 1C) and the Alushin lab results (Gong et al., *Nat SMB*, 2025, now cited), indicate a saturation of fascin at a ratio to actin of about ~0.23. Thus, our results using Drosophila fascin are in agreement with this study using human fascin-1, and we have now included this information in the Results section. In contrast, the measured/listed ~0.6 fascin/actin in the previous Fig S2C

denotes the fraction of fascin bound to pelleted F-actin out of the total protein added (2μM). For these competitive binding assays between the two proteins (fascin and espin) we used their high concentrations. Thus, to try to rectify this confusion, we have now replotted the graph for Fig S2C (now Fig S1F), with calculations including the final concentration of each protein used during the assays, i.e. fascin=2μM, and F-actin=5μM. This shows that the ratio of fascin to actin is ~0.23, and is not changed in the presence of the other bundling protein (espin).

3a. *"Information on the proteins is missing. The methods indicate (page 20) that fascin and Mical have been purified "as previousy described", but it would be nice to know at least what orthologs were used. Cofilin is said to be human, but which isoform is it? I assume it is cofilin-1, but it would be worth saying so explicitly.*

We thank the Reviewer for bringing up these points. We have now modified the Materials and Methods section, mentioning the details of the proteins used in our study (including that, as this reviewer mentions, we do use human cofilin-1). In particular, we used human cofilin-1 to compare these present results with our previous results (Grintsevich et al., 2016; Rajan et al., 2023) and those present in the literature. We have also mentioned our reasoning for using Drosophila Mical and Drosophila fascin: we used these forms of the proteins (as we did in our previous study; Rajan et al., 2023) to allow us to more closely match our *in vitro* observations with our *in vivo* observations. We also mention our reasoning for using a splice form of mammalian espins that is known to be able to rescue the *espin (forked)* mutant defects in Drosophila (Grieshaber et al., 2001; and see our response to Comment #3b below).

3b. "*On page 4: "Espins comprise four isoforms" in which species? The in vitro work here is done with espin-4 which is a somewhat peculiar (short) version of espin. This specificity is worth mentioning, and it should be discussed when comparing with published observations on other espins (in the discussion section).".*

We thank the Reviewer for this question and the need for clarification on this issue. We previously had this information within different areas of the manuscript, but have now added this information to the text at the beginning of the Results section. In particular, the single *espin* gene in mammals has been described by the discoverer of the espins (James Bartles) as having four major splice variants/isoforms (Espin-1, Espin-2 (A and B), Espin-3 (A and B), and Espin-4; e.g., reviewed in Sekerková et al. (Bartles), 2006). It is important to note that each of the espin splice forms (including espin-4 (also called small espin)) share a common 14 kDa C-terminal actin binding domain, which is necessary and sufficient for F-actin bundling activity (Bartles et al., 1998; Bartles, 2000). It is also important to note that this espin-4 splice form has been used by different labs to study the action of espins (e.g., Bartles et al., 1998; Claessens et al., 2008). Furthermore, some of the longer espin splice forms and their use *in vitro* to bundle F-actin are complicated by having domains that can act in an autoinhibitory fashion (e.g., Zheng et al., 2014; Liu et al., 2016). Thus, the major reasons that we used this splice variant for our *in vitro* work is because this small espin splice variant (the espin-4 isoform): [1] contains the actin bundling regions of espin that are necessary and sufficient for actin bundling (Bartles et al., 1998; Bartles, 2000), [2] it has been used in other prominent foundational studies (e.g., Bartles et al., 1998; Claessens et al., 2008), [3] it most closely matches Drosophila espin (forked), and [4] this mammalian espin-4 splice form is sufficient to rescue the defects that occur *in vivo* when *Drosophila forked (espin)* is removed (i.e., the bundling defects present in *Drosophila forked (espin*) mutants can be rescued by expressing this mammalian espin-4 splice form (Grieshaber et al., 2001)). Thus, this form of espin allowed us to robustly bundle F-actin *in vitro* and match our *in vitro* work with our *in vivo* work.

Minor:
1. **"***Page 21. It is unclear to me why polymerization is done in 10x buffer instead of 1x buffer."*

We thank the Reviewer for this question and we have now corrected the confusion on this issue by revising a sentence in the Methods. In particular, we used a 10× polymerization buffer stock, but with a final concentration of 1× in the polymerization reaction.

2. *"Statistics for EM (Fig 1F). The table indicates that "5 or more bundles" were analyzed. This is a rather low number. How were these bundles chosen? Randomly?"*

We thank the reviewer for this question and the request for clarification, which we have now included in the Materials and Methods. Yes, actin bundles were imaged in a randomized manner, across multiple grids squares that were identically fixed and stained, with no bias toward a specific morphology or size. Though we observed more than 5 bundles per conditions, not all bundles were suitable for analysis (because of aggregation and overlap). Thus, using these criteria, only regions in the bundles where individual filaments could be clearly observed to make reliable measurements were considered. As detailed in the Results section, our observations using this approach reveal that the general trend indicated by our data is consistent with observations using other approaches (e.g., a higher ratio of espin to F-actin results in larger bundles, and because of the size of the bundler, the interfilament distance is smaller in espin-bundled vs fascin-bundled filaments). Our results using this approach are also similar to that previously reported (e.g., the parameters we measured with fascin (1:1 actin) are similar to those previously reported (e.g., Ishikawa et al., 2003; Jansen et al., 2011), and fascin + espin results in larger bundles than those with either fascin or espin on their own (e.g., Claessens et al., 2008)).

3. *"In Fig 3A, the red circle could be misunderstood as representing MICAL (in spite of the legend, which is a bit ambiguous)."*

We thank the Reviewer for this comment and we have now modified both the figure and the legend for Fig. 3A (now Fig. 7A).

Referee #2:

We thank Referee #2 for positive comments on the manuscript including: ***"This study provide important insights into the dynamic assembly and disassembly process of higher-order actin structures crosslinked by fascin and espin.", "Overall, the experiments are well-designed and the results are clearly presented.",*** and ***"This study makes an important contribution to our understanding of the remodeling and plasticity of filament bundles crosslinked by multiple crosslinkers."***

We also thank the Reviewer for his/her helpful comments on the manuscript including ***"Below are a few specific comments and minor points which I believe should be addressed prior to acceptance for publication":***

Specific Comments:

1. *"The fascin-F-actin interface and the inter-filament distance have been precisely defined by recent studies (PMID:39833469, 40403900). Thus, the statement on page 5 citing "three putative actin-binding sites" from earlier studies is inaccurate and should be updated."*

Thank you for bringing this to our attention. We have made the correction and added the references mentioned.

2a. *"Espin in this study was purified by affinity chromatography without further polishing steps such as ion exchange or size exclusion. As shown in Supplementary Figure 1A-2, the flowthrough after tag removal contains substantial amounts of contaminating proteins. In S1A-3, the contaminating bands below purified espin are not shown."*

We thank the Reviewer for this comment and we have now further detailed/documented our purification strategy and have mentioned these issues in the legend of Fig S1A and in the Materials and Methods. In particular, we followed the purification instructions developed by James Bartles (who originally identified espins)

– and sent it to us when he sent us the DNA construct. This strategy has also been used by others who have prominently worked with it (e.g., Purdy et al., *Physical Review Letters*, 2007; Claessens et al., *PNAS*, 2008; Kitajiri et al., *Cell*, 2010). Furthermore, as brought-up by this reviewer, many of the recombinant proteins purified (including in our two labs (Terman and Reisler labs)) do indeed use further polishing steps such as ion exchange and size exclusion. However, when we attempted to use these polishing steps on espin, it removed much of the espin protein. Therefore, we followed the approaches mentioned above that were used in previously published work to purify espin. Furthermore, since we have used the same construct and purification strategy as developed by James Bartles, and used in a number of espin papers, we believe this issue is not unique to us. Indeed, both our labs (Terman and Reisler) used this approach and we see similar results. Moreover, none of the previous papers (e.g., Bartles et al., *Journal of Cell Biology*, 1998; Purdy et al., *Physical Review Letters*, 2007; Claessens et al., *PNAS*, 2008; Kitajiri et al., *Cell*, 2010) show, as we do here for transparency, the steps and quality of the purified protein. In addition, our antibody results reveal that these are not contaminating proteins but degraded forms of espin. In light of this susceptibility of espin to degradation, we used fresh batches of espin in our experiments. Lastly, as we show in multiple figures and with multiple approaches (sedimentation, light scattering, TIRFM, and EM), our purified espin robustly bundles F-actin.

2b. *"Consequently, the effective concentration of espin used in this study may be considerably lower than measured, which potentially affect all concentration-dependent results. For instance, espin crosslinks F-actin into hexagonal bundles with a saturating stoichiometry of approximately 1:4.33, yet the bundles in Figures 1E and S3F appear extremely thin, even at saturating or near-saturating espin. In contrast, much thicker bundles were observed under similar conditions at lower espin ratio (1 espin per 7 actin subunits, PMID: 9763424). Please clarify the possible reasons for this discrepancy."*

We thank the Reviewer for this comment, which we have now discussed in the legend of Fig S1A and in the Materials and Methods section. In particular, the other bands that can be seen using our purification strategy (which are espin degradation products, see above), are minimal compared to the abundant espin band in Fig S1A. Thus, we respectfully believe that the effective concentration of espin used in this study would not be considerably lower than measured. Furthermore, the author of PMID: 9763424 (Bartles et al*., JCB*, 1998) sent us this espin construct: he used it in PMID: 9763424 and used the same purification strategy as we do. Therefore, it is likely that if the effective concentration of espin is slightly off in our study, it would be slightly off in that study too. Morever, the much thicker bundles observed in PMID: 9763424 may be due to the difference in the temperature at which actin filaments were incubated with espin in that study versus ours. In PMID: 9763424, the experiments were performed at 37°C, while our assays (for negative-stain EM preparations) were carried out at room temperature. The higher temperature conditions would likely enhance actin binding kinetics to promote more extensive crosslinking between espin–actin filaments, resulting in thicker bundles. Lastly, even if the effective concentration used in our study was off, it would not affect our main results or conclusions such as: [1] we used multiple different approaches to show this espin is bundling F-actin, [2] we used different approaches to show Mical disassembles this espin-bundled F-actin, [3] we find that there is a synergy between fascin and this espin in bundles assembly, and [4] we find that fascin and this espin together protect F-actin from Mical and Mical + cofilin to a greater extent than equimolar amounts of fascin or espin on their own.

3. *"Since fascin and espin synergistically increase both bundle length and width, please provide data or analysis demonstrating whether these two parameters are correlated."*

We thank the Reviewer for this question. Our analysis points to the idea that these two parameters are correlated. In particular, for exact correlation determination, the length and width of the same actin bundles is required. As mentioned in the manuscript, we found that due to fluorescence intensity saturation (after 5 actin filaments; Fig S2E), the thickness of bundles could not be measured using TIRFM assays (which were used to determine the length of the bundles). Furthermore, EM analysis does not allow us to measure over time the length of the filaments/bundles. However, to examine the Reviewer's question and see if our results at least point to the

idea that these two parameters are correlated, we plotted our results using TIRFM assays to determine the length of the bundles and EM assays to determine the width of the bundles. Our results (presented in Fig S2G) point to the idea that these parameters are correlated.

4a. *"Figure 3 focuses on Mical mediated bundle disassembly by tracking changes in bundle integrity, but it does not reveal the nature of the disassembly products. After disassembly, do the filaments remain as single filament, or are they further converted into G-actin? Such information would provide important insight into the dynamics of bundle disassembly by mical. At minimum, include high-speed co-sedimentation assay to track whether the disassembled bundles are all converted into G-actin."*

We thank the Reviewer for this question and we have now conducted the new experiments the reviewer suggested to address it. These new experiments reveal that after disassembly of bundled F-actin by Mical/NADPH, the filaments do not remain as single filaments, but are further converted into G-actin (Fig 3C).

4b. *"Related to this, the model in figure 3A shows mical disassembles the filament into G-actin, whereas the model in supplementary 6A suggests that mical oxidized filaments remain intact, which is further disassembled into G-actin by cofilin. Tracking the disassembly products would enable a more detailed and consistent mechanistic model."*

We thank the Reviewer for this question and our results (including new experiments) using high-speed sedimentation assays, low-speed sedimentation assays, pyrene actin assays, light scattering assays, and TIRFM assays, allow us to track the disassembly products and indicate a more detailed and consistent mechanistic model. In particular, our results of all these assays indicate that the extent of the bundled F-actin disassembly (from unmodified bundled F-actin to Mical oxidized filaments to G-actin) depends on: [1] the amount of Mical/NADPH present, [2] the time of the incubation with Mical/NADPH, [3] the amount of bundling proteins present, [4] the type of bundling proteins (fascin alone, espin alone, or fascin + espin), and [5] whether cofilin is also present with Mical/NADPH. For example, in the presence of Mical, filaments do not remain as single filaments but are further converted to G-actin (Fig. 3C). However, if these experiments are conducted in the presence of actin bundling proteins/bundled F-actin, filaments have more protection from similar amounts of Mical's action (e.g., Fig. 4A). So too, the rate and extent of Mical's disassembly action on these bundled filaments, including the length of time filaments persisted, is also dependent on both the amount and type of bundling proteins present (e.g., Figs. 4B-C, S3). Furthermore, the rate and extent of Mical's disassembly action on these bundled filaments, including the length of time filaments persisted, is also dependent on whether cofilin is present with Mical (e.g., Figs. 6, S4A, S4C-E). We have now better detailed these observations in the model in Supplementary Fig. 6A (now Fig. 7D). Also, based on a related question by Reviewer #3, Question #2f, we have added another diagram that details these observations (Fig. 9A).

5. *"Do espin-bundled filaments resist Mical-mediated disassembly relative to unbundled F-actin? If so, how do they stimulate Mical activity at a similar rate (Fig. 2B)? Please reconcile these observations."*

We thank the Reviewer for these questions and we have now conducted experiments to address them. First, we now show results that point to espin-bundled filaments resisting Mical-mediated disassembly relative to unbundled F-actin (Fig. 4A). Further, our previous results pointed to the idea that there might be somewhat less ability of espin-bundled F-actin to stimulate Mical's activity. However, we did not conduct those experiments to test for that possibility. We simply conducted those experiments to see if espin-bundled F-actin was able to stimulate Mical's activity (versus the espin crosslinks preventing Mical's activity from being stimulated by F-actin). In light of this reviewer's question, we have now conducted these enzymatic assay experiments to address both issues. In particular, we used less Mical (i.e., similar to what we used for the experiments in Fig. 4A, showing that espin-bundled filaments resist Mical-mediated disassembly relative to unbundled F-actin) so that we could both: [1] see if espin-bundled F-actin was able to stimulate Mical's activity and [2] better quantify any differences

between espin-bundled actin's ability to stimulate Mical's activity compared to unbundled F-actin. These new results (now in Fig. 7B) show that espin-bundled F-actin stimulates Mical's activity, but at a lower rate than unbundled F-actin.

__6a.__ "*In figure 3E, the green curve declines gradually, consistent with Mical-driven disassembly of fascin-espin-4 bundle. However, in the similar experiment shown in figure 4A-2, the green curve first decreases but then rises sharply to a level comparable to the starting point. This pattern is inconsistent and difficult to interpret, and the blue curve in this panel shows a similar trend."*

We thank the Reviewer for the question and we have now better clarified this in the mansuscript. In particular, please note that the difference between the green curve in Figure 3E (now Fig. 4B (1)) versus Figure 4A-2 (now Fig. 6A (1), 2nd panel from left), is that there is twice as much Mical added in the experiments in Fig. 4B (1) than in Fig. 6A (1) (50nM vs 25nM). The same is also true for the blue curve (i.e., twice as much Mical added in the experiments in Fig. 4B (1) than in Fig. 6A (1) (50nM vs 25nM)).

__6b.__ *"Moreover, in the first panel the green endpoint exceeds the starting value, suggesting increased bundling in the presence of cofilin. Please clarify these discrepancies or optimize the experiments to obtain more consistent results."*

We thank the Reviewer for this question related to the green curve in the first panel of Fig. 4A (now Fig. 6A (1)). We have now described in the Materials and Methods section and provided an additional experiment (Fig. S4B) for why we see these types of effects/curves (and this response also applies to Comment #6a, above). In particular, in these light scattering measurements, the addition of solution and its mixing can cause initial effects (since these measurements are particle size sensitive real-time measurements). We have previously noted that this leads to marginal decreases in the light scattering intensity (e.g., see our previous paper (such as Fig. 1D, Rajan et al., 2023) in which we first optimized these light scattering assays). However, this type of transient reduction in light scattering is reversed with time (e.g., Fig. 1D, Rajan et al., 2023; and see new Fig. S4B). However, in the presence of Mical/NADPH, this disassembly occurs more rapidly and is not reversed in time (e.g., see Fig. 1D of Rajan et al., 2023, and Figs. 4B and 6A of this paper). These characteristics make light scattering measurements a sensitive and robust means to examine the effects of different proteins (and combinations of them) on bundled F-actin disassembly.

We therefore took advantage of these previous observations for the present paper. In particular, we used low amounts of cofilin alone or Mical/NADPH alone – so that on their own in the presence of fascin or fascin + espin they had very little to modest effects on the decrease in light scattering. For example, the effects we see with low levels of cofilin are not that much different (Fig. 6A (1) in cofilin only graph in this paper, green and blue lines in cofilin only) than with the addition of buffer only (see Fig. 1D, Rajan et al., 2023, and Fig. S4B of this paper). These results indicate that this amount of cofilin has very little to no effect on fascin and fascin+espin bundles. So too, this low amount of Mical (Fig. 6A (1) in Mical only graph) has relatively modest effects on the light scattering of fascin and fascin+espin bundles. Thus, employing this experimental strategy and comparing the panels in Fig. 6A (1), leads us to the robust observation of the strong loss of light scattering in the presence of Mical/NADPH + cofilin compared to Mical/NADPH or cofilin on their own. It should also be noted that we have complemented our light scattering assays with pyrene actin and TIRF assays (e.g., Figs. 6B, S4A, S4C-E), and they support the results of these light scattering assays.

__Minor points__:
__1.__ *"Espin and espin-4 are interchangeably used throughout the figures and captions, the terminology should be unified for consistency."*

We thank the Reviewer for this comment and we have now unified our terminology for espin. In particular,

we have described the espin-4 splice variant at the beginning of the Results section and then used espin throughout the manuscript. See also our response to Reviewer #1, Question 3b in which we describe our rationale for using the espin-4 splice variant (including that it has the espin actin bundling region that is necessary and sufficient to bundle F-actin and that it can rescue the defects found in *forked/espin* mutants *in vivo* in *Drosophila*).

2. *"In figure 3B-1, I assume the Mical only and Mical + Espin-4 curves are overlapping. However, the Mical only curve is not discernable. Please adjust styling to ensure both curves are clearly distinguishable."*

We thank the Reviewer for this question and we have now made the change. In particular, yes, these curves Figure 3B-1 (now Figure 7B (1)) were overlapping. We have now adjusted the styling so both curves are clearly distinguishable.

3. *"In figure 4B-2, the espin concentration is labeled as 0.2mM but should be 0.2 uM, please correct this."*

We thank the Reviewer for bringing this to our attention. We have now corrected it in what is now Fig. 6B (2).

Referee #3:
We thank Referee #3 for positive comments on our manuscript including: ***"Assembly, maintenance, and turnover of filament bundles are important question in the field, and in vitro reconstitution with confirmation of principle in vivo is an excellent approach."***, ***"The in vivo work on bristles was very nice"***, ***"In summary then, I think bundle formation and bundle dynamics are important, understudied questions in the actin field"*** and ***"…the current experiments are technically sound, and I agree with the conclusions".***

We also thank Referee #3 for her/his helpful comments/recommendations on the manuscript:

General Comment:
1. *"This manuscript is divided into two sections. The first half focuses on bundle formation to show that espin plus fascin are more effective at bundling than either alone. The second half of the manuscript focuses on bundle stability to show that bundles built through a combination of espin and fascin are more resistant to MICAL and cofilin disassembly than bundles built from either factor alone….However, that said, in my opinion, the work does not constitute a significant, conceptual step forwards"*

We thank the Reviewer for this comment. Yet, it is important to respectfully point out that this overview does not fully represent the findings of our study and its significant, conceptual step forward. In particular, as we have outlined in the introduction of our manuscript, actin bundles have to undergo tightly regulated disassembly and turnover to allow cells to respond to environmental signals and undergo functional remodeling and have plasticity (Blanchoin et al., 2014; Lappalainen et al., 2022; Valencia et al., 2021). However, the molecules and mechanisms underlying this regulated disassembly of bundled F-actin are still poorly understood (e.g., Breitsprecher et al., 2011; Chikireddy et al., 2024; Elkhatib et al., 2014; Schmoller et al., 2011). Indeed, a recent study in the *Journal of Cell Biology* (Chikireddy, J., L. Lengagne, R. Le Borgne, C. Durieu, H. Wioland, G. Romet-Lemonne, and A. Jegou. 2024. *Fascin-induced bundling protects actin filaments from disassembly by cofilin*. *J Cell Biol*. 223: e202312106) highlights this issue – of [1] trying to understand how the complex higher-order F-actin structures are disassembled (since cofilin is not a robust disassembler of them), and [2] the high-level interest in this area for the cell biology community. Furthermore, to the best of our knowledge, the ability of disassemblers to destabilize bundles composed of multiple different bundling proteins has not been examined previously. Thus, we have no understanding of/answers to this problem. Yet, this is of critical interest, since F-actin is often simultaneously and redundantly bundled by several different proteins.

Therefore, we have begun to investigate these important questions. Our initial examination of them (Rajan et al., *PNAS*, 2023), has shed light on some of these questions: that Mical, and also Mical combined with cofilin, are robust disassemblers of fascin-bundled F-actin. In the present study, we addressed other important unanswered questions such as: [1] does Mical affect F-actin crosslinked with other critical bundling proteins (versus the possibility that Mical's effects on bundled F-actin only applies to fascin crosslinked F-actin bundles), and [2] how is F-actin bundled with multiple different bundling proteins disassembled. Furthermore, in the process of our investigations into the above questions, our experiments provided new conceptual advances into the mechanisms of bundled filaments assembly. **Thus, we now find that our extensive qualitative and quantitative analysis – using a wide range of *in vitro* and *in vivo* biochemical, genetic, structural, and cellular approaches (including updating this revised version of the manuscript in response to each of the Reviewers' requests for new experiments and clarifications) – has led us to 12 main findings. It is also important to note that all of these 12 main findings are novel, have not been previously published, and provide definitive new insights into the molecular and biochemical mechanisms by which bundled F-actin is robustly assembled and disassembled. Furthermore, since prior to this study none of these 12 main findings/effects/mechanisms were known, we believe that our study does *"constitute a significant, conceptual step forwards"* into the molecules and mechanisms underlying the assembly and disassembly of higher-order bundled F-actin networks. Our 12 novel main findings in this paper are:**

[1] Using purified fascin and espin, our results extend previous work on fascin and espin being robust bundling proteins. Our results support the point that although fascin and espin are known to induce comparable structural changes in F-actin, they do not interfere with each other's interactions with F-actin, and that fascin is a more potent actin bundling protein than espin (Figs. 1, S1; Table 1).

[2] Using sedimentation, light scattering, TIRF, and electron microscopy assays, our results indicate that when purified fascin and espin are added together, they have a synergistic effect on bundling F-actin – i.e., they are more effective together at bundling F-actin than the equimolar amounts of espin or fascin on their own (Figs. 2A-B, S2A-C). Our results also reveal that this synergy between fascin and espin results in an increase in the length and width of actin bundles (compared to equal molar concentrations of either bundler alone) (Figs. 2C-F, S2D-G). Together, these results show that two different actin-bundling proteins can work synergistically to promote bundles formation – even at concentrations that on their own they would be unable to readily do so.

[3] Our results find that purified Mical robustly disassembles espin-bundled F-actin (Fig. 3A).

[4] Our results find that purified Mical robustly disassembles complex F-actin bundles: i.e., where F-actin is simultaneously bundled together with two bundling proteins – espin and fascin (Fig. 3B).

[5] Our results find that purified cofilin disassembles espin–bundled F-actin and espin–fascin co-bundled F-actin (Fig. 5).

[6] Our results find that Mical synergizes with cofilin to robustly increase the rate and extent of the disassembly of espin-bundled and espin–fascin co-bundled F-actin compared to either Mical or cofilin alone – as observed by light scattering, pyrene-actin, and TIRF microscopy assays (Figs. 6, S4).

[7] Our results find that Mical and cofilin directly induce the disassembly of espin-bundled and espin–fascin co-bundled F-actin via depolymerization/shortening, thinning, and severing of bundles (Figs. 6B, S4C-E).

[8] Our results find that compared to unbundled F-actin – or even F-actin bundled with espin or fascin alone – espin–fascin co-bundled F-actin helps protect it from disassembly by Mical, cofilin, and Mical + cofilin (Figs. 4, 5, 6, S3, S4A, S4C-E). Thus, these complex bundles – composed of different bundling proteins – are significantly more stable and resistant to F-actin disassembly factors than bundles composed of equal amounts of either bundler alone. This synergistic protective effect against F-actin disassembly factors provides a new, previously unknown purpose for why F-actin is bundled with multiple bundling proteins.

[9] In addition to these new molecular mechanisms that we have identified to disassemble complex bundled filaments ([3]-[7] above], we find that: [i] Mical requires its NADPH-dependent enzyme activity to disassemble complex bundled filaments (Fig. 3), [ii] espin and espin+fascin-bundled F-actin directly activate this enzyme activity of Mical (Fig. 7B), and [iii] Mical-oxidized actin filaments are more poorly bundled by espin and espin+fascin compared to unmodified actin filaments (Fig. 7C). These results, which are also suppored by

additional requested experiments in Fig. S5, provide a biochemical understanding of how Mical, and Mical in tandem with cofilin, disassembles complex bundled filaments (summary model in Fig. 7D).
[10] Our results also provide a biochemical understanding of how these complex bundles help protect F-actin from Mical. Namely, these complex bundles decrease Mical's enzymatic actin oxidation activity (Fig. 7B), which is necessary for Mical to disassemble F-actin/bundled F-actin.
[11] Our results support the point that this partial protective ability of espin, fascin, and espin + fascin against Mical-mediated disassembly of F-actin also occurs *in vivo* (Fig. 8D-I).
[12] Our results find that espin + fascin's combined partial protection of F-actin against Mical is critical for regulating the morphology, extension, and remodeling of cells *in vivo* (Fig. 8).

Specific Comments:
1. *"Figures 1 and 2 show that espin and fascin together bundle filaments more extensively than either alone. However, this has been shown previously in earlier publications (PMID: 18579789). Therefore, figures 1 and 2 are technically sound, but the results are confirmatory."*

We thank the Reviewer for this comment and that s/he finds that our results are technically sound. However, it is important to respectfully mention that the results in Figure 2 – that espin and fascin synergize in bundles assembly – have not been previously shown. In particular, as mentioned in novel main findings **[2]** above, we find using sedimentation and light scattering assays that purified fascin and espin added together are more effective in bundling F-actin than the sum of their individual effects – i.e., they show a synergistic effect in the bundling of F-actin (Figs. 2A-B, S2A-C). In other words, our results don't just show that *"espin and fascin together bundle filaments more extensively than either one alone"* but that when present together, fascin and espin can bundle F-actin at concentrations that on their own they would be unable to readily do so. Furthermore, we used TIRF and electron microscopy assays to further investigate this synergism. Our results using these approaches confirm this synergism and reveal that it results in an increase in the length and width of actin bundles (compared to equal molar concentrations of either bundler alone) (Figs. 2C-F, S2D-G). Together, these results show that two different actin-bundling proteins can work synergistically to promote bundles formation – even at concentrations that each one alone would be unable to readily do so. Thus, these are important observations related to the mechanisms of the assembly of actin bundles and the need/role of different bundling proteins in this.

It should also be noted that we heavily cite the groundbreaking work of PMID: 18579789 (Claessens et al., *PNAS*, 2008), which shows that "*espin and fascin together bundle filaments more extensively than either alone"*, as well as the work of others looking at espin, fascin, and espin and fascin *in vitro* and *in vivo*. Indeed, this is presented as one of the foundations of our study, as we began to assemble bundles composed of both bundling proteins. Yet, as described above, we also identified important new mechanisms in F-actin bundles assembly: the synergism between espin and fascin. This was not investigated by PMID: 18579789 or – to the best of our knowledge – in any previous study. Thus, we respectively point out that the results in Figure 2 are novel (and Figure 1 provides the foundation for those studies and additional novelty as described above in novel main findings **[1]**), and are not simply confirmatory of previous work.

2a. *"Figures 3 and 4 assess bundle stability in the presence of MICAL and cofilin. These experiments are more novel, but the results and conclusions are descriptive."*

We thank the Reviewer for this comment and we have now performed the experiments s/he suggests below in 2b-g. Therefore (and aided by these additional experiments this reviewer suggests), as we detail in response to general comment #1 above (**novel main findings [3-10]**), we respectfully submit that: [1] these results are not *"more novel"* – they are novel, and [2] that the results and conclusions are not simply *"descriptive"*, but provide an understanding of how complex bundles – including super bundles formed in the presence of multiple different bundling proteins – are robustly disassembled. Thus, our results provide a first **molecular and biochemical and cellular (*in vivo*)** mechanistic understanding of how this occurs. Furthermore, given the interest in this in the cell

biology community (e.g., see Chikireddy, J., L. Lengagne, R. Le Borgne, C. Durieu, H. Wioland, G. Romet-Lemonne, and A. Jegou. 2024. *Fascin-induced bundling protects actin filaments from disassembly by cofilin. J Cell Biol*. 223: e202312106), our results provide now an important conceptual understanding of how complex bundles can be robustly disassembled. The disassembly steps include: [1] Mical binds to bundled F-actin, [2] after bundled F-actin binding, Mical becomes activated to consume NADPH (its co-enzyme), [3] Mical oxidizes F-actin within bundles, [4] cofilin is then drawn to preferentially associate with the Mical-oxidized actin in the filaments, [5] cofilin then helps Mical to disassemble these actin bundles by their shortening/depolymerization, thinning, and severing, [6] Mical-oxidized actin does not repolymerize well, and [7] espin and fascin do not readily bundle Mical-oxidized actin filaments. See also the model based on our results in Fig. 7D.

2b. "*The study would be greatly improved if the authors were to identify underlying mechanisms of bundle disassembly.*"

We thank the Reviewer for this comment and we have now conducted new experiments (Figs. 7B-C, S5) to address it. Also, we hope that together with the points raised above in General Comment 1 (novel main findings [3-12]), Specific Comment 2a, and below in Specific Comments 2c-g, it is now clear that **we have identified underlying mechanisms (molecular, biochemical, and cellular mechanisms) of bundle disassembly**, including the importance of both espin and fascin for helping to protect F-actin from Mical-mediated disassembly *in vivo*. Our results therefore identify the first reported molecular means to disassemble complex bundles composed of two different bundling proteins and the biochemical means of how Mical and cofilin are causing this disassembly.

2c. *"Does MICAL alter the affinity of espin or fascin for F-actin?"*

We have now conducted experiments to address these questions. In particular, we find that Mical (with or without its actin oxidation activity) does not alter the affinity of espin or fascin for F-actin (Figs. S5B-C). So too, our new experiments reveal that the presence of fascin, espin, or both does not alter the binding of Mical to bundled actin (Fig. S5A). However, we found that in the presence of Mical's enzymatic actin oxidation activity, these Mical-oxidized actin filaments are more poorly bundled by espin, fascin, and espin + fascin compared to unmodified actin filaments (Fig. 7C). These observations, in combination with those observed in response to the experiments requested in #2d and #2e below, provide further insights into the underlying mechanisms of bundle disassembly.

2d. *"How do MICAL, espin, and fascin work alone or together to control cofilin loading on to F-actin? Cofilin severing activity is related to the fractional occupancy on the filament. Do the other 3 factors change the binding rules for cofilin?"*

We have now conducted experiments to address these questions. In particular, these questions center around if MICAL, espin, and/or fascin increase cofilin loading onto actin and therefore underlie cofilin's ability to assist/synergize with Mical in F-actin disassembly. Interestingly, previous work has found that cofilin loading onto F-actin is slowed by fascin (Breitsprecher et al., *Journal of Cell Science*, 2011; Chikireddy et al., *JCB*, 2024). Moreover, the reduction in cofilin binding is not because of the competitive binding between fascin and cofilin (Chikireddy et al., *JCB*, 2024). This happens likely because of the change in filament's helicity in fascin-bundled actin filaments (Claessens et al. (Bausch), *PNAS*, 2008; Lieleg et al., *Chemphyschem*, 2009; Gong et al., *Nat SMB*, 2025). Therefore, these results indicate that fascin does not assist Mical by increasing cofilin loading onto F-actin. Likewise, we find no evidence that espin assists Mical by increasing cofilin loading on to F-actin, since we find no difference in cofilin's binding to F-actin in the presence of espin concentrations used in TIRFM Mical+cofilin F-actin disassembly assays (Fig. S5E). So too, we have previously found that Mical alone (without its NADPH-dependent enzyme activity) does not affect cofilin's loading onto F-actin (Grintsevich et al., *NCB*, 2016; Rajan et al., *PNAS*, 2023). However, once Mical has oxidized F-actin, previous work reveals that cofilin binds better to

Mical-oxidized than unoxidized F-actin (Grintsevich et al., *NCB*, 2016; Wioland et al., *EMBO J*, 2021). Thus, together, these observations, plus other ones described in response to Questions #2c above and #2e below, present a model that Mical's oxidation of F-actin alters F-actin's conformation and stability – including weakening espin and fascin–F-actin bundling abilities (Fig. 7D (1)) – which increases cofilin's binding to filaments and allows it to disassemble Mical-oxidized filaments, which are already highly unstable, more rapidly than the unoxidized filaments (Fig. 7D (2-3)).

2e. *"Does MICAL or cofilin alter the fascin binding/unbinding dynamics seen in simpler systems (PMID: 20551315)? What about for espin?"*

We have now conducted experiments to address these questions. Previous work in PMID: 20551315 (Courson & Rock, *JBC*, 2010) related to fascin shows that: 1) actin bundles formed by fascin are extremely stable over time, and 2) the fascin in bundles is stable and does not dissociate from, or redistribute within, the bundles – unless competed away by free fascin or other bundling proteins (such as alpha-actinin). We therefore addressed the question of whether Mical or cofilin competed away fascin (altered fascin's binding/unbinding dynamics).

Related to if Mical alters fascin or espin binding/unbinding dynamics to F-actin: As discussed above in response to Question #2c, our new results reveal that there is no decrease in fascin-bundled F-actin or espin-bundled F-actin in the presence of Mical (in the absence of Mical's activating co-enzyme NADPH) (Fig. S5B). Thus, Mical, in the absence of its enzymatic activity does not alter fascin's binding/unbinding dynamics. However, we found that once Mical oxidizes actin filaments, although the affinity of fascin and espin for Mical-oxidized F-actin is similar to that for unoxidized F-actin (Fig. S5C; compare the Kapp in (1) and (2)), these Mical-oxidized actin filaments are more poorly bundled by espin, fascin, and espin + fascin compared to unmodified actin filaments (Fig. 7C). These results, coupled with previous results that Mical-oxidized F-actin is highly unstable (Hung et al., 2011; Grintsevich et al., 2016; Grintsevich et al., 2017) and Mical-oxidized G-actin only poorly polymerizes (Hung et al., 2011; Grintsevich et al., 2016), provide a biochemical understanding of how Mical disassembles complex bundled filaments. Namely, Mical, in the presence of its NADPH coenzyme, oxidizes actin within complex bundled filaments, and this destabilizes them, which leads to a loss of filaments that espin and fascin can bundle, as well as produces G-actin that cannot readily polymerize (Fig. 7D (1)).

Related to if cofilin alters fascin or espin binding/unbinding dynamics to F-actin: We have now included new data showing that at the concentrations of cofilin used in the Mical+cofilin disassembly assays (Figs. 6, S4), cofilin does not affect the binding of fascin or espin to F-actin (Fig. S5D). These results demonstrate that cofilin does not assist Mical-mediated bundled F-actin disassembly by preventing bundling proteins from interacting with F-actin (and see also Questions #2c-d above for more observations related to Mical and cofilin).

2f. *"Can a phase diagram be drawn that provides some mechanistic insight into what combinations and concentrations of factors favor bundle formation versus disintegration?"*

Yes, thank you, we have now provided a phase diagram that offers mechanistic insight into what combinations and concentrations of factors favor bundles formation versus disintegration (Fig. 9A).

2g. *"These sorts of experiments would help reveal mechanisms that go beyond phenotypic description."*

We thank the Reviewer for this comment. We hope that we have now better detailed how our studies – including: [1] finding molecules underlying complex bundles disassembly (Mical and cofilin), [2] determining how these bundles are disassembled (via shortening/depolymerization and severing/thinning), [3] determining the mechanisms through which Mical/Cofilin are disassembling these complex bundles (Mical's enzymatic actin oxidation activity and cofilin's synergy with Mical in inducing disassembly), and [4] showing that these mechanisms are also at play *in vivo* – go far beyond phenotypic description and provide a

molecular/biochemical/cellular conceptual understanding of how disassembly of complex bundled F-actin structures can occur.

<u>Minor points</u>:
<u>1.</u> *"The experiments are performed using preformed filaments and then adding bundling proteins. What happens during copolymerization? Which would better reflect the in vivo situation? Prepolymerize and then bundle, or copolymerize the actin in the presence of the bundling proteins?"*

We thank the Reviewer for this question. We have now clarified in the text why we conducted our experiments as we have. In particular, regarding the reviewer's question about which approach—[1] pre-polymerization followed by bundling or [2] co-polymerization with bundling proteins—better reflects the *in vivo* situation? Although the answer may vary depending on the specific cellular context, work in the bristle model (which is our *in vivo* model for these studies) indicates that filaments are formed and then bundled (e.g., Tilney and DeRosier, *PNAS*, 2005). We therefore performed our *in vitro* experiments with this in mind. This type of strategy also allows us to demonstrate that bundled filaments are disassembled versus not being formed.

It also should be noted that even though fascin does not affect actin elongation, espins, including the espin-4 splice variant, is known to decrease the rate of actin polymerization (Sekerková et al, *Journal of Neuroscience*, 2004, and our unpublished work). This reduction in polymerization rate results in shorter actin bundles compared to those formed in the presence of fascin. Therefore, to avoid potential bias in the analysis of bundles shortening, severing, and thinning rates due to differences in filaments/bundles length, we elongated the actin filaments before adding the bundling proteins. To maintain consistency, all experiments were performed following this same procedure.

<u>2.</u> "The in vivo work on bristles was very nice. Could more be done in the discussion to relate the in vitro work to the in vivo phenotypes and current models of bristle-stereocilia formation? Maybe not much more can be said, but I found myself interested to hear more from the authors to relate the current studies back to the historical context of the field. It's just a thought."

We thank the Reviewer for this supportive comment and interest. Although space limitations preclude us from extensively discussing these topics, we have now used the section of the Results describing our *in vivo* results and the Discussion section to more fully relate the *in vitro* work to the *in vivo* phenotypes and current models of bundle formation in bristle-stereocilia formation.

March 24, 2026

RE: JCB Manuscript #202509039R

Jonathan Terman
The University of Texas Southwestern Medical Center

Dear Dr. Terman:

Thank you for submitting your revised manuscript entitled "Synergistic Assembly, Disassembly, and Protection of Complex Forms of Bundled F-actin". The reviewers all now support publication so we would be happy to publish your paper in JCB pending final revisions necessary to meet our formatting guidelines (see details below).

To avoid unnecessary delays in the acceptance and publication of your paper, please read the following information carefully.

A. MANUSCRIPT ORGANIZATION AND FORMATTING:

Full guidelines are available on our Instructions for Authors page, http://jcb.rupress.org/submission-guidelines#revised.
Submission of a paper that does not conform to JCB guidelines will delay the acceptance of your manuscript.

1) Text limits: Character count for Articles is < 40,000, not including spaces. Count includes abstract, introduction, results, discussion, and acknowledgments. Count does not include title page, figure legends, materials and methods, references, tables, or supplemental legends.

2) Figures limits: Articles may have up to 10 main text figures.

**3) Figure formatting: Scale bars must be present on all microscopy images, including inset magnifications. Molecular weight or nucleic acid size markers must be included on all gel electrophoresis. Aspect ratios of images may not be altered.

**In order to accommodate readers with red-green color blindness, we ask that you please change the red/green color scheme used in the graphs throughout your figures, particularly in Figure 9.

**4) Statistical analysis: Error bars on graphic representations of numerical data must be clearly described in the figure legend. The number of independent data points (n) represented in a graph must be indicated in the legend.
**Statistical methods should be explained in full in the materials and methods. For figures presenting pooled data the statistical measure should be defined in the figure legends. Please also be sure to indicate the statistical tests used in each of your experiments (either in the figure legend itself or in a separate methods section) as well as the parameters of the test (for example, if you ran a t-test, please indicate if it was one- or two-sided, etc.). Also, if you used parametric tests, please indicate if the data distribution was tested for normality (and if so, how). If not, you must state something to the effect that "Data distribution was assumed to be normal but this was not formally tested."

5) Abstract and title: The abstract should be no longer than 160 words and should communicate the significance of the paper for a general audience. The title should be less than 100 characters including spaces. Make the title concise but accessible to a general readership.

**6) Materials and methods: Should be comprehensive and not simply reference a previous publication for details on how an experiment was performed. Please provide full descriptions in the text for readers who may not have access to referenced manuscripts. For example, in the "Protein Purification" paragraph of your methods section, you state: " Drosophila Fascin, Drosophila Mical (MicalRedoxCH), and human cofilin-1 (referred to as cofilin in this study) were purified as previously described (Hung et al., 2010; Rajan et al., 2023c)." You must remove this and write out the full methodology. The same goes for all other places in the methods where you refer to methods "...as previously described."

**7) All antibodies, cell lines, animals, and tools used in the manuscript should be described in full, including accession numbers for materials available in a public repository such as the Resource Identification Portal. Please be sure to provide the sequences for all of your primers/oligos and RNAi constructs in the materials and methods. You must also indicate in the methods the source, species, and catalog numbers (where appropriate) for all of your antibodies. Please also indicate the acquisition and quantification methods for immunoblotting/western blots.

**8) Microscope image acquisition: The following information must be provided about the acquisition and processing of images:
a. Make and model of microscope
b. Type, magnification, and numerical aperture of the objective lenses
c. Temperature

d. Imaging medium
e. Fluorochromes
f. Camera make and model
g. Acquisition software
h. Any software used for image processing subsequent to data acquisition. Please include details and types of operations involved (e.g., type of deconvolution, 3D reconstitutions, surface or volume rendering, gamma adjustments, etc.).

9) References: There is no limit to the number of references cited in a manuscript. References should be cited parenthetically in the text by author and year of publication. Abbreviate the names of journals according to PubMed.

10) Supplemental materials: There are strict limits on the allowable amount of supplemental data. Articles may have up to 5 supplemental figures. Please also note that tables, like figures, should be provided as individual, editable files. A summary of all supplemental material should appear at the end of the Materials and methods section.

11) eTOC summary: A ~40-50-word summary that describes the context and significance of the findings for a general readership should be included on the title page. The statement should be written in the present tense and refer to the work in the third person.

12) Conflict of interest statement: JCB requires inclusion of a statement in the acknowledgements regarding competing financial interests. If no competing financial interests exist, please include the following statement: "The authors declare no competing financial interests." If competing interests are declared, please follow your statement of these competing interests with the following statement: "The authors declare no further competing financial interests."

**13) ORCID IDs: ORCID IDs are unique identifiers allowing researchers to create a record of their various scholarly contributions in a single place. Please note that ORCID IDs are now *required* for all authors. At resubmission of your final files, please be sure to provide your ORCID ID and those of all co-authors.

14) A separate author contribution section following the Acknowledgments. All authors should be mentioned and designated by their full names. We encourage use of the CRediT nomenclature.

Please note that JCB now requires authors to submit Source Data used to generate figures containing gels and Western blots with all revised manuscripts. This Source Data consists of fully uncropped and unprocessed images for each gel/blot displayed in the main and supplemental figures. For assays performed using capillary electrophoresis and/or immunoassay-based detection, authors should instead provide the electropherogram graph(s) for each experiment, plotting fluorescence/chemiluminescence intensity vs. molecular weight/size. Please be sure to provide one Source Data file for each figure gels, blots, and/or capillary electrophoresis assays along with your revised manuscript files. File names for Source Data figures should be alphanumeric without any spaces or special characters (i.e., SourceDataF#, where F# refers to the associated main figure number or SourceDataFS# for those associated with Supplementary figures). For traditional gels and blots, the lanes of the gels/blots should be labeled as they are in the associated figure, the place where cropping was applied should be marked (with a box), and molecular weight/size standards should be labeled wherever possible. For capillary electrophoresis assays, each trace in the graph should be color-coded and labeled to indicate which protein, gene, or sample is being measured (please try to avoid red/green combinations to accommodate our color-blind readers).

Source Data files will be made available to reviewers during evaluation of revised manuscripts and, if your paper is eventually published in JCB, the files will be directly linked to specific figures in the published article.

Source Data Figures should be provided as individual PDF files (one file per figure). Authors should endeavor to retain a minimum resolution of 300 dpi or pixels per inch. Please review our instructions for export from Photoshop, Illustrator, and PowerPoint here: https://rupress.org/jcb/pages/submission-guidelines#revised

Journal of Cell Biology now requires a data availability statement for all research article submissions. These statements will be published in the article directly above the Acknowledgments. The statement should address all data underlying the research presented in the manuscript. Please visit the JCB instructions for authors for guidelines and examples of statements at (https://rupress.org/jcb/pages/editorial-policies#data-availability-statement).

B. FINAL FILES:

Please upload the following materials to our online submission system. These items are required prior to acceptance. If you have any questions, contact JCB's Managing Editor, Lindsey Hollander (lhollander@rockefeller.edu).

-- An editable version of the final text (.DOC or .DOCX) is needed for copyediting (no PDFs).

-- High-resolution figure and MP4 video files: See our detailed guidelines for preparing your production-ready images,

https://jcb.rupress.org/fig-vid-guidelines.

-- Cover images: If you have any striking images related to this story, we would be happy to consider them for inclusion on the journal cover. Submitted images may also be chosen for highlighting on the journal table of contents or JCB homepage carousel. Images should be uploaded as TIFF or EPS files and must be at least 300 dpi resolution.

It is JCB policy that if requested, original data images must be made available to the editors. Failure to provide original images upon request will result in unavoidable delays in publication. Please ensure that you have access to all original data images prior to final submission.

The license to publish form must be signed before your manuscript can be sent to production. A link to the license to publish form will be sent to the corresponding author only. Please take a moment to check your funder requirements before choosing the appropriate license.

Additionally, JCB encourages authors to submit a short video summary of their work. These videos are intended to convey the main messages of the study to a non-specialist, scientific audience. Think of them as an extended version of your abstract, or a short poster presentation. We encourage first authors to present the results to increase their visibility. The videos will be shared on social media to promote your work. For more detailed guidelines and tips on preparing your video, please visit https://rupress.org/jcb/pages/submission-guidelines#videoSummaries.

Thank you for your attention to these final processing requirements. Please revise and format the manuscript and upload materials within 7 days. If you need an extension for whatever reason, please let us know and we can work with you to determine a suitable revision period.

Please contact the journal office with any questions at cellbio@rockefeller.edu.

Thank you for this interesting contribution, we look forward to publishing your paper in Journal of Cell Biology.

Sincerely,

Pekka Lappalainen
Monitoring Editor
Journal of Cell Biology

Gabriele Stephan
Scientific Editor
Journal of Cell Biology

Reviewer #1:

The authors have improved their manuscript and have addressed all my concerns. I now recommend publication and congratulate the authors on this very nice work.

Reviewer #2:

The authors have thoughtfully addressed my comments, and the revised manuscript is substantially improved, particularly with respect to the underlying mechanisms of bundle disassembly. I believe this study will make a valuable contribution to the field.

Reviewer #3:

The authors have made strong revisions to improve the manuscript. I thank the authors for taking my comments seriously. I have no further concerns. This is solid work on an important question. Classic JCB material.
WMB
